# Blood Metabolite Signature of Metabolic Syndrome Implicates Alterations in Amino Acid Metabolism: Findings from the Baltimore Longitudinal Study of Aging (BLSA) and the Tsuruoka Metabolomics Cohort Study (TMCS)

**DOI:** 10.3390/ijms21041249

**Published:** 2020-02-13

**Authors:** Jackson A. Roberts, Vijay R. Varma, Chiung-Wei Huang, Yang An, Anup Oommen, Toshiko Tanaka, Luigi Ferrucci, Palchamy Elango, Toru Takebayashi, Sei Harada, Miho Iida, Madhav Thambisetty

**Affiliations:** 1 Clinical and Translational Neuroscience Section, Laboratory of Behavioral Neuroscience, National Institute on Aging, National Institutes of Health, Baltimore, MD 21224, USA; robertsjaa@nih.gov (J.A.R.); vijay.varma@nih.gov (V.R.V.); 2 Brain Aging and Behavior Section, Laboratory of Behavioral Neuroscience, National Institute on Aging, National Institutes of Health, Baltimore, MD 21224, USA; chiung-wei.huang@nih.gov (C.-W.H.); anya@grc.nia.nih.gov (Y.A.); 3 Glycoscience Group, National Centre for Biomedical Engineering Science, National University of Ireland Galway, Galway H91-TK33, Ireland; anup.mammen@gmail.com; 4 Translational Gerontology Branch, National Institute on Aging, NIH, Baltimore, MD 21224, USA; tanakato@mail.nih.gov (T.T.); ferruccilu@grc.nia.nih.gov (L.F.); elangop@mail.nih.gov (P.E.); 5 Department of Preventive Medicine and Public Health, Keio University, Tokyo 160-8282, Japan; ttakebayashi@a3.keio.jp (T.T.); seiharada@keio.jp (S.H.); mihoiida1029@gmail.com (M.I.)

**Keywords:** metabolic syndrome, metabolomics, amino acids, fasting glucose, triglycerides, waist circumference, blood pressure

## Abstract

Rapid lifestyle and dietary changes have contributed to a rise in the global prevalence of metabolic syndrome (MetS), which presents a potential healthcare crisis, owing to its association with an increased burden of multiple cardiovascular and neurological diseases. Prior work has identified the role that genetic, lifestyle, and environmental factors can play in the prevalence of MetS. Metabolomics is an important tool to study alterations in biochemical pathways intrinsic to the pathophysiology of MetS. We undertook a metabolomic study of MetS in serum samples from two ethnically distinct, well-characterized cohorts—the Baltimore Longitudinal Study of Aging (BLSA) from the U.S. and the Tsuruoka Metabolomics Cohort Study (TMCS) from Japan. We used multivariate logistic regression to identify metabolites that were associated with MetS in both cohorts. Among the top 25 most significant (lowest *p*-value) metabolite associations with MetS in each cohort, we identified 18 metabolites that were shared between TMCS and BLSA, the majority of which were classified as amino acids. These associations implicate multiple biochemical pathways in MetS, including branched-chain amino acid metabolism, glutathione production, aromatic amino acid metabolism, gluconeogenesis, and the tricarboxylic acid cycle. Our results suggest that fundamental alterations in amino acid metabolism may be central features of MetS.

## 1. Introduction 

Metabolic syndrome (MetS) is a cluster of risk factors that raises an individual’s risk for heart disease, diabetes, stroke, and chronic neurodegenerative disease [1,2,3,4]. MetS is defined as having 3 or more of 5 individual risk factors including elevated waist circumference, fasting serum/plasma glucose concentration, triglyceride levels, and blood pressure and reduced serum/plasma high-density lipoprotein (HDL) cholesterol [5]. In the United States, more than one-third of adults and more than one-half of those 60 years or older have MetS [6]. Additionally, the global spread of the Westernized lifestyle has increased the global burden of metabolic diseases, representing a substantial public health concern as more societies become sedentary and transition to more unhealthy diets [7,8]. In Japan, for instance, government estimates suggest that nearly one-third of males and one-tenth of females 20 years or older have MetS and expectations suggest that the prevalence will likely increase in the coming years [9]. Presence of MetS further inflates the per-patient cost of treatment for those with hypertension, and overall patient-specific health costs increase with the accumulation of each additional MetS risk factor [10]. 

Part of the health and cost burden imposed by MetS stems from its association with increased morbidity and mortality of other diseases. MetS has been shown to double the risk of fatal cardiovascular events [11]; has been shown to increase the risk of cardiovascular disease and type II diabetes mellitus (T2DM) [1,2]; and is associated with adverse neurological outcomes, including increased risk of cognitive decline, vascular dementia, and Alzheimer’s disease (AD) [12,13,14]. MetS likely increases risk of these conditions through chronic inflammation, hyperinsulinemia, dyslipidemia, and oxidative stress [15]. Identifying the biologic pathways and mechanisms underlying MetS is essential to developing biomarkers and interventions for multiple chronic and neurological diseases.

Despite prior work, the pathophysiology of MetS remains unclear. Recently, high-throughput omics has emerged as an important tool for understanding disease-specific cellular processes [16]. Metabolomics of blood in particular may improve the mechanistic understanding of disease pathology [17]. Metabolite profiling of MetS can help characterize altered metabolic processes, which may lead to earlier diagnosis and intervention as well as improved personalized treatments [18]. 

Prior work has identified blood serum metabolites that characterize early phenotypes of MetS prior to diagnosis [19]. Metabolomics has also been used to discriminate between overweight and obese individuals with and without MetS [20,21], suggesting some diagnostic utility. Other studies established blood metabolite profiles of different dietary patterns in patients with MetS [22,23], indicating that the approach may be useful for studying the impact of lifestyle on disease processes. Metabolomics studies in blood tissue have additionally identified multiple biochemical pathways in MetS, most notably altered amino acid metabolism [24]. 

Cross-cultural and multiethnic studies may yield significant insight into biochemical processes associated with MetS that are fundamental to the disease etiology. These studies may provide important information regarding the interaction between genes and environment in the development of disease and may shed light on fundamental biologic features of disease pathology. Prior cross-cultural studies have examined clustering of MetS risk factors by ethnicity [25] as well as shared and distinct metabolite associations with type II diabetes mellitus (T2DM) [26,27]. However, a metabolomic approach in two ethnically distinct longitudinal cohorts has not yet been applied to identify a metabolite signature of MetS.

The purpose of the present study is to comprehensively explore a serum metabolite signature of MetS in two ethnically distinct cohorts of older individuals—the Baltimore Longitudinal Study of Aging (BLSA) from the United States and Tsuruoka Metabolomics Cohort Study (TMCS) from Japan. These distinct cohorts allow for cross comparisons that can help identify metabolic alterations that may be fundamental to MetS. Specifically, we utilized capillary electrophoresis time-of-flight mass spectrometry (CE-TOFMS) to assay charged and hydrophilic blood serum metabolites and examined their associations with MetS. To our knowledge, our study is one of the first to utilize this omics platform in a large, multiethnic study of MetS.

## 2. Results

### 2.1. Participants

Table 1 summarizes the demographic characteristics of participants from the BLSA and TMCS cohorts. Comparing the overall samples between the two cohorts, as shown in columns 2 and 3 of Table 1, BLSA participants were, on average, older and more likely to have never smoked. The BLSA participants were more likely male compared to TMCS. The prevalence of MetS was similar between groups, but BLSA participants had a greater prevalence of elevated triglycerides and reduced HDL-C. Overall, TMCS participants had a greater prevalence of elevated waist circumference, elevated fasting glucose, and elevated blood pressure. Considering the continuous values for risk factors, on average, the BLSA cohort had a larger waist circumference and lower HDL cholesterol than the TMCS cohort. The TMCS cohort, on average, had higher fasting glucose and higher systolic and diastolic blood pressures. Average triglyceride levels did not differ between the two cohorts. Regarding prescription drug use, TMCS participants were more likely to use antihypertensive medications, while BLSA participants were more likely to use lipid-lowering medications. Both cohorts were similar in terms of use of diabetes medication.

**Figure 1:**
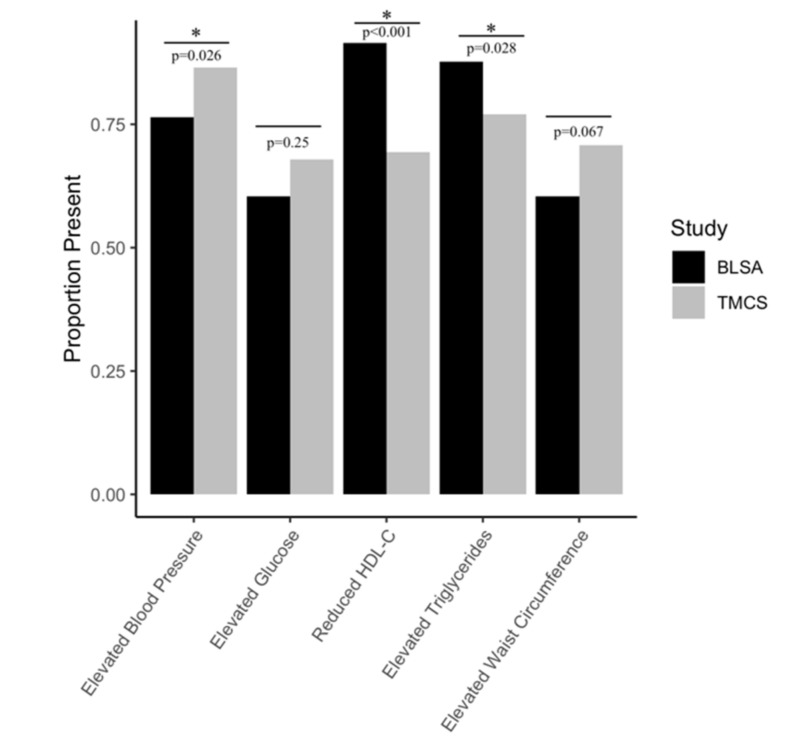
Distribution of individual MetS risk factors among participants with MetS in each cohort: * Significant difference at *p* < 0.05 between risk factor prevalence according to chi-square tests.

The comparisons between each cohort among individuals with MetS are displayed in columns 4 and 5 of Table 1 and are summarized in Figure 1. As shown in Figure 1, TMCS participants with MetS displayed a greater prevalence of elevated blood pressure, a lower prevalence of elevated triglycerides, and reduced HDL-C relative to BLSA participants with MetS. Similar to the comparison of the overall samples, individuals with MetS in the TMCS cohort displayed a higher use of antihypertensive medication. The use of diabetes medication and lipid-lowering medications was similar between the MetS subgroups of each cohort.

### 2.2. Metabolite Concentrations and Classifications

Table S2 includes the median metabolite concentrations for BLSA and TMCS and the false discovery rate (FDR)-adjusted *p*-values indicating the significance of differences between the two cohorts. The majority of metabolites (76/82) was significantly different after FDR correction between the two cohorts.

We classified metabolites according to the Human Metabolome Database (HMDB; http://www.hmdb.ca/) designations (see Table S1): 44 metabolites were classified as carboxylic acids and derivatives; 8 metabolites were classified as fatty acyls; 5 metabolites were classified as organooxygen compounds; 4 metabolites were classified as hydroxy acids and derivatives; 3 metabolites were classified as keto acids and derivatives; and 3 metabolites were classified as organonitrogen compounds. The additional 15 metabolites were each classified into distinct categories with two or fewer metabolites including amines, benzenes and substituted derivatives, glycerophospholipids, imidazopyrimidines, indoles and derivatives, organic sulfonic acids and derivatives, organic sulfuric acids and derivatives, pyridines and derivatives, and pyrimidine nucleosides. Three metabolites were unclassified by HMDB. 

### 2.3. Associations with Metabolic Risk Factors

In Table S3, we present the ranked results of all logistic regression models for both TMCS and BLSA cohorts separately. As described in the Methods section, we ranked metabolites based on the likelihood of a type I error (i.e., *p*-value) and identified the top 25 metabolites in each cohort with the lowest likelihood of a type I error (i.e., smallest *p*-value). We indicated with an * if a metabolite in the top 25 for one cohort was also in the top 25 for the other cohort, and we indicated with a † if the metabolite was unique to the top 25 in one cohort. In the BLSA, 17 metabolites were significantly associated with MetS, 16 were significantly associated with elevated waist circumference, 7 were significantly associated with elevated fasting glucose, 4 were significantly associated with elevated triglycerides, and no metabolites were significantly associated with reduced HDL-C or elevated blood pressure after FDR correction. In the TMCS cohort, 33 metabolites were significantly associated with MetS, 21 were significantly associated with elevated waist circumference, 21 were significantly associated with elevated triglycerides, 19 were associated with elevated fasting glucose, 6 were associated with reduced HDL-C, and 4 were associated with elevated blood pressure after FDR correction.

**Figure 2:**
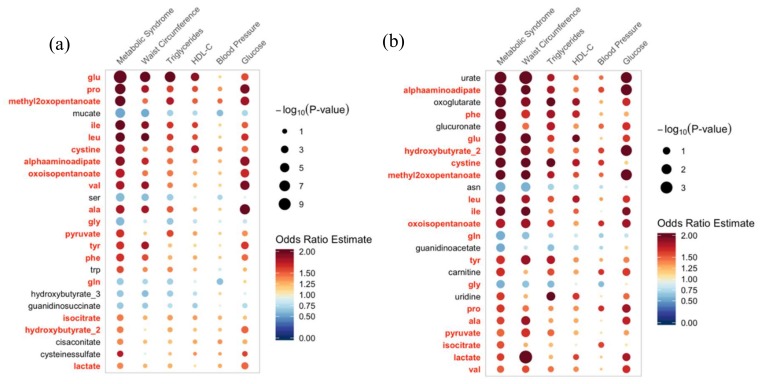
Logistic regression model results for the top 25 metabolites with the lowest likelihood of a type I error (i.e., smallest *p*-value) from the logistic regression model measuring the association between metabolites and MetS in (**a**) TMCS and (**b**) BLSA. *p*-values are false-discovery rate (FDR) corrected for multiple comparisons, and results are adjusted for age and sex in both cohorts as well as for storage time in the BLSA cohort. The *y*-axis indicates the serum metabolite concentration used as the predictor in the model and is ordered (i.e., ranked) from smallest to largest *p*-value within the top 25. The *x*-axis indicates the outcomes of MetS and its individual risk factors. The size of each circle corresponds to the significance of the association, with a larger circle indicating a smaller *p*-value. The color of each circle indicates the value of the odds ratio estimate with blue indicating an OR < 1 and red indicating an OR > 1. Red, bolded font indicates the metabolite was among the top 25 in both cohorts. Abbreviations: glu: glutamate; pro: proline; ile: isoleucine; leu: leucine; val: valine; ser: serine; ala: alanine; gly: glycine; tyr: tyrosine; phe: phenylalanine; trp: tryptophan; gln: glutamine; and asn: asparagine; BLSA: Baltimore Longitudinal Study of Aging; TMCS: Tsuruoka Metabolomics Cohort Study.

In Figure 2, we summarize the results of the logistic regression models measuring the association between metabolites and MetS in both cohorts for the 25 metabolites with the lowest likelihood of a type I error (i.e., smallest *p*-value). Of the 25 most significant metabolites, 18 metabolites were in the top 25 for both BLSA and TMCS cohorts while 7 were unique within each cohort. Results remained consistent in three different sensitivity analyses: (1) adjusting models for smoking status, physical activity, and diet quality (Table S4); (2) sex-stratified analyses (Table S5) in which fewer metabolites remained significant after FDR correction, likely due to reduced statistical power; and (3) excluding imputed values (Table S6).

### 2.4. Metabolite Classes Associated with MetS

A number of metabolite classes were represented among the 25 metabolites most significantly associated with MetS in each cohort. These were classified a priori using HMDB class designations reported above in Section 2.2. Of the 18 metabolites shared in the top 25 between cohorts, 13 represented carboxylic acids and their derivatives. The remaining 5 metabolites were keto acids and their derivatives (2 metabolites), hydroxy acids and their derivatives (2 metabolites), and pyruvate, which was unclassified by HMDB. The 7 metabolites unique to the TMCS cohort in the top 25 were classified as carboxylic acids and their derivatives (4 metabolites), hydroxy acid and their derivatives (1 metabolite), indole and their derivatives (1 metabolite), and organooxygen compounds (1 metabolite). The 7 metabolites unique to the BLSA cohort among the top 25 represented carboxylic acids and their derivatives (2 metabolites), pyrimidine nucleosides (1 metabolite), keto acid and their derivatives (1 metabolite), imidazopyrimidine (1 metabolite), organonitrogen compounds (1 metabolite), and organooxygen compounds (1 metabolite).

Of the 18 metabolites overlapping between BLSA and TMCS, we additionally categorized them into metabolic pathways based upon the HMDB pathway database and a literature search of metabolite-associated pathways (see the Methods section for a description of pathway classification relevant to MetS and related conditions). Classification by this methodology resulted in the identification of 7 primary pathways: aromatic amino acid metabolism, amino acid metabolism, lysine degradation, tricarboxylic acid cycle, glutathione production, gluconeogenesis, and branched-chain amino acid metabolism. The class and primary pathways of the 18 metabolites overlapping between BLSA and TMCS in the top 25 are summarized in Table 2. 

## 3. Discussion

In this study, we compared U.S. and Japanese populations of older individuals in order to identify a serum metabolite signature of MetS that may be intrinsic to the syndrome despite markedly different population-specific characteristics and distribution of individual risk factors. We identified 18 metabolites that were common among the 25 metabolites with the strongest associations with MetS in each cohort; these metabolites were mainly carboxylic acids, hydroxy acids, and keto acids. Overwhelmingly, the shared associations identified in this study represented amino acids—which account for 11 of the 13 carboxylic acids among the 18 shared metabolites—or metabolites implicated in amino acid metabolism. Despite numerous differences between cohorts including metabolite concentrations and prevalence of MetS risk factors, we identified alterations in overlapping metabolites across cohorts, suggesting that fundamental alterations in amino acid metabolism may be intrinsic to MetS pathophysiology. This signature suggests a set of metabolites and associated pathways that may be important for improved diagnosis and targeted therapeutic approaches.

Interestingly, while the prevalence of MetS was similar between TMCS and BLSA cohorts, the prevalence of individual risk factors in the overall sample as well as among individuals with MetS varied between cohorts. This finding is consistent with prior studies that indicate a cross-cultural heterogeneity in the combination of the risk factors in MetS [25]. Part of these differences in risk factor prevalence may stem from differences in drug prescription guidelines and practice between the U.S. and Japan, since we reported differences in drug usage prevalence between cohorts. It has previously been reported that cholesterol-lowering statins are underutilized in Japanese patients at risk for cardiovascular disease, and differences in antihypertensive medication prescription have been reported, suggesting that drug prescription guidelines and practices could underly this difference [28,29,30]. It is important to note that we do not report pretreatment values of these measures and are therefore unable to identify with certainty how differences in drug usage may impact risk factor differences. Finally, contrary to prior research indicating a higher smoking prevalence in Asian countries [31], we report higher rates of smoking among U.S. participants in this study.

Our findings suggest that, despite the heterogeneity in the prevalence of MetS risk factors across both cohorts and differences in serum concentrations in over 92% of the metabolites measured in this study, the majority of each cohort’s most significant metabolite associations with MetS was similar. The 18 common and overlapping metabolites were either amino acids or metabolites directly or indirectly related to amino acid metabolism, suggesting that amino acid metabolism and associated pathways may be fundamental to the biologic processes that may underlie MetS. In particular, the amino acid metabolic pathways represented in these results potentially implicate branched-chain amino acid (BCAA) metabolism and glutathione (GSH) synthesis in the pathogenesis of MetS.

Circulating concentrations of both serum and plasma BCAAs, which include valine, isoleucine, and leucine, have been previously linked to obesity and insulin resistance [32,33,34]. In a Chinese cohort, BCAAs were associated with overall MetS as well as elevated fasting glucose, elevated triglycerides, and reduced HDL-C [35]. When BCAA metabolism becomes dysregulated, as in MetS, the breakdown products of valine, isoleucine, and leucine may accumulate and exert negative metabolic effects. This is evidenced by the previous finding that a genetic disposition to impaired BCAA metabolism confers an increased risk for type II diabetes mellitus [36]. Notably, branched-chain alpha-ketoacid (BCKA; direct catabolic products of the BCAAs) administration has been demonstrated to induce mitochondrial dysfunction in diverse tissues [37,38,39]. In our study, all three BCAAs were positively associated with MetS in both cohorts and the BCKAs methyl-2-oxopentanoate and oxoisopentanoate were also positively associated with MetS. This suggests that BCAA metabolism may be fundamentally implicated in MetS and confirms that BCAAs and BCKAs may represent markers of the disease in diverse populations. Interestingly, both BCAAs and BCKAs have been associated with MetS in a previous TMCS cohort of postmenopausal women [40].

More generally, BCAAs have been frequently studied in the context of metabolic disorders and obesity. Among healthy-weight individuals, BCAAs appear to exert beneficial effects, including a decreased risk of obesity, increased muscle mass, potential improvements in glucose sensitivity, and possible therapeutic effects for patients with hepatic cirrhosis and encephalopathy [41,42,43,44]. However, long-term exposure to elevated BCAAs stimulates hyperphagia and obesity, has been correlated positively with LDL and triglyceride levels and negatively with HDL-C, and directly inhibits the TCA cycle through BCKA accumulation [43,45,46]. In prior in vitro studies using rat cortical slices, BCKA accumulation has also been demonstrated to reduce neuronal glutamate uptake [47], which has been identified as a potential pathological feature of Alzheimer’s disease (AD) [48]. In addition, a genetic predisposition to elevated isoleucine has been found to increase risk of AD, and leucine has previously been shown to promote tau accumulation via a mammalian target of rapamycin (mTOR)-dependent mechanism [49,50]. An altered BCAA metabolism could therefore represent a potential mechanistic link between MetS and AD [51].

Oxidative stress likely plays a key role in the pathology of both AD and obesity-related disorders [52,53,54] among older adult populations like those included in this study. One essential mechanism for combatting oxidative stress is the production of GSH, a molecule with numerous antioxidant roles for which homeostasis is disrupted in both AD and obesity [55,56]. Interestingly, our recent work using the CE-MS platform to measure brain tissue metabolite concentrations in the BLSA autopsy study identified a positive association between GSH and cystine, a GSH precursor, in AD, suggesting a disease-specific, chronic inflammatory state [57]. In the present study, several amino acids essential to GSH synthesis were associated with MetS. Most notably, cystine and glutamate displayed positive associations with MetS while glycine displayed a negative association. While cystine is considered a rate-limiting metabolite for the synthesis of GSH, elevated extracellular glutamate inhibits the uptake of cystine and reduces GSH synthesis [58]. Independently, cystine has been associated with obesity and insulin resistance [59]. Additionally, 2-hydroxybutyrate was positively associated with MetS in this study and plays an important role in the GSH synthetic pathway. Specifically, 2-hydroxybutyrate is a byproduct of cystine formation, and its association with MetS may represent an increased flux through GSH synthesis in response to oxidative stress [60]. When its levels are reduced, glycine may become the limiting factor in GSH production as suggested by the finding that glycine supplementation restores GSH levels and reduces oxidative stress [61]. Serine, which was associated with a decreased risk of MetS in the TMCS cohort only, has been shown to play a role in GSH synthesis through its conversion to glycine [62]. Another amino acid, glutamine, that was associated with a decreased risk of MetS in both cohorts has additionally been shown to drive production of glutathione [63]. As a whole, our results suggest that MetS implicates pathways associated with chronic oxidative stress owing to the positive associations of several metabolites from the GSH synthesis pathway with MetS. Furthermore, glycine availability may be a limiting factor in GSH production under these conditions, since glycine demonstrated a negative association with MetS.

Our results further implicate the importance of gluconeogenesis and particularly the glucose-alanine cycle in MetS. In this study, alanine, pyruvate, and lactate all displayed positive associations with MetS across cohorts. Alanine is the predominant glucogenic amino acid and is typically released from muscle under conditions in which glucose is scarce. In peripheral tissues, alanine is produced through transamination of pyruvate and is then converted back to pyruvate and glucose via gluconeogenesis in the liver [64]. Lactate is typically produced from pyruvate under conditions of anaerobic glycolysis and is similarly reconverted to pyruvate for gluconeogenesis in the liver. Subcutaneous fat has been demonstrated to be a source of excess lactate in obese individuals, suggesting altered metabolism in the obese [65]. The positive associations of alanine, pyruvate, and lactate with MetS suggests that alterations in glycolytic flux are likely central features of MetS.

Interestingly, it has been shown that disruption of pyruvate entry into the tricarboxylic acid (TCA) cycle causes an increased rate of conversion of glutamine—which was identified as protective against MetS in this study—into oxoglutarate, a TCA cycle intermediate [66]. Increased utilization of amino acids for the TCA cycle under pathological conditions could reduce their availability for other key biochemical pathways, such as GSH synthesis discussed previously. More general alterations in the TCA cycle may be fundamental to MetS, as we identified that isocitrate was positively associated with the disease in both cohorts. This is not surprising, as increased anaplerotic flux generating TCA intermediates is a hallmark feature of obesity and insulin resistance [67].

We additionally identified an alteration in aromatic amino acid metabolism, indicated by the positive association of phenylalanine and tyrosine with MetS across cohorts. Tyrosine is produced through hydroxylation of phenylalanine, and both metabolites have previously been associated with obesity [68]. These amino acids may become elevated as a result of competition with BCAAs for the same large neutral amino acid transporter or inhibition of tyrosine aminotransferase by cystine [32,69]. Interestingly, phenylalanine and tyrosine function as precursors for catecholamines in the brain, presenting a potential link between the metabolic dysfunction of MetS and common neurological diseases [70,71].

Lastly, we identified that alpha-aminoadipate, a breakdown product of lysine degradation, was associated with MetS in both cohorts. This metabolite has previously been identified as a marker distinguishing between metabolically healthy and unhealthy obese individuals and has been considered a biomarker of diabetes [72,73].

In a prior study, we utilized metabolomics to identify classes of metabolites associated with MetS in a study including similar cohorts from the BLSA and TMCS [74]. In that study, we performed flow injection analysis-tandem mass spectrometry and liquid chromatography-tandem mass spectrometry to quantify 167 metabolites spanning a wide variety of polar and hydrophobic compounds. We identified that phosphatidylcholines-acyl-alkyls, sphingomyelins, and hexoses were associated with MetS in both cohorts, suggesting that these classes of metabolites may represent fundamental markers of the syndrome. Furthermore, we measured 27 of the 82 metabolites quantified in this study, some of which were associated with MetS in this study but not our prior work. There may be a few explanations for this disparity between our results. First, the present study makes use of much larger sample sizes for both cohorts, nearly six times larger for TMCS and twice as large for BLSA. This increased the statistical power of our analyses and thus improved the possibility that additional metabolite associations were captured. In addition, the current study utilizes a different metabolomics platform than our previous work, quantifying metabolites by CE-TOFMS as opposed to LC-MS, which may explain some variation in our results.

Our study has several limitations. First, the TMCS cohort represented a narrower age range than the BLSA cohort and were from the same city and prefecture in Japan, suggesting that the BLSA cohort was more heterogenous with respect to a number of unmeasured environmental and ethnicity-related factors. Additionally, the TMCS cohort is likely more representative of the general Japanese population than the BLSA cohort is of the U.S. population, since the BLSA participants are predominantly Caucasian, well educated, and relatively healthy. In addition, the metabolomics platform we utilized only allowed us to measure a subset of the serum metabolome. Furthermore, while our sample provided sufficient power to detect significant associations between metabolites and MetS, a larger sample size may have allowed for the detection of other novel metabolites that we did not detect in our current analyses. Lastly, the cross-sectional nature of the present study prevents us from making inferences regarding metabolic predictors of MetS onset or how longitudinal changes in metabolite concentrations may reflect disease processes.

In conclusion, our results highlight the importance of several altered amino acid metabolic pathways in MetS. Specifically, we confirm through analysis in two ethnically distinct cohorts that BCAA metabolism and GSH synthesis may represent central pathways contributing to MetS and additionally note alterations in gluconeogenesis, TCA cycle, aromatic amino acid metabolism, and lysine degradation. It is possible that these alterations in amino acid metabolism may be the result of mitochondrial dysfunction coupled with protein breakdown, which have previously been discussed as causal mechanisms in amino acid alterations [68,75]. These alterations, however, take place across disparate biochemical pathways, suggesting that MetS likely represents a state of chronic, systemic metabolic dysregulation. The findings of this study are relevant for future studies examining altered metabolic flux through these pathways in MetS as well as an understanding of shared pathways across MetS and other diseases associated with aging.

## 4. Methods

### 4.1. Participants

The National Institute on Aging’s (NIA) BLSA is one of the longest running scientific studies of human aging in the U.S. [76]. This observational study began in 1958 and includes longitudinal clinical, radiological, and laboratory evaluations on community-dwelling volunteer participants. The individuals in this study were participants in the neuroimaging sub-study of the BLSA [77]. Written informed consent was obtained at each visit for all BLSA participants. The BLSA study protocol has ongoing approval from the Institutional Review Board of the National Institute of Environmental Health Science, National Institutes of Health (“Early Markers of Alzheimer’s Disease (BLSA)”, IRB No. 2009-074).

The Tsuruoka Metabolomics Cohort Study (TMCS) is a population-based study of residents from Tsuruoka City, Yamagata Prefecture, Japan that began in 2012. Participants were recruited from annual municipal or worksite health checkup programs in the city. The individuals in this study were a subset from the baseline survey participants of the TMCS [78]. Written informed consent was obtained from all TMCS participants. The TMCS study protocol has ongoing approval from the Medical Ethics Committee of the School of Medicine, Keio University, Tokyo, Japan (Approval No. 20110264; original approval date: 6 December 2011; latest update: 2 December 2019).

### 4.2. Blood Samples

In BLSA participants, blood samples were collected at the NIA Clinical Research Unit in Harbor Hospital, Baltimore, MD. Collection and processing details have been described previously [79]. Briefly, venous blood samples were collected (after an overnight fast) between 6 and 7 AM. Serum samples were aliquoted into 0.5-mL volumes in Nunc cryogenic tubes (Rochester, NY, USA) and stored at −80 °C. Samples were not subject to any freeze–thaw cycles prior to metabolomic assays. The average storage time of BLSA serum samples prior to thaw for quantitative metabolomics was 12.39 years (SD: 9.41). The sample included 252 participants.

For TMCS participants, blood serum samples were collected during annual health checkups. Details on collection and processing have been published previously [40,78]. Briefly, venous blood samples were collected (after an overnight fast) between 8:30 and 10:30 AM. Serum samples were collected with serum-separating medium and assayed immediately. Storage time was less than 6 h and did not vary across TMCS participants. The sample included 644 participants.

### 4.3. Metabolites

Non-targeted metabolomics was carried out in this study using capillary electrophoresis time-of-flight mass spectrometry (CE-TOFMS) for quantification of metabolites. CE-TOFMS captures a broad range of charged and hydrophilic metabolites, including amino acids, organic acids, ketoacids, and a number of other metabolite classes which represent a variety of biological pathways. Compared to other methods, CE-TOFMS requires a small sample size with robust sensitivity and high resolution of results [80].

#### 4.3.1. Metabolite Extraction

As described previously [81], metabolite extraction was completed at Keio University, Tokyo, Japan. Briefly, samples were thawed and 100-μL serum aliquots were placed in 900-μL of methanol with internal standards (20 μmol of both methionine sulfur and camphor 10-sulfonic acid). Solutions were mixed and 400 μL of Milli-Q water (Millipore, Billerica, MA, USA) and 1 mL of chloroform was added, followed by centrifugation at 4600× *g* for 5 min at 4 °C. Next, the aqueous layer was transferred to a 5-kDa cutoff centrifugal filter tube (Millipore, Billerica, MA, USA) to remove large molecules. The filtrate was then centrifugally concentrated at 35 °C and reconstituted with 50 μL of Milli-Q water that contained reference compounds (200 μmoL/L each of 3-aminopyrrolidine and trimesic acid) prior to CE-TOFMS.

#### 4.3.2. Capillary Electrophoresis Time-of-Flight Mass Spectrometry (CE-TOFMS)

Analysis using CE-TOFMS to quantify cationic and anionic metabolites has been described previously [82,83,84]. Briefly, CE-TOFMS analysis was used an Agilent CE capillary electrophoresis system equipped with an Agilent 6210 time-of-flight mass spectrometer, Agilent 1100 series isocratic HPLC pump, Agilent G1603A CE-MS adapter kit, and Agilent G1607A CE-ESI-MS sprayer kit (Agilent Technologies, Waldbronn, Germany). Agilent G2201AA ChemStation software version B.03.01 for CE (Agilent Technologies, Waldbronn, Germany) was used to control systems. Systems were connected by a fused silica capillary (50 μm internal diameter × 100 cm total length) with 1 mol/L formic acid as the electrolyte for cationic analysis and 50 mmol/L ammonium acetate solution for anionic analysis. The spectrometer was scanned from *m/z* 50 to 1000 [85]. MasterHands automatic integration software (Keio University, Tsuruoka, Yamagata, Japan) was used to extract peaks in order to quantify *m/z*, peak area, and migration time (MT) [84]. Signal peaks corresponding to isotopomers, adduct ions, and other product ions of known metabolites were excluded. The remaining peaks were annotated by matching *m/z* values and normalized migration times of corresponding authentic standard compounds [81].

As described previously [86], CE-TOFMS analysis enabled measurement of the absolute concentrations of metabolites on the basis of their peak area and a 6-point calibration curve for each metabolite. Quantification was performed using the (M+H)^+^ or (M-H)^−^ parent ion peak area for each metabolite compared to the same parent peak in the standard solution. Concentration is only reported if the measured area is above the signal:noise ratio (S/N) of 5 and peak area is within linear range of the standard curve [87]. As reported previously [88], coefficients of variation (CVs) for repeat technical was less than 10%. In this study, 103 distinct metabolites were originally detected by CE-TOFMS. Metabolite concentrations are reported in µM.

### 4.4. Metabolite Classifications

**Figure 3:**
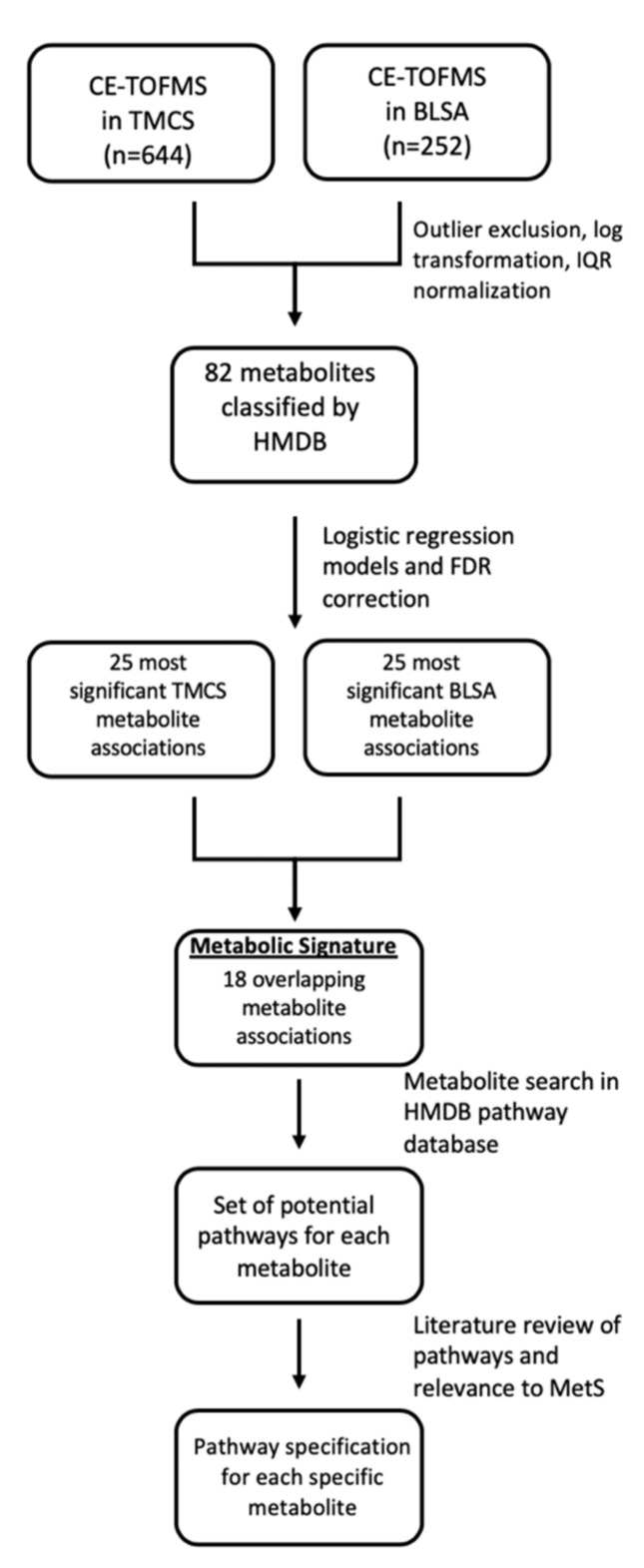
Establishing a metabolic signature of MetS: This figure presents an overview of the methodology, demonstrating data processing steps, analytic methods, and data interpretation including class and pathway classification of metabolites. CE-TOFMS: capillary electrophoresis time-of-flight mass spectrometry; TMCS: Tsuruoka Metabolomics Cohort Study; BLSA: Baltimore Longitudinal Study of Aging; HMDB: Human Metabolome Database; FDR: false-discovery rate.

Among the 82 metabolites included in analyses, we identified metabolite classes and primary pathways using a three-step process (summarized in Figure 3). We first categorized metabolites based on the Human Metabolome Database (HMDB) designation of metabolite class, subclass, and direct parent [89]. Table S1 includes classifications and groupings of all metabolites. The “class” classification is included in the tables in the Results section for significant metabolites.

Second, after identifying the 18 overlapping metabolites associated with MetS in both cohorts, we used the HMDB pathway database to identify the biochemical pathways each individual metabolite participates in as well as the pathways that were overlapping across metabolites. Third, we conducted a comprehensive literature search to identify which of the overlapping pathways were associated with MetS and/or related conditions. The primary pathway for metabolites highlighted in the tables in the Results section for significant metabolites included the pathways identified by HMDB with primary biologic relevance to MetS.

### 4.5. Outcomes

#### 4.5.1. Definition of MetS

Metabolic syndrome (MetS) was defined using the Third Adults Treatment Panel of the National Cholesterol Education Program (NCEPATPIII) criteria, revised by the American Heart Association and National Heart, Lung, and Blood Institute (AHA/NHLBI) [5]. A diagnosis of MetS required meeting at least three of five criteria/risk factors, including elevated waist circumference, elevated fasting plasma glucose, elevated serum triglyceride levels, reduced serum HDL cholesterol, and elevated blood pressure. Elevated waist circumference was defined as greater than 40 inches in males and greater than 35 inches in females. For TMCS participants, the criterion was modified as appropriate for a Japanese population, defining elevated waist circumference as greater than or equal to 100 cm in males and greater than or equal to 90 cm in females [40]. Elevated fasting glucose was defined as greater than or equal to 100 mg/dL or the use of prescription diabetes medications. Elevated triglyceride level was defined as greater than or equal to 150 mg/Dl or the use of prescription hyperlipidemia medications. Reduced HDL cholesterol was defined as less than 40 mg/dl in males, less than 50 mg/dL in females, or the use of prescription hyperlipidemia medications. Elevated blood pressure was defined as greater than or equal to 130 mm Hg systolic blood pressure, greater than or equal to 85 mm Hg diastolic blood pressure, or the use of prescription hypertension medications. All prescription medication use in both cohorts were self-reported through a standardized medication questionnaire.

#### 4.5.2. Individual Risk Factors

In the BLSA sample, plasma levels of triglycerides and fasting plasma glucose were measured using previously detailed enzymatic methods [90,91]. HDL-C values were measured using a dextran sulfate-magnesium precipitation procedure. In addition to outcomes measured in blood serum, waist circumference and blood pressure in both cohorts were also measured. BLSA staff clinicians measured waist circumference with a tape measure kept parallel to the floor from the hipbone and wrapping around the waist at the level of the umbilicus while participants held their breath [92]. Systolic and diastolic blood pressures were recorded three times in both arms in a seated position using a mercury sphygmomanometer sized to the arm of each participant, and the mean of the systolic and diastolic measurements were used in analysis [93].

In the TMCS sample, serum levels of triglycerides and fasting plasma glucose were measured using enzymatic and hexokinase methods, respectively [94]. HDL-C values were obtained using a direct method. Waist circumference was measured to the nearest 0.1 cm at the umbilicus at the end of a normal breath. If the umbilicus drooped down, the measurement was recorded midway between the inferior margin of the last rib and the top of the iliac crest in a horizontal plane [40]. Systolic and diastolic blood pressures were each measured twice on one occasion while seated using an automated sphygmomanometer (Omron HBP-T105S-N, Kyoto, Kyoto, Japan), and the mean of each of the two measurements were used in analysis [40].

### 4.6. Statistical Analyses

We first excluded all metabolites with >30% missing values (i.e., values identified as “not detectable” (ND) due to values lower than the limit of detection (LOD)) [57] and only included metabolites that overlapped between samples; 21 of 103 metabolites were excluded, resulting in 82 total metabolites included in analyses (see Figure 3). Similar to prior work, we then imputed all missing values as the lowest detectable value/2 [95]. In the BLSA cohort, the number of imputed values for individual metabolites ranged from 0 (0.0% of total) to 54 (21.4% of total), with a median of 5 (2.0% of total). In the TMCS cohort, the number of imputed values ranged from 0 (0.0% total) to 174 (27.0% of total), with a median of 12 (1.9% of total). Following imputation, we compared metabolite values between BLSA and TMCS using nonparametric Wilcoxon rank-sum tests. To account for multiple comparisons, we corrected levels of significance using false discovery rate (FDR)-adjusted *p*-values [96]. We compared demographic characteristics in addition to the prevalence of MetS and its individual risk factors between BLSA and TMCS cohorts using two-sample t-tests for continuous variables and chi-squared tests for categorical variables.

For primary analyses, we first natural log transformed all 82 metabolites and then excluded outlier values outside the 1.5 interquartile range (i.e., 1.5 × IQR). We then performed IQR normalization of metabolite values by subtracting the median value from the individual metabolite value and by dividing by the IQR. We used these normalized values in multivariate logistic regression models to explore the associations between distinct blood metabolites (predictors) and the binary outcome indicators of MetS (i.e., present/absent) and its 5 individual risk factors: elevated waist circumference, elevated fasting glucose, elevated triglycerides, reduced HDL cholesterol, and elevated blood pressure. The logistic regression models indicated whether each of the 82 metabolites was associated with either increased or decreased odds of the outcome. Covariates included sex and age at blood draw. For BLSA models, we included storage time as an additional covariate. Model results indicate an increase or decrease in the odds of having MetS or one of its individual risk factors associated with a one-unit increase in the normalized metabolite concentration(s). We corrected levels of significance using FDR-adjusted *p*-values using the same method described previously.

In order to identify a metabolite signature of MetS and to then describe the biologic pathways that may link the metabolites to underlying disease processes, we first ranked metabolites within each cohort. The ranking was based on the likelihood of a type I error (e.g., *p*-value) generated from the logistic regression model, indicating the strength of the association between the metabolite and MetS. The highest-ranking metabolite had the least likelihood of a type I error (i.e., smallest FDR-corrected *p*-value), the second-highest-ranking metabolite had the second smallest *p*-value, and so on. We used this ranking to identify the top 25 metabolites in each cohort. We then visualized the 25 top ranked metabolites for each cohort using side-by-side rainplots [97], which visualize the significance and direction of the odds ratio estimate of each metabolite association. Of these 25 metabolites, we identified which metabolites were shared between cohorts (i.e., in the top 25 for both BLSA and TMCS), which suggest metabolites and associated metabolic pathways that may be intrinsic to MetS despite differences in population-specific factors. We additionally identified metabolites that were unique to a cohort (i.e., in the top 25 for one cohort but not in the other). We included both the a priori indicated HMDB designated “class” classification as well as the primary metabolic “pathway” classification in order to meaningfully group metabolites and to develop a model linking metabolite associations to biological processes.

We performed sensitivity analyses for the multivariate logistic regression models including smoking status, physical activity, and diet (for TMCS only) as covariates in addition to age, sex, and serum sample storage time (for BLSA only). Smoking status was coded 0 (never smoker) or 1 (current or former smoker). Diet quality was assessed using the Dietary Approaches to Stop Hypertension (DASH) score [98]. The DASH score indicates adherence to the DASH dietary pattern by measuring consumption of 9 target nutrients including total fat, saturated fat, protein, fiber, cholesterol, calcium, potassium, and magnesium. We excluded magnesium as this target was not included in TMCS data collection. The DASH score therefore represented the sum of 8 nutrient components, with higher values indicating a higher quality diet. The DASH score was not collected for the majority of BLSA participants included in this study and was therefore only included in sensitivity analyses for the TMCS cohort.

Physical activity measurements were obtained via questionnaire in each study. For BLSA participants, a physical activity (PA) questionnaire was administered that asked participants to estimate the time spent performing 97 different activities. The use of this questionnaire in the BLSA has previously been described [99]. PA intensity was measured in metabolic equivalents (METs) [100]. As in prior studies, activities were grouped into low-intensity (<4 METs), moderate-intensity (4–5.9 METs), and high-intensity (>6 METs) [101]. Total PA was determined by summing the three categories of activity and by multiplying the hours spent in each activity by the assigned MET value. The PA survey administered to TMCS participants was developed initially for the Japan Public Health Center-based Study [102] and relies on measurement of the domains of occupational activity, leisure time activity, sleeping, and other activities [103]. Total PA was determined by summing all domains of activity and by multiplying the hours spent in each activity by the assigned MET value. In both BLSA and TMCS, we used total PA in METs/week in sensitivity analyses.

We conducted additional sensitivity analyses exploring potential sex-specific differences in metabolite associations. We ran sex-stratified multivariate logistic regression models for the BLSA and TMCS including age as a covariate as well as sample storage time in the BLSA cohort. Finally, we conducted sensitivity analyses to verify that imputation did not significantly impact results. We ran multivariate logistic regression models for BLSA and TMCS including sex and age as covariates as well as sample storage time in the BLSA cohort while excluding all imputed data from the analyses.

For all logistic regression models, we reported odds ratios. The type I error level was set to 0.05 for unadjusted *p*-values, and we corrected for multiple comparisons using FDR-adjusted *p*-values as described above. We used R Studio 1.1.453 for all data analyses and visualizations.

## Figures and Tables

**Table 1:** Demographic characteristics of the Baltimore Longitudinal Study of Aging (BLSA) and Tsuruoka Metabolomics Cohort Study (TMCS) cohorts for overall samples and only participants with metabolic syndrome (MetS): Two sample t-tests were used for comparison of continuous variables. Chi-square tests were utilized for comparison of categorical variables. * Significant difference between cohorts at *p* < 0.05 in the full sample and † comparisons were significant at *p* < 0.05 in samples restricted to participants with MetS. Physical activity questionnaires were not directly comparable, and thus, between group differences were not calculated.

	BLSA (Total Sample) (*n* = 252)	TMCS (Total Sample) (*n* = 644)	BLSA MetS (*n* = 106)	TMCS MetS (*n* = 274)
Age, Mean (SD)	73.8 (8.8) *	69.4 (2.2) *	72.6 (7.9) ^†^	69.4 (2.3) ^†^
Female, n (%)	120 (47.2) *	359 (55.8) *	46 (43.4) ^†^	190 (69.3) ^†^
White, n (%)	210 (83.3)	-	82 (77.4)	-
Never smoke, n (%)	112 (44.4) *	424 (65.8) *	60 (56.6) ^†^	205 (74.8) ^†^
Physical Activity (SD)	87.3 (61.5)	17.5 (14.6)	80.6 (58.8)	16.8 (13.5)
DASH Score (SD)	-	2.69 (0.7)	-	2.71 (0.7)
Storage time, Mean (SD)	12.4 (9.4)	-	11.1 (9.2)	-
Metabolic syndrome, n (%)	106 (42.1)	274 (42.6)	-	-
Elevated waist circumference, n (%)	83 (32.9) *	276 (42.9) *	64 (60.4)	194 (70.8)
Waist circumference, Mean (SD)	35.9 (4.8) *	32.6 (3.2) *	38.4 (4.4) ^†^	34.2 (3.2) ^†^
Elevated triglyceride level, n (%)	121 (48.0) *	241 (37.4) *	93 (87.7) ^†^	211 (77.0) ^†^
Triglyceride level, Mean (SD)	104.5 (59.6)	105 (62.4)	133.7 (73.5)	124.6 (79.1)
Hyperlipidemia drug use, n (%)	93 (37.4) *	187 (29.0) *	70 (66.0)	175 (63.9)
Reduced HDL cholesterol, n (%)	138 (54.8) *	209 (32.4) *	97 (91.5) ^†^	190 (69.3) ^†^
HDL cholesterol, Mean (SD)	56.1 (16.4) *	67.4 (18.0) *	49.6 (14.6) ^†^	64.3 (18.7) ^†^
Elevated Blood Pressure, n (%)	134 (53.2) *	453 (70.3) *	81 (76.4) ^†^	237 (86.5) ^†^
SBP, Mean (SD)	124.6 (20.2) *	132.8 (18.4) *	125.3 (18.9) ^†^	137.4 (18.0) ^†^
DBP, Mean (SD)	71.3 (12.3) *	75.6 (10.7) *	71.8 (12.7) ^†^	76.9 (10.0) ^†^
Hypertension drug use, n (%)	56 (22.5) *	297 (46.1) *	42 (39.6) ^†^	165 (60.2) ^†^
Elevated fasting glucose, n (%)	97 (39.0) *	319 (49.5) *	64 (60.4)	186 (67.9)
Fasting glucose, Mean (SD)	99.6 (16.7) *	103.2 (15.9) *	106.5 (19.9)	107.1 (17.1)
Diabetes drug use, n (%)	18 (7.2)	60 (9.3)	17 (16.0)	38 (13.9)

BLSA: Baltimore Longitudinal Study of Aging; TMCS: Tsuruoka Metabolomics Cohort Study; SBP: systolic blood pressure; DBP: diastolic blood pressure; SD: standard deviation; DASH: Dietary Approaches to Stop Hypertension; HDL: high-density lipoprotein.

**Table 2:** Shared metabolites among the top 25 metabolites with the lowest probability of type I error from the logistic regression models measuring the association between metabolites and MetS: The class of each metabolite was determined by Human Metabolome Database (HMDB) classification, and the primary pathway was determined by the HMDB pathway database tool in addition to a literature search identifying pathways relevant to MetS and related conditions.

Metabolite	Class	Primary Pathway *
Lactate	Alpha hydroxy acids	Gluconeogenesis
2-hydroxybutyrate	Alpha hydroxy acids	Glutathione production
Pro	Carboxylic acids	Amino acid metabolism
Phe	Carboxylic acids	Aromatic amino acid metabolism
Tyr	Carboxylic acids	Aromatic amino acid metabolism
Ile	Carboxylic acids	BCAA metabolism
Leu	Carboxylic acids	BCAA metabolism
Val	Carboxylic acids	BCAA metabolism
Ala	Carboxylic acids	Gluconeogenesis
Glu	Carboxylic acids	Glutathione metabolism
Cystine	Carboxylic acids	Glutathione metabolism
Gly	Carboxylic acids	Glutathione metabolism
Gln	Carboxylic acids	Glutathione metabolism
Alphaaminoapidate	Carboxylic acids	Lysine degradation
Isocitrate	Carboxylic acids	Tricarboxylic acid cycle
Methyl-2-oxopentanoate	Short-chain keto acids	BCAA metabolism
Oxoisopentanoate	Short-chain keto acids	BCAA metabolism
Pyruvate	Unclassified	Gluconeogenesis

BCAA: branched-chain amino acid; * the likely pathway of primary biologic relevance to MetS and related conditions.

## Supplementary Materials

The following are available online at https://www.mdpi.com/1422-0067/21/4/1249/s1, Figure S1: title, Table S1: title, Video S1: title.

## Abbreviations

BLSA	Baltimore Longitudinal Study of Aging
TMCS	Tsuruoka Metabolomics Cohort Study
MetS	Metabolic Syndrome
CE-TOFMS	Capillary electrophoresis time-of-flight mass spectrometry
AD	Alzheimer’s disease
HMDB	Human Metabolome Database
FDR	False-discovery rate
PA	Physical activity
MET	Metabolic equivalent
DASH	Dietary Approaches to Stop Hypertension
LOD	Limit of detection
IQR	Interquartile range
BCAA	Branched-chain amino acid
BCKA	Branched-chain keto acid
GSH	Glutathione
TCA	Tricarboxylic acid
