# Supplementary material for: Blood Metabolite Signature of Metabolic Syndrome Implicates Alterations in Amino Acid Metabolism: Findings from the Baltimore Longitudinal Study of Aging (BLSA) and the Tsuruoka Metabolomics Cohort Study (TMCS)
Source: Int J Mol Sci. 2020 Feb 13;21(4):1249. doi: 10.3390/ijms21041249 (PMC7072861; doi:10.3390/ijms21041249)
Supplement: Supplementary file 1 [file ijms-21-01249-s001.pdf]

Supplementary Tables

Supplementary Table 1. Metabolite classifications from the Human Metabolome Database.

Class and subclass were identified through a search of the HMDB database using the metabolite name or known alternate names for each metabolite.

**Metabolite**	**Class**	**Subclass**
triethanolamine	Amines	Alkanolamines
terephthalate	Benzene and substituted derivatives	Benzoic acids and derivatives
hippurate	Benzene and substituted derivatives	Benzoic acids and derivatives
Aminoisobutyrate	Carboxylic acids and derivatives	Amino acids, peptides, and analogues
thr	Carboxylic acids and derivatives	Amino acids, peptides, and analogues
gly	Carboxylic acids and derivatives	Amino acids, peptides and analogues
betaala	Carboxylic acids and derivatives	Amino acids, peptides, and analogues
ala	Carboxylic acids and derivatives	Amino acids, peptides, and analogues
sarcosine	Carboxylic acids and derivatives	Amino acids, peptides, and analogues
nndimethylglycine	Carboxylic acids and derivatives	Amino acids, peptides, and analogues
ser	Carboxylic acids and derivatives	Amino acids, peptides, and analogues
creatinine	Carboxylic acids and derivatives	Amino acids, peptides, and analogues
pro	Carboxylic acids and derivatives	Amino acids, peptides, and analogues
guanidinoacetate	Carboxylic acids and derivatives	Amino acids, peptides, and analogues
val	Carboxylic acids and derivatives	Amino acids, peptides, and analogues
betaine	Carboxylic acids and derivatives	Amino acids, peptides, and analogues
pipecolate	Carboxylic acids and derivatives	Amino acids, peptides, and analogues
Hydroxyproline	Carboxylic acids and derivatives	Amino acids, peptides, and analogues
creatine	Carboxylic acids and derivatives	Amino acids, peptides, and analogues
ile	Carboxylic acids and derivatives	Amino acids, peptides, and analogues
leu	Carboxylic acids and derivatives	Amino acids, peptides, and analogues
asn	Carboxylic acids and derivatives	Amino acids, peptides, and analogues

ornithine	Carboxylic acids and derivatives	Amino acids, peptides, and analogues
asp	Carboxylic acids and derivatives	Amino acids, peptides, and analogues
prolinebetaine	Carboxylic acids and derivatives	Amino acids, peptides, and analogues
gln	Carboxylic acids and derivatives	Amino acids, peptides, and analogues
lys	Carboxylic acids and derivatives	Amino acids, peptides, and analogues
glu	Carboxylic acids and derivatives	Amino acids, peptides, and analogues
met	Carboxylic acids and derivatives	Amino acids, peptides, and analogues
his	Carboxylic acids and derivatives	Amino acids, peptides, and analogues
alphaaminoadipate	Carboxylic acids and derivatives	Amino acids, peptides, and analogues
phe	Carboxylic acids and derivatives	Amino acids, peptides, and analogues
Methylhistidine	Carboxylic acids and derivatives	Amino acids, peptides, and analogues
arg	Carboxylic acids and derivatives	Amino acids, peptides, and analogues
guanidinosuccinate	Carboxylic acids and derivatives	Amino acids, peptides, and analogues
citrulline	Carboxylic acids and derivatives	Amino acids, peptides, and analogues
tyr	Carboxylic acids and derivatives	Amino acids, peptides, and analogues
sdma	Carboxylic acids and derivatives	Amino acids, peptides, and analogues
adma	Carboxylic acids and derivatives	Amino acids, peptides, and analogues
cystine	Carboxylic acids and derivatives	Amino acids, peptides, and analogues
nacetylaspartate	Carboxylic acids and derivatives	Amino acids, peptides, and analogues
cysteinessulfate	Carboxylic acids and derivatives	Amino acids, peptides, and analogues
fumarate	Carboxylic acids and derivatives	Dicarboxylic acids and derivatives
glutarate	Carboxylic acids and derivatives	Dicarboxylic acids and derivatives
cisaconitate	Carboxylic acids and derivatives	Tricarboxylic acids and derivatives
isocitrate	Carboxylic acids and derivatives	Tricarboxylic acids and derivatives
citrate	Carboxylic acids and derivatives	Tricarboxylic acids and derivatives
Oacetylcarnitine	Fatty acyls	Fatty acid esters
gammabutyrobetaine	Fatty acyls	Fatty acids and conjugates
Hexanoate	Fatty acyls	Fatty acids and conjugates
acetylbutyrate	Fatty acyls	Fatty acids and conjugates
Octanoate	Fatty acyls	Fatty acids and conjugates
pelargonate	Fatty acyls	Fatty acids and conjugates

Decanoate	Fatty acyls	Fatty acids and conjugates
azelate	Fatty acyls	Fatty acids and conjugates
glycerophosphate	Glycerophospholipids	Glycerophosphates
glycerophosphorylcholine	Glycerophospholipids	Glycerophosphocholines
hydroxybutyrate_3	Hydroxy acids and derivatives	Beta hydroxy acids and derivatives
malate	Hydroxy acids and derivatives	Beta hydroxy acids and derivatives
lactate	Hydroxy acids and derivatives	Alpha hydroxy acids and derivatives
hydroxybutyrate_2	Hydroxy acids and derivatives	Alpha hydroxy acids and derivatives
urate	Imidazopyrimidines	Purines and purine derivatives
indole3acetate	Indoles and derivatives	Indolyl carboxylic acids and derivatives
trp	Indoles and derivatives	Indolyl carboxylic acids and derivatives
oxoglutarate	Keto acids and derivatives	Gamma-keto acids and derivatives
Oxoisopentanoate	Keto acids and derivatives	Short-chain keto acids and derivatives
methyl2oxopentanoate	Keto acids and derivatives	Short-chain keto acids and derivatives
taurine	Organic sulfonic acids and derivatives	Organosulfonic acids and derivatives
indoxylsulfate	Organic sulfuric acids and derivatives	Arylsulfates
trimethylaminenoxide	Organonitrogen compounds	Aminoxides
choline	Organonitrogen compounds	Quaternary ammonium salts
carnitine	Organonitrogen compounds	Quaternary ammonium salts
threonate	Organooxygen compounds	Carbohydrate and carbohydrate conjugates
succinate	Organooxygen compounds	Carbohydrates and carbohydrate conjugates
glucuronate	Organooxygen compounds	Carbohydrates and carbohydrate conjugates
Mucate	Organooxygen compounds	Carbohydrates and carbohydrate conjugates
kynurenine	Organooxygen compounds	Carbonyl compounds
Methylnicotinamide	Pyridines and derivatives	Pyridine-carboxylic acids and derivatives

uridine	Pyrimidine nucleosides	Pyrimidine nucleosides
ab	Unclassified	Unclassified
pyruvate	Unclassified	Unclassified
Oxoproline	Unclassified	Unclassified

Supplementary Table 2. Median metabolite comparisons between BLSA and TMCS.

Wilcoxon rank-sum tests were used to compare metabolite concentrations.
BLSA: Baltimore Longitudinal Study of Aging; TMCS: Tsuruoka Metabolomics Cohort Study.

**Metabolite**	**BLSA**			**TMCS**			**FDR-adjusted p-value**
	**Median**	**25% Quartile**	**75% Quartile**	**Median**	**25% Quartile**	**75% Quartile**	
gly	346.6590	297.9980	413.2901	225.1098	192.6657	273.8031	<.0001
trimethylaminenoxide	6.2121	4.2827	10.4510	4.7151	2.7631	8.8180	<.0001
betaala	4.2104	3.4477	5.3380	2.4040	1.8762	3.2546	<.0001
ala	507.1895	434.3979	577.0248	332.3874	285.3466	395.8898	<.0001
sarcosine	1.9066	1.5804	2.3440	2.8626	2.1144	3.7856	<.0001
aminoisobutyrate	1.6654	1.1903	2.3432	1.9216	1.0954	3.0720	0.0719
ab	24.7239	20.8691	29.8938	20.3995	17.1045	24.2344	<.0001
nndimethylglycine	3.7424	3.0813	4.6026	5.0215	3.8782	6.2047	<.0001
choline	31.0605	25.7066	36.9402	15.0024	12.7491	17.6179	<.0001
ser	158.9177	139.5379	176.7704	115.3446	102.9358	129.5237	<.0001
creatinine	69.7186	59.0892	82.2621	58.6956	50.4210	70.2499	<.0001
pro	203.2958	167.3519	240.3784	123.5639	101.9918	149.4326	<.0001
guanidinoacetate	2.4351	2.0284	2.8293	2.5747	2.0849	3.1803	0.0004
val	267.8353	239.2944	292.5737	215.7620	188.5032	247.4084	<.0001
betaine	49.0550	41.3603	56.9190	56.5871	47.2893	67.3843	<.0001
thr	148.4164	132.2355	165.2754	126.2662	109.8556	145.3971	<.0001

taurine	172.3725	147.1813	197.4634	74.3049	64.8132	86.7754	<.0001
pipecolate	1.7758	1.3966	2.9618	1.3216	1.0220	1.8732	<.0001
hydroxyproline	12.4667	9.8897	16.9533	7.9553	6.6185	9.7634	<.0001
creatine	33.0460	22.3931	44.8298	38.4932	27.3322	50.7825	0.0003
ile	66.8646	59.0025	78.0149	53.9487	46.2084	64.0857	<.0001
leu	153.7695	137.0353	170.1310	113.3951	97.0674	129.4709	<.0001
asn	58.4164	53.8317	63.1061	45.4244	40.7959	51.4163	<.0001
ornithine	82.0486	69.1657	94.1837	36.2452	31.3595	42.5361	<.0001
asp	43.1926	38.2682	49.1246	3.6761	2.8510	5.6428	<.0001
methylnicotinamide	0.4726	0.3695	0.6433	0.4998	0.3457	0.6557	0.9747
prolinebetaine	13.2619	3.7118	23.7585	2.1832	1.1707	4.7594	<.0001
gammabutyrobetaine	1.1106	0.9260	1.2822	1.3666	1.1556	1.6462	<.0001
gln	601.5756	553.8508	642.1182	648.1966	596.3095	705.3124	<.0001
lys	218.7084	203.1406	242.9836	176.7810	155.9570	198.9044	<.0001
glu	133.2329	108.8419	159.3250	32.3027	24.1248	42.3827	<.0001
met	24.6544	20.5385	28.9156	18.9167	14.0114	22.7383	<.0001
triethanolamine	0.2419	0.1757	0.3233	1.2411	0.6884	2.0147	<.0001
his	79.0593	74.0377	86.4249	72.8119	66.3083	78.6838	<.0001
alphaaminoadipate	0.9738	0.7493	1.2086	1.0261	0.8226	1.2604	0.0217
carnitine	52.7949	47.2295	60.9552	62.8674	54.5828	70.9440	<.0001
phe	90.6306	82.2757	97.8659	53.9027	48.8927	59.3404	<.0001
methylhistidine	15.7110	10.0726	23.3666	5.0232	3.6528	8.2025	<.0001
arg	101.1714	85.8017	113.2558	85.0822	74.7995	94.5621	<.0001
guanidinosuccinate	0.4321	0.3318	0.5927	0.4698	0.3201	0.6286	0.9263
indole3acetate	2.6360	2.1164	3.2311	4.8906	2.3905	7.5788	<.0001
citrulline	55.5717	47.4846	64.6817	43.5503	37.3095	49.1106	<.0001
tyr	73.4601	65.3192	84.1446	61.1412	54.5940	70.6574	<.0001
sdma	0.5747	0.4861	0.6820	0.5541	0.4502	0.6610	0.0065
adma	0.5533	0.4891	0.6241	0.5395	0.4478	0.6415	0.0635
oacetylcarnitine	0.6754	0.3360	1.1723	0.7855	0.4176	1.2814	0.0189

trp	55.7179	49.8577	61.4163	46.6087	41.2667	52.2516	<.0001
kynurenine	1.8682	1.6059	2.1967	1.5778	1.3355	1.8940	<.0001
cystine	51.1044	41.9651	60.3313	61.5781	55.2546	68.2158	<.0001
uridine	23.6623	21.8393	25.5747	23.8182	19.9807	29.7288	0.2755
glycerophosphorylcholine	12.8277	10.8503	14.9936	5.9383	4.8798	7.4174	<.0001
pyruvate	45.0313	33.9915	55.3932	63.5563	51.4585	77.6452	<.0001
lactate	1736.1398	1447.8995	2032.6032	651.3902	531.1770	802.1482	<.0001
hydroxybutyrate_2	33.0740	26.8653	42.3531	35.5222	28.3317	44.2596	0.0523
hydroxybutyrate_3	56.1272	39.2703	85.8255	84.3078	53.0616	148.7473	<.0001
fumarate	1.8792	1.5442	2.2563	1.7207	1.3698	2.1605	0.0017
oxoisopentanoate	12.2004	10.7138	13.5754	13.4588	11.6178	15.1734	<.0001
hexanoate	5.9453	5.1856	6.7386	8.3493	7.0132	9.8724	<.0001
succinate	11.9939	10.1058	14.0248	9.5699	8.1109	11.2661	<.0001
oxoproline	47.0167	41.0473	54.0453	27.9016	24.1717	32.1348	<.0001
methyl2oxopentanoate	47.2239	40.1845	55.0041	49.8576	41.7089	57.8870	0.0022
acetylbutyrate	1.7842	1.5137	2.0462	1.6535	1.1657	2.0909	0.0049
glutarate	4.3100	2.8687	5.5270	4.5841	3.5591	5.8675	0.0006
malate	7.2647	5.8644	8.6264	6.2071	5.2270	7.3108	<.0001
threonate	32.1082	26.3712	37.8609	30.5966	25.8203	35.5713	0.0243
octanoate	1.6839	1.3643	1.9985	2.4633	2.0958	2.8823	<.0001
oxoglutarate	10.8199	8.3835	14.1896	13.3713	11.6281	15.8200	<.0001
pelargonate	3.3506	2.2807	4.2772	3.3923	2.9236	4.1151	0.0103
terephthalate	1.4947	1.2870	1.7757	0.6860	0.5373	0.8572	<.0001
urate	283.5751	238.7524	334.0695	259.6904	215.9469	305.6050	<.0001
glycerophosphate	4.6515	3.3747	5.8516	2.5489	2.0420	3.0992	<.0001
decanoate	3.2544	2.7880	3.8525	7.3086	6.0607	8.7219	<.0001
cisaconitate	2.5629	2.2462	3.1854	5.7546	4.8667	6.6105	<.0001
nacetylaspartate	0.5536	0.4675	0.6582	0.7407	0.6057	0.8945	<.0001
hippurate	5.7742	2.9516	9.7600	4.1811	1.5200	6.8677	<.0001
azelate	55.3257	1.0007	75.5652	0.9414	0.7811	1.1826	<.0001

isocitrate	4.3289	3.6953	5.2476	5.3206	4.3231	6.6892	<.0001
citrate	81.4959	71.3022	93.0391	105.4264	87.9522	128.1160	<.0001
glucuronate	4.5203	3.9433	5.7970	3.7086	2.9678	4.5770	<.0001
cysteinessulfate	1.6015	1.2300	4.0881	1.0159	0.2399	1.3372	<.0001
mucate	9.6127	6.8518	12.0668	8.1004	6.5964	9.5099	<.0001
indoxylsulfate	4.4620	3.0948	6.2289	3.9497	2.6277	5.6507	0.0017

*Units for metabolite concentrations are μM.

Supplementary Table 3a. Logistic regression model results for BLSA cohort.

MetS: Metabolic syndrome; OR: odds ratio; WC: waist circumference; TG: triglyceride; BP: blood pressure; FG: fasting glucose.

**Metabolite**	**MetS OR**	**MetS p-value**	**MetS FDR p-value**	**WC OR**	**WC p-value**	**WC FDR p-value**	**Elevated TGs OR**	**Elevated TGs p-value**	**Elevated TGs FDR p-value**	**Reduced HDL-c OR**	**Reduced HDL-c p-value**	**Reduced HDL-c FDR p-value**	**BP OR**	**BP p-value**	**BP FDR p-value**	**Elevated FG OR**	**Elevated FG p-value**	**Elevated FG FDR p-value**
urate†	2.86	1.55e-05	0.0012749	3.12	6.8e-06	0.0002781	1.79	0.0121316	0.110532	1.46	0.0835315	0.3678401	1.46	0.1016421	0.5556437	2.57	0.0001303	0.0001303
alphaaminoadipate*	2.48	4.06e-05	0.0016659	2.04	0.0010448	0.0154273	1.8	0.0068657	0.0703738	1.41	0.0836582	0.3678401	1.58	0.0278568	0.2855326	2.46	7.36e-05	7.36e-05
phe*	2.03	0.0002112	0.0043297	1.65	0.0063231	0.0345665	1.82	0.0020797	0.0426343	1.71	0.0034914	0.095431	1.24	0.2353416	0.7132589	1.26	0.1859604	0.1859604
oxoglutarate†	2.19	0.0001775	0.0043297	1.9	0.0014604	0.0171107	2.02	0.0011848	0.0323834	1.83	0.0023635	0.095431	1.26	0.2377419	0.7132589	1.68	0.0098953	0.0098953
glu*	2.24	0.0003067	0.004775	2.3	0.000298	0.0081459	1.63	0.0248187	0.1356754	1.98	0.0014406	0.095431	1.18	0.4472154	0.8929173	1.62	0.024978	0.024978
glucuronate†	2.02	0.0003494	0.004775	1.45	0.0483555	0.158606	1.76	0.0042913	0.0502699	1.34	0.1064271	0.3678401	1.48	0.0434141	0.3955502	1.65	0.0096971	0.0096971
hydroxyb	1.96	0.0004903	0.0057433	1.92	0.0008692	0.0154273	1.45	0.0507587	0.209999	1.45	0.0419114	0.2863942	1.6	0.0147153	0.2855326	2.18	0.0001256	0.0001256

utyrate_2*																		
cystine*	2.11	0.0008149	0.0083527	2.15	0.0011288	0.0154273	2.12	0.0011757	0.0323834	1.76	0.0063302	0.1038153	1.64	0.0219236	0.2855326	1.15	0.4951075	0.4951075
asn†	0.56	0.0016465	0.0135009	0.57	0.0020046	0.0182641	0.71	0.0599085	0.2339284	0.76	0.1164713	0.3678401	0.77	0.1411488	0.6935139	0.94	0.7149429	0.7149429
methyl2oxopentanoate*	2.05	0.0015337	0.0135009	2.01	0.0024752	0.0184518	1.75	0.0152324	0.1135504	1.57	0.038201	0.2847711	1.48	0.0820901	0.5177988	2.5	0.0001543	0.0001543
ile*	1.96	0.0031686	0.0216553	1.99	0.0033765	0.0230725	1.38	0.1699463	0.4738501	1.5	0.0665351	0.3678401	0.98	0.9471096	0.9830758	1.92	0.0049199	0.0049199
leu*	1.93	0.0031691	0.0216553	1.81	0.0086406	0.0442829	1.55	0.0512193	0.209999	1.77	0.0088087	0.1203858	1.32	0.2105313	0.7132589	1.64	0.0240218	0.0240218
oxoisopentanoate*	1.76	0.0036462	0.022999	1.83	0.0024743	0.0184518	1.64	0.0136646	0.1120496	1.31	0.1463558	0.4000392	1.67	0.0094109	0.2855326	1.82	0.0026977	0.0026977
gln*	0.55	0.0043607	0.0255414	0.63	0.0278492	0.1085488	0.76	0.1867839	0.4738501	0.84	0.38161	0.6519171	0.69	0.0672048	0.4934755	0.8	0.2865875	0.2865875
guanidinoacetate†	0.54	0.0068109	0.0372327	0.82	0.3833299	0.6548552	0.62	0.0352568	0.1700623	0.73	0.1419583	0.4000392	0.72	0.1516246	0.6935139	1.15	0.5198416	0.5198416
carnitine†	1.66	0.0081634	0.0406283	1.25	0.2440806	0.5559615	1.56	0.0219207	0.1356754	1.29	0.1632407	0.4317979	1.63	0.0146088	0.2855326	1.62	0.0144279	0.0144279
tyr*	1.65	0.0084229	0.0406283	1.85	0.0018745	0.0182641	1.76	0.004079	0.0502699	1.36	0.0968901	0.3678401	1.28	0.1962907	0.7132589	1.41	0.0712558	0.0712558
gly*	0.57	0.0109962	0.0500938	0.64	0.0424553	0.1450555	0.6	0.0247937	0.1356754	0.93	0.7252369	0.8583158	0.61	0.0275791	0.2855326	1.03	0.9075403	0.9075403
uridine†	1.62	0.0159485	0.0653887	1.45	0.0680407	0.2066422	1.37	0.1281642	0.3892394	1.36	0.1166322	0.3678401	1.65	0.0154208	0.2855326	1.83	0.0036466	0.0036466
pro*	1.62	0.0155648	0.0653887	1.26	0.2546814	0.564429	2	0.0011668	0.0323834	1.63	0.013614	0.1594784	1.08	0.7023314	0.958181	1.47	0.0553605	0.0553605
ala*	1.59	0.0215372	0.0840977	1.84	0.0044653	0.0281657	1.31	0.1850624	0.4738501	1.27	0.2107447	0.4670559	1.05	0.7949921	0.958181	1.71	0.0105376	0.0105376
pyruvate*	1.48	0.022814	0.085034	1.65	0.0056176	0.0329032	1.48	0.0293109	0.1502183	1.24	0.1971317	0.4670559	1.1	0.5893669	0.9529254	1.32	0.1124355	0.1124355
isocitrate*	1.55	0.0272106	0.0970115	1.27	0.2310166	0.5508608	1.22	0.3266301	0.5666233	0.97	0.8887592	0.9330836	1.59	0.0240846	0.2855326	1.06	0.7579231	0.7579231
lactate*	1.61	0.0288757	0.0986586	3.16	6.6e-06	0.0002781	1.24	0.3273595	0.5666233	1.6	0.0290942	0.2847711	1.17	0.4671827	0.8929173	1.79	0.0109241	0.0109241
val*	1.51	0.0384247	0.1250204	1.57	0.0291228	0.1085488	1.45	0.0731848	0.2723044	1.38	0.0995825	0.3678401	1.19	0.397656	0.8929173	1.66	0.0140339	0.0140339

asp	1.44	0.0396406	0.1250204	1.41	0.0559126	0.1763398	1.18	0.3489762	0.5666233	1.45	0.0360764	0.2847711	0.97	0.885247	0.9690342	1.01	0.9401103	0.9401103
taurine	0.7	0.0487181	0.1479588	0.87	0.4479851	0.694422	0.85	0.3817128	0.5905745	1.17	0.3804769	0.6519171	0.87	0.4693061	0.8929173	0.74	0.1002705	0.1002705
kynurenine	1.42	0.059985	0.1756703	1.5	0.036755	0.1310396	1.4	0.0836536	0.2858166	1.32	0.1263946	0.3701555	1.42	0.0722159	0.4934755	0.97	0.8737608	0.8737608
betaala	1.45	0.066824	0.1889505	1.23	0.3170903	0.6459306	1.24	0.2995782	0.5666233	1.25	0.270682	0.5284743	1.2	0.3910166	0.8929173	1.37	0.1234898	0.1234898
creatinine	1.54	0.0989546	0.2704758	1.01	0.9682452	0.9801988	1.35	0.2736941	0.5473882	0.96	0.8657615	0.9330836	1.36	0.2516465	0.7132589	1.05	0.8408483	0.8408483
methylhistidine	1.36	0.1035021	0.2737797	0.96	0.8247282	0.9084175	1.48	0.0468146	0.209999	1.2	0.3292477	0.5869198	1.27	0.2237699	0.7132589	1.28	0.2009708	0.2009708
glycerophosphate	0.66	0.1078394	0.2763386	0.55	0.0240341	0.103726	0.65	0.1117298	0.3523787	1.04	0.8640148	0.9330836	1	0.9945072	0.9945072	1.04	0.8914283	0.8914283
hydroxyproline	1.35	0.1172216	0.2912779	1.29	0.1873698	0.4801352	1.1	0.6195232	0.8024185	0.85	0.3950907	0.6611722	1.45	0.0597757	0.4901607	1.36	0.1280557	0.1280557
oxoproline	0.73	0.1226328	0.2957615	0.89	0.5824415	0.7654511	0.61	0.02331	0.1356754	0.78	0.2195247	0.4737113	1.04	0.8589644	0.9690342	1.29	0.2312121	0.2312121
azelate	4.26	0.1774736	0.4157953	0.99	0.9926722	0.9926722	6.42	0.0996082	0.3267151	2.32	0.4378325	0.6648568	1.8	0.5926731	0.9529254	10.02	0.0383527	0.0383527
indoxylsulfate	1.26	0.2148111	0.4892918	1.21	0.3019977	0.6459306	1.27	0.2237079	0.5091814	1.1	0.5901207	0.7763451	1.07	0.7264501	0.958181	1.21	0.3242103	0.3242103
nndimethylglycine	1.25	0.2362706	0.4967741	1.32	0.1553875	0.4306571	1.28	0.2100594	0.5066139	1.38	0.0956774	0.3678401	1.08	0.7140729	0.958181	1.33	0.1536487	0.1536487
hydroxybutyrate_3	1.25	0.2361668	0.4967741	1.07	0.7388596	0.8513989	1.29	0.1906958	0.4738501	1.16	0.436311	0.6648568	1.06	0.7551738	0.958181	1.36	0.1169592	0.1169592
nacetylaspartate	0.8	0.2318173	0.4967741	0.88	0.4943413	0.723857	0.56	0.0041343	0.0502699	0.6	0.0058294	0.1038153	1.06	0.7476071	0.958181	0.98	0.9289166	0.9289166

creatine	1.29	0.2436225	0.4994261	1.17	0.4664378	0.7082945	1.11	0.6267624	0.8024185	1.19	0.4052188	0.6645589	1.37	0.1580975	0.6935139	2.01	0.0030579	0.0030579
mucate	0.84	0.3490046	0.6980092	0.89	0.5440174	0.7633417	1	0.989196	0.989196	0.98	0.9103255	0.9330836	1.24	0.2784016	0.7609644	0.77	0.1898986	0.1898986
trp	1.17	0.4245057	0.7911243	1.1	0.6249143	0.7764087	1.21	0.3447414	0.5666233	1.39	0.0934689	0.3678401	1.16	0.4716655	0.8929173	1.34	0.1467392	0.1467392
glutarate	0.85	0.4094869	0.7911243	1.2	0.3465969	0.6459306	0.92	0.6822048	0.8349372	0.93	0.7018101	0.8463004	1.16	0.4610684	0.8929173	0.85	0.4096753	0.4096753
malate	1.19	0.4160483	0.7911243	1.66	0.025703	0.1053825	0.78	0.2632299	0.5396213	1.1	0.6434038	0.811267	1.15	0.533362	0.9305464	1.71	0.0186557	0.0186557
lys	0.86	0.4393884	0.8006633	0.76	0.1575575	0.4306571	1.02	0.9082876	0.9500554	1.05	0.8154076	0.9159373	1.05	0.814966	0.958181	1.12	0.5581157	0.5581157
arg	1.14	0.4782816	0.8525889	1.32	0.1642071	0.4343544	0.91	0.6231361	0.8024185	0.79	0.2103075	0.4670559	1.06	0.7634363	0.958181	1.72	0.0079272	0.0079272
succinate	1.16	0.4903142	0.8554419	1.73	0.0152019	0.0733268	0.99	0.9545284	0.9783916	0.92	0.68518	0.8385786	1.36	0.1716971	0.6935139	1.33	0.1984308	0.1984308
ab	1.12	0.5571995	0.8767424	1.12	0.5512701	0.7633417	1.16	0.4397447	0.6677605	1.27	0.2028021	0.4670559	1.12	0.5468875	0.9342662	1.76	0.005708	0.005708
betaine	1.11	0.5682087	0.8767424	0.84	0.3390809	0.6459306	1.26	0.2253792	0.5091814	1.23	0.2565532	0.5173559	1.04	0.8296445	0.958181	0.97	0.8911876	0.8911876
thr	0.91	0.5901895	0.8767424	0.96	0.8308697	0.9084175	0.98	0.9126284	0.9500554	0.91	0.5780688	0.7763451	1.2	0.2987126	0.790143	1.11	0.5342217	0.5342217
ornithine	1.1	0.6302656	0.8767424	1.19	0.3738122	0.6548552	0.97	0.8966096	0.9500554	1.5	0.0324277	0.2847711	0.99	0.9373607	0.9830758	1.16	0.4346935	0.4346935
methylnicotinamide	1.09	0.6477893	0.8767424	0.83	0.3345405	0.6459306	1.4	0.0763781	0.2723044	1	0.9799502	0.9906333	1.05	0.808185	0.958181	0.75	0.1306787	0.1306787
prolinebetaine	0.92	0.6522108	0.8767424	1.16	0.4759311	0.70957	0.79	0.2359621	0.5091814	0.95	0.8008471	0.9120759	0.91	0.6540075	0.958181	0.87	0.4885932	0.4885932
indole3acetate	1.09	0.6390022	0.8767424	1.62	0.0165376	0.075338	1.23	0.3073066	0.5666233	0.91	0.6059279	0.7763451	0.99	0.9649766	0.9891011	1.18	0.399878	0.399878
adma	0.9	0.5835007	0.8767424	1.28	0.2031784	0.5048675	1.04	0.8437136	0.9500554	1.12	0.5471842	0.7703047	1	0.9888073	0.9945072	0.85	0.4112826	0.4112826
fumarate	1.1	0.625998	0.8767424	1.37	0.1346424	0.39431	0.96	0.8298361	0.9500554	1.21	0.3270308	0.5869198	1.05	0.8285653	0.958181	1.19	0.3912086	0.3912086
acetylbutyrate	1.13	0.5286296	0.8767424	1.02	0.9051377	0.9277662	1.15	0.4729548	0.692541	1.03	0.8685751	0.9330836	1.09	0.6719824	0.958181	1.14	0.4933298	0.4933298
decanoate	1.1	0.6085615	0.8767424	0.88	0.507233	0.7297036	1.1	0.6251906	0.8024185	0.86	0.4146538	0.6648568	1.13	0.5328012	0.9305464	1.21	0.3406765	0.3406765

cisaconitate	1.11	0.5773316	0.8767424	1.1	0.6068133	0.7655183	0.95	0.7723946	0.9179182	0.8	0.2036275	0.4670559	1.29	0.1757319	0.6935139	1.11	0.5635985	0.5635985
hippurate	0.93	0.6521778	0.8767424	1.02	0.8993822	0.9277662	0.98	0.9152973	0.9500554	0.84	0.3011394	0.5672766	0.96	0.8124084	0.958181	1	0.998444	0.998444
cysteinessulfate	1.18	0.6321721	0.8767424	1.13	0.7112498	0.8331784	0.82	0.553971	0.7832003	1.66	0.1242991	0.3701555	0.85	0.615611	0.958181	1.15	0.7008306	0.7008306
triethanolamine	1.1	0.670497	0.8867864	1.04	0.8492549	0.9163014	1.25	0.3159778	0.5666233	1.42	0.1050324	0.3678401	0.76	0.2478779	0.7132589	1.22	0.3590953	0.3590953
sarcosine	0.94	0.7368695	0.9018403	0.85	0.3787975	0.6548552	1.24	0.2338017	0.5091814	1.09	0.6018628	0.7763451	1.05	0.7881221	0.958181	1.18	0.3613233	0.3613233
gammabutyrobetaine	0.93	0.7274059	0.9018403	0.79	0.2351235	0.5508608	0.97	0.8752962	0.9500554	0.82	0.3043923	0.5672766	0.87	0.4961141	0.9040302	1.04	0.8516508	0.8516508
his	0.93	0.7028794	0.9018403	0.91	0.5880905	0.7654511	1.31	0.1440646	0.4219034	1.12	0.5310666	0.7703047	1.19	0.3414171	0.8308331	1.17	0.3814172	0.3814172
citrulline	0.93	0.7215774	0.9018403	0.84	0.4298674	0.6911593	1.12	0.5937074	0.8024185	0.93	0.7327086	0.8583158	0.78	0.2522501	0.7132589	1.23	0.3472109	0.3472109
oacetylcarnitine	1.07	0.7280686	0.9018403	0.88	0.5668399	0.7633417	1.15	0.5061536	0.7281508	1.03	0.8795215	0.9330836	0.97	0.8825241	0.9690342	1.29	0.2418903	0.2418903
sdma	0.93	0.7536104	0.9087654	0.81	0.333843	0.6459306	1.23	0.3593221	0.5666233	1.13	0.5542436	0.7703047	0.85	0.4611296	0.8929173	0.89	0.6115819	0.6115819
ser	1.05	0.8046092	0.929267	0.91	0.6720198	0.8069807	0.91	0.676086	0.8349372	1.45	0.0829508	0.3678401	0.74	0.1776072	0.6935139	1.16	0.4993865	0.4993865
pipecolate	1.05	0.7884579	0.929267	0.87	0.4488337	0.694422	1.19	0.3586824	0.5666233	1	0.9906333	0.9906333	1.21	0.3223697	0.8260723	1.04	0.832573	0.832573
threonate	0.95	0.7961146	0.929267	0.97	0.8807766	0.9277662	1.27	0.2511444	0.5280471	1.4	0.0981808	0.3678401	0.92	0.6792021	0.958181	1.01	0.9434764	0.9434764
guanidinosuccinate	1.04	0.8312077	0.9466532	0.92	0.6790448	0.8069807	1.01	0.9786958	0.989196	0.85	0.4240695	0.6648568	0.93	0.7252214	0.958181	0.92	0.676561	0.676561
pelargonate	0.95	0.8689577	0.9760894	1.28	0.4288221	0.6911593	1.27	0.4521972	0.6741849	0.85	0.5831674	0.7763451	0.74	0.3444918	0.8308331	1.59	0.1335259	0.1335259
trimethyla	0.98	0.9193957	0.9852918	1.11	0.5678517	0.7633417	0.92	0.6360634	0.8024185	0.88	0.4522394	0.6742479	0.98	0.9235115	0.9830758	1.04	0.8460522	0.8460522

minenoxide																		
aminoisobutyrate	1.02	0.9143648	0.9852918	0.97	0.8917433	0.9277662	1.11	0.6138026	0.8024185	1.26	0.2558316	0.5173559	1.16	0.4619424	0.8929173	0.88	0.5487635	0.5487635
met	0.97	0.9031672	0.9852918	0.89	0.6054908	0.7655183	0.97	0.894332	0.9500554	0.91	0.652971	0.811267	1.07	0.761834	0.958181	1.24	0.3356561	0.3356561
glycerophosphorylcholine	1.01	0.9633757	0.9852918	0.95	0.8154153	0.9084175	0.96	0.8492481	0.9500554	0.89	0.5519454	0.7703047	0.97	0.8863118	0.9690342	1.2	0.3623529	0.3623529
hexanoate	0.99	0.9410996	0.9852918	0.94	0.7475698	0.8513989	0.78	0.1904931	0.4738501	0.79	0.2031546	0.4670559	1.37	0.0974794	0.5556437	1.2	0.3254788	0.3254788
octanoate	0.99	0.9596511	0.9852918	0.84	0.3724342	0.6548552	0.97	0.8700139	0.9500554	0.8	0.258678	0.5173559	0.87	0.4791264	0.8929173	1.33	0.1479176	0.1479176
terephthalate	1.01	0.973276	0.9852918	0.92	0.6455011	0.7900163	0.84	0.3550024	0.5666233	1.06	0.7467034	0.8623899	1.01	0.9382015	0.9830758	1.15	0.4428181	0.4428181
citrate	1.01	0.9528537	0.9852918	0.83	0.3230478	0.6459306	0.93	0.7088063	0.854737	0.75	0.1149588	0.3678401	1.11	0.5920816	0.9529254	0.84	0.3611277	0.3611277
choline	1	0.991028	0.991028	1.28	0.4089838	0.6844219	0.75	0.3444388	0.5666233	1.04	0.9035426	0.9330836	1.15	0.6541611	0.958181	1.24	0.4652082	0.4652082

* Indicates a metabolite among the 25 most significant metabolites in both BLSA and TMCS.
† Indicates a metabolite which is among the 25 most significant metabolites in the BLSA cohort only.

Supplementary Table 3b. Logistic regression model results for TMCS cohort.

MetS: Metabolic syndrome; OR: odds ratio; WC: waist circumference; TG: triglyceride; BP: blood pressure; FG: fasting glucose.

**Metabolite**	**MetS OR**	**MetS p-value**	**MetS FDR p-value**	**WC OR**	**WC p-value**	**WC FDR p-value**	**Elevated TGs OR**	**Elevated TGs p-value**	**Elevated TGs FDR p-value**	**Reduced HDL-c OR**	**Reduced HDL-c p-value**	**Reduced HDL-c FDR p-value**	**BP OR**	**BP p-value**	**BP FDR p-value**	**Elevated FG OR**	**Elevated FG p-value**	**Elevated FG FDR p-value**

glu*	2.62	<1e-07	<1e-07	2.36	<1e-07	3e-07	2.16	<1e-07	2e-07	1.89	1.6e-06	0.0001283	1.12	0.3523812	0.610458	1.57	0.0001596	0.0001596
pro*	2.22	<1e-07	2e-07	1.79	6.32e-05	0.0007683	1.61	0.0001991	0.0040822	1.57	0.000723	0.0197634	1.34	0.0265016	0.1552239	1.94	2e-07	2e-07
methyl2oxopentanoate*	2.18	<1e-07	4e-07	1.46	0.0101364	0.0433872	1.76	2.07e-05	0.0008476	1.51	0.0026004	0.0388544	1.48	0.0043193	0.0698039	1.83	3.2e-06	3.2e-06
ile*	2.15	1e-07	2.7e-06	1.89	7.5e-05	0.0007683	1.63	0.0004407	0.0060229	1.62	0.0010778	0.0220948	1.14	0.3469516	0.610458	1.52	0.0016959	0.0016959
mucate†	0.53	2e-07	2.7e-06	0.54	8.1e-06	0.0002223	0.69	0.0016342	0.0121819	0.73	0.008216	0.0842138	0.61	9.65e-05	0.007535	0.72	0.0035501	0.0035501
leu*	2.07	7e-07	9.2e-06	2.08	6.7e-06	0.0002223	1.62	0.0007301	0.0073111	1.56	0.002843	0.0388544	1.18	0.2569146	0.5543947	1.66	0.0002218	0.0002218
cystine*	1.84	1.1e-06	1.23e-05	1.37	0.0194199	0.0663514	1.5	0.000857	0.0073111	1.77	1.39e-05	0.0005712	1.37	0.0110338	0.1130964	1.43	0.0022987	0.0022987
alphaaminoadipate*	1.89	1.2e-06	1.28e-05	1.69	0.0002892	0.0023712	1.44	0.0032412	0.0166114	1.36	0.0183331	0.1503312	1.24	0.0904601	0.3371693	1.87	6e-07	6e-07
oxoisopentanoate*	1.74	2.7e-06	2.47e-05	1.43	0.0049633	0.0254368	1.43	0.0020456	0.0133466	1.24	0.0748419	0.2360398	1.19	0.1356267	0.4277458	1.68	5.1e-06	5.1e-06
ala*	1.78	1.04e-05	8.27e-05	1.81	6.67e-05	0.0007683	1.53	0.0008916	0.0073111	1.29	0.0594141	0.2079275	1.17	0.2397012	0.5312296	2.1	0	0
ser†	0.6	1.15e-05	8.27e-05	0.62	0.0001547	0.0014096	0.68	0.0006696	0.0073111	0.92	0.4525585	0.6510491	0.72	0.0051076	0.0698039	0.93	0.5288011	0.5288011
val*	1.8	1.21e-05	8.27e-05	1.86	4.26e-05	0.0006993	1.41	0.0084696	0.0365529	1.29	0.0626118	0.2079275	1.15	0.2946837	0.5893675	1.94	4e-07	4e-07
gly*	0.6	1.91e-05	0.0001206	0.83	0.1525352	0.3474413	0.66	0.0003288	0.0053929	0.79	0.0502224	0.2059116	0.87	0.2240926	0.5312296	0.81	0.0621333	0.0621333
pyruvate*	1.65	4.21e-05	0.0002467	1.18	0.2039538	0.3981954	1.58	0.000156	0.0040822	1.31	0.0309918	0.1588327	1.16	0.2380181	0.5312296	1.37	0.0068198	0.0068198
phe*	1.65	6.54e-05	0.0003565	1.57	0.0013954	0.0081732	1.26	0.0592481	0.147997	1.29	0.0487467	0.2059116	1.17	0.2107374	0.5312296	1.48	0.0011762	0.0011762
tyr*	1.65	6.96e-05	0.0003565	1.82	3.11e-05	0.0006379	1.2	0.132013	0.2517457	1.13	0.3252522	0.5674613	1.08	0.527372	0.7862636	1.63	6.71e-05	6.71e-05
trp†	1.57	0.0005496	0.0026511	1.38	0.0280827	0.0822421	1.39	0.010788	0.0422899	1.22	0.1387539	0.3670264	0.85	0.2369036	0.5312296	1.24	0.0843374	0.0843374
gln*	0.67	0.0007675	0.0034965	0.72	0.0121765	0.0475464	0.7	0.0023343	0.0133466	0.89	0.3396313	0.5683625	0.63	0.0001838	0.007535	1.13	0.269	0.269
hydroxyb	0.66	0.001037	0.0044755	0.62	0.0008483	0.0054861	0.68	0.0024415	0.0133466	0.77	0.0463788	0.2059116	1.13	0.3392838	0.610458	0.89	0.3506441	0.3506441

utyrate_3†																		
guanidinosuccinate†	0.67	0.0014886	0.0061034	0.67	0.0042088	0.0230082	0.77	0.0372914	0.1054445	0.69	0.0044556	0.0521944	0.92	0.4819131	0.7456014	0.75	0.0174133	0.0174133
isocitrate*	1.42	0.0015816	0.0061759	1.27	0.058854	0.1664149	1.32	0.0108303	0.0422899	1.33	0.01407	0.1281937	1.25	0.0492889	0.2245385	1.2	0.0830357	0.0830357
hydroxybutyrate_2*	1.42	0.0016615	0.0061929	1.05	0.6627484	0.7978941	1.29	0.0209631	0.0747379	1.2	0.1129454	0.3193628	1.21	0.0992081	0.3536984	1.49	0.0002462	0.0002462
cisaconitate†	1.38	0.0034581	0.0123289	1.23	0.0961581	0.2464052	1.2	0.0937353	0.1970846	1.29	0.0269692	0.1588327	1.35	0.0100384	0.1130964	1.26	0.0276847	0.0276847
lactate*	1.38	0.0038706	0.0129578	1.25	0.072924	0.1993257	1.25	0.0414458	0.1132852	1.08	0.5055387	0.7026131	1.27	0.0417887	0.2119818	1.48	0.0003441	0.0003441
cysteinessulfate†	1.74	0.0039506	0.0129578	0.9	0.6218341	0.7725818	1.22	0.2964591	0.4191318	1.36	0.1305168	0.3567459	1.32	0.1557546	0.4561384	1.59	0.0111618	0.0111618
carnitine	1.41	0.0048575	0.0153199	1.13	0.3781655	0.5742513	1.3	0.0316049	0.1036639	1.17	0.2243061	0.4840289	1.33	0.0253736	0.1552239	1.31	0.0213755	0.0213755
urate	1.41	0.0082618	0.0250913	1.38	0.0254302	0.0772324	1.12	0.361632	0.4706956	1.05	0.7152676	0.8146103	1.55	0.0012661	0.0259559	1.23	0.0910825	0.0910825
prolinebetaine	0.73	0.0085994	0.025184	0.99	0.9423483	0.9678252	0.77	0.0279537	0.0955084	0.86	0.2164228	0.4840289	0.97	0.7924101	0.8952902	0.87	0.2056081	0.2056081
methylnicotinamide	1.36	0.0100789	0.0284989	1.24	0.1186254	0.2947661	1.38	0.0070373	0.0320587	1.32	0.030222	0.1588327	0.89	0.3714452	0.6216022	1.19	0.1374414	0.1374414
citrate	0.74	0.0105144	0.0287393	0.88	0.3316609	0.5230037	0.81	0.0653865	0.1576969	0.86	0.213209	0.4840289	0.99	0.9211423	0.9441708	0.98	0.8502059	0.8502059
acetylbutyrate	0.74	0.0129538	0.0342648	0.85	0.2349845	0.428194	0.87	0.2467172	0.3817134	0.9	0.4003659	0.6258765	0.65	0.0010959	0.0259559	0.86	0.2126202	0.2126202
decanoate	0.78	0.0138744	0.0355531	1.04	0.753874	0.8706714	0.81	0.034907	0.1054445	0.9	0.3097724	0.5644741	0.95	0.6298989	0.8330921	0.91	0.3370715	0.3370715
gammabutyro	0.75	0.0169674	0.0421615	0.83	0.1766324	0.3715661	0.79	0.0567951	0.147997	0.91	0.4402491	0.6446505	1.09	0.4766899	0.7456014	0.96	0.7365369	0.7365369

betaine																		
met	1.27	0.0274465	0.0661945	1.19	0.1587971	0.3519287	1.21	0.0694759	0.1595625	1.29	0.0303441	0.1588327	1.2	0.0871191	0.3371693	1.06	0.5353396	0.5353396
choline	1.3	0.0340543	0.0775681	1.23	0.1321154	0.3186312	1.13	0.3188734	0.4357936	0.95	0.712924	0.8146103	1.34	0.0235181	0.1552239	1.14	0.2816198	0.2816198
asn	0.76	0.0338922	0.0775681	0.6	0.0004094	0.0030521	0.76	0.0353354	0.1054445	0.9	0.4378067	0.6446505	0.73	0.0218201	0.1552239	1.11	0.3981662	0.3981662
betaine	0.79	0.0372135	0.0824731	0.66	0.0008697	0.0054861	0.87	0.2259034	0.3632172	0.94	0.6155513	0.7765416	1.02	0.8489268	0.8952902	0.84	0.102113	0.102113
threonate	0.78	0.038376	0.0828114	0.69	0.0060225	0.0290496	0.83	0.1155051	0.2310102	0.88	0.3003408	0.559726	0.78	0.0439474	0.2119818	0.87	0.2300136	0.2300136
aminoisobutyrate	0.78	0.0410745	0.0863618	0.78	0.0882463	0.2334256	0.7	0.004075	0.0196557	0.78	0.0534971	0.2064748	1.06	0.662655	0.8530708	0.97	0.8242875	0.8242875
adma	0.79	0.0517317	0.10605	1.09	0.5239291	0.6874337	0.69	0.0021507	0.0133466	0.79	0.0633925	0.2079275	1.09	0.5006332	0.7602207	0.86	0.1852417	0.1852417
creatine	1.27	0.0554638	0.1109275	1.39	0.0214409	0.0703263	1.3	0.0371503	0.1054445	1.15	0.2806597	0.5479547	1.14	0.3233947	0.610458	1.16	0.2205363	0.2205363
glycerophosphate	1.23	0.0575035	0.1122688	1.08	0.5261903	0.6874337	1.31	0.0135377	0.0504586	1.25	0.0553957	0.2064748	1.01	0.9505267	0.9505267	0.84	0.1035899	0.1035899
glucuronate	1.24	0.0633615	0.120829	1.21	0.1436511	0.3365541	1.2	0.1125321	0.2306907	1.29	0.0371629	0.1792563	1.17	0.1962009	0.518983	1.12	0.2939067	0.2939067
thr	1.25	0.0678853	0.1265135	1.1	0.4759533	0.6874337	1.19	0.1494495	0.2785196	1.33	0.0290924	0.1588327	1.08	0.5451125	0.7914604	1.26	0.0457838	0.0457838
succinate	0.81	0.0739253	0.1347083	0.71	0.0074314	0.0338543	0.82	0.0850456	0.1872036	0.86	0.2047318	0.4840289	0.98	0.8849366	0.9185418	1.01	0.9542538	0.9542538
glycerophosphorylcholine	1.21	0.1009621	0.1799758	0.88	0.338883	0.5243095	1.16	0.1995742	0.3339813	1.08	0.4997732	0.7026131	1.24	0.0734745	0.3131439	0.96	0.6774421	0.6774421
hippurate	0.87	0.1418421	0.2474691	0.98	0.8156761	0.9038573	0.84	0.0595598	0.147997	0.89	0.2187963	0.4840289	0.96	0.6762147	0.8530708	1.06	0.4959014	0.4959014
uridine	0.86	0.1744223	0.2979715	1.08	0.5305258	0.6874337	0.9	0.3315009	0.4384367	1.02	0.8737165	0.9068956	0.88	0.2855774	0.5866809	0.92	0.4432149	0.4432149
pipecolate	1.17	0.1908159	0.3193246	0.97	0.8064285	0.9038573	0.93	0.5546611	0.6714583	0.89	0.3607126	0.5799693	1.08	0.5501615	0.7914604	1.36	0.0098608	0.0098608

trimethylaminenoxide	0.87	0.2199177	0.3606651	0.92	0.4977627	0.6874337	0.85	0.1613197	0.2875699	0.85	0.1784351	0.45724	0.93	0.567839	0.8028069	1.07	0.5435934	0.5435934
terephthalate	0.88	0.2627462	0.4224547	1.06	0.667182	0.7978941	0.92	0.464305	0.5889607	1.01	0.9636014	0.9754977	0.83	0.1280571	0.4277458	0.91	0.3761491	0.3761491
hydroxyproline	1.13	0.3010839	0.4747862	0.99	0.9362399	0.9678252	1.02	0.8945186	0.9284877	0.97	0.7775163	0.8500845	1.01	0.939515	0.9505267	0.99	0.9043927	0.9043927
taurine	1.12	0.3129439	0.4841774	1.39	0.0105822	0.0433872	1.13	0.2963122	0.4191318	1.32	0.0215621	0.1588327	1.14	0.2861858	0.5866809	0.96	0.7423042	0.7423042
sdma	0.89	0.3265441	0.4958633	0.99	0.9116036	0.9678252	0.82	0.0867529	0.1872036	0.81	0.0861805	0.2617335	1.04	0.7240414	0.8952902	0.95	0.6670224	0.6670224
ornithine	1.12	0.3448519	0.4972545	1.02	0.902117	0.9678252	0.96	0.6991464	0.8011196	1.02	0.8667852	0.9068956	0.94	0.5933707	0.8246847	1.08	0.4882293	0.4882293
his	1.12	0.3456525	0.4972545	1.19	0.2035541	0.3981954	1.14	0.2697272	0.4021387	1.05	0.6808693	0.7975897	0.97	0.8233662	0.8952902	1.08	0.5260447	0.5260447
oacetylcarnitine	1.12	0.3381297	0.4972545	1.08	0.5396197	0.6874337	1.18	0.1557241	0.283764	0.94	0.6259029	0.7776369	1.19	0.146949	0.4462895	1.17	0.1669288	0.1669288
triethanolamine	0.92	0.3601881	0.504029	1.1	0.3065319	0.5027124	1.01	0.8772798	0.9284877	1	0.9854451	0.9854451	0.97	0.7512542	0.8952902	0.88	0.1216378	0.1216378
oxoproline	0.9	0.362655	0.504029	0.87	0.2736673	0.4675149	0.85	0.1785891	0.311581	1.08	0.5285346	0.7210163	1.03	0.7941699	0.8952902	1.1	0.3869229	0.3869229
creatinine	0.88	0.3897822	0.5239695	1	0.9834402	0.9955814	0.79	0.1275268	0.2489808	0.88	0.435452	0.6446505	1.22	0.2199284	0.5312296	0.94	0.6796011	0.6796011
asp	1.1	0.3869625	0.5239695	1.17	0.1952792	0.3981954	1.11	0.3290012	0.4384367	1.03	0.8291434	0.8829839	0.94	0.6215293	0.8330921	1.03	0.8096231	0.8096231
indole3acetate	0.91	0.4289396	0.5614219	0.95	0.6713987	0.7978941	0.89	0.2886757	0.4191318	0.87	0.2451881	0.5026356	0.96	0.7420324	0.8952902	0.98	0.8880847	0.8880847
octanoate	0.92	0.4368124	0.5614219	0.85	0.2124193	0.4050787	0.99	0.9183084	0.9412661	1.12	0.3506689	0.5750969	1.17	0.1789133	0.5058926	0.87	0.2166576	0.2166576
oxoglutarate	1.09	0.4381829	0.5614219	0.87	0.2427369	0.432456	1.13	0.2638582	0.4006735	1.14	0.2435027	0.5026356	1.1	0.4260494	0.6850206	1.01	0.9386277	0.9386277

kynurenine	1.1	0.4542953	0.5728306	1.41	0.0149833	0.0558469	1.02	0.892805	0.9284877	0.99	0.9138946	0.936742	1.04	0.7820113	0.8952902	0.85	0.164058	0.164058
hexanoate	0.92	0.4610588	0.5728306	1.08	0.5409237	0.6874337	0.86	0.1857352	0.3172976	0.93	0.5658791	0.7250326	0.98	0.8503212	0.8952902	1	0.9798578	0.9798578
fumarate	0.93	0.5338159	0.653327	0.73	0.0161325	0.0575158	0.94	0.5681161	0.6714583	0.97	0.7892515	0.8515609	1.03	0.7934598	0.8952902	1.03	0.7963404	0.7963404
methylhistidine	1.07	0.5592648	0.6744076	0.85	0.2185219	0.4072454	0.94	0.5669478	0.6714583	0.88	0.2991676	0.559726	1.35	0.0193551	0.1552239	0.98	0.8395736	0.8395736
betaala	0.93	0.5936341	0.7054781	1	0.9979192	0.9979192	0.86	0.2340482	0.3690759	0.88	0.3390235	0.5683625	1.03	0.8340677	0.8952902	1.13	0.3336538	0.3336538
sarcosine	1.06	0.6372152	0.7464521	1.09	0.5027711	0.6874337	0.96	0.7034221	0.8011196	0.88	0.3224429	0.5674613	0.95	0.6710425	0.8530708	0.93	0.5043462	0.5043462
lys	1.06	0.6478354	0.7482043	1.17	0.2478711	0.432456	1.08	0.5207917	0.6470443	1.05	0.6716016	0.7975897	0.76	0.0334432	0.1828229	1.03	0.8210561	0.8210561
arg	0.96	0.7253828	0.8261304	0.93	0.544917	0.6874337	0.81	0.0700518	0.1595625	0.9	0.4045299	0.6258765	0.94	0.6070913	0.8296914	1.12	0.3135873	0.3135873
ab	0.96	0.7421823	0.8336843	1.01	0.9108972	0.9678252	0.89	0.3138768	0.4357936	0.86	0.2164228	0.4840289	1.03	0.8262343	0.8952902	1.35	0.006937	0.006937
citrulline	0.96	0.7532621	0.8346959	0.74	0.0224679	0.0708603	0.92	0.4668591	0.5889607	1.06	0.6498423	0.7953294	1.24	0.0763766	0.3131439	1.04	0.7246392	0.7246392
nndimethylglycine	1.03	0.7927607	0.8611343	0.99	0.9442197	0.9678252	0.98	0.859585	0.9274469	1.05	0.6641702	0.7975897	1.21	0.1307437	0.4277458	0.87	0.2274901	0.2274901
nacetylaspartate	0.97	0.8086261	0.8611343	0.87	0.2862458	0.4790235	0.99	0.9480924	0.9513312	1.04	0.7665218	0.849389	1.13	0.3073078	0.599982	0.89	0.2889727	0.2889727
azelate	1.03	0.7997099	0.8611343	0.92	0.4976907	0.6874337	1.02	0.8563819	0.9274469	1.07	0.5400852	0.7210163	1.02	0.8516175	0.8952902	0.97	0.7499186	0.7499186
pelargonate	1.02	0.8390204	0.8820471	0.84	0.1767205	0.3715661	1.06	0.5731961	0.6714583	1.07	0.548299	0.7210163	1.17	0.1932492	0.518983	0.94	0.56432	0.56432
malate	1.01	0.9530853	0.9892784	0.96	0.7266928	0.8512687	0.88	0.2243561	0.3632172	0.83	0.1118293	0.3193628	1.33	0.013918	0.1268087	1.29	0.0171709	0.0171709
guanidinoacetate	1	0.9947685	0.9947685	0.89	0.4414985	0.6582341	1.04	0.8045821	0.8915639	1.05	0.7516738	0.844346	1.15	0.3573413	0.610458	1.06	0.6793411	0.6793411
glutarate	1	0.9764001	0.9947685	0.89	0.3238293	0.5206667	0.99	0.9513312	0.9513312	1.13	0.2771713	0.5479547	1.1	0.4047699	0.6638226	0.97	0.784439	0.784439

indoxylsulfate	1	0.9887784	0.9947685	1.04	0.7791947	0.8874162	1.04	0.734584	0.8251492	1.07	0.5539515	0.7210163	0.89	0.3389133	0.610458	0.92	0.4615473	0.4615473

* Indicates a metabolite among the 25 most significant metabolites in both BLSA and TMCS.
† Indicates a metabolite which is among the 25 most significant metabolites in the TMCS cohort only.

Supplementary Table 4a. Sensitivity analysis results for BLSA cohort. P-values are FDR-adjusted. Model includes smoking status and physical activity as covariates, in addition to age, sex, and sample storage time.

MetS: metabolic syndrome; OR: odds ratio; WC: waist circumference; TG: triglycerides; BP: blood pressure

**Metabolite**	**MetS OR**	**MetS p-value**	**WC OR**	**WC p-value**	**TG OR**	**TG p-value**	**HDL-C OR**	**HDL-C p-value**	**BP OR**	**BP p-value**	**Elevated Glucose OR**	**Elevated Glucose p-value**
alphaaminoadipate*	2.59	0.003504	2.16	0.016959	1.71	0.168767	1.42	0.32553	1.52	0.511848	2.37	0.000293
urate*	2.67	0.003504	3.03	0.000876	1.67	0.19345	1.45	0.328729	1.35	0.872155	2.54	0.00029
oxoglutarate*	2.22	0.007516	1.94	0.016959	2.09	0.059736	2.17	0.030505	1.18	0.930454	1.66	0.015762
hydroxybutyrate_2*	2.06	0.008992	2.01	0.016959	1.43	0.302552	1.56	0.223648	1.5	0.511848	2.17	0.000319
glu*	2.21	0.015586	2.23	0.016959	1.72	0.168767	2.18	0.042489	1.02	0.960241	1.63	0.038901
phe*	1.89	0.017502	1.55	0.100897	1.9	0.059736	1.8	0.073651	1.16	0.930454	1.15	0.45676
asn*	0.53	0.018051	0.54	0.016959	0.7	0.302552	0.76	0.397718	0.76	0.732393	0.93	0.696113
methyl2oxopentanoate*	2.07	0.023766	2.06	0.024128	1.7	0.192184	1.7	0.223648	1.32	0.872155	2.48	0.000442
glucuronate*	1.84	0.023766	1.32	0.418777	1.72	0.093315	1.43	0.288718	1.36	0.732393	1.45	0.062334
gly*	0.51	0.029611	0.58	0.090524	0.54	0.093315	0.84	0.67919	0.6	0.511848	1.14	0.564096
cystine*	1.91	0.031284	1.96	0.029289	1.95	0.081744	1.69	0.192278	1.6	0.511848	1.04	0.861478
ile*	1.97	0.033997	2.03	0.027269	1.39	0.492457	1.63	0.264243	0.87	0.950561	1.75	0.022485
leu*	1.85	0.044079	1.75	0.080657	1.57	0.244738	1.87	0.112628	1.19	0.930454	1.46	0.094507
gln*	0.57	0.049627	0.67	0.218875	0.79	0.553512	0.82	0.592118	0.75	0.832265	0.84	0.415991
oxoisopentanoate*	1.7	0.049627	1.83	0.027269	1.62	0.168767	1.41	0.312099	1.52	0.511848	1.78	0.005911
tyr*	1.66	0.050039	1.9	0.016959	1.76	0.081744	1.45	0.288718	1.33	0.740073	1.3	0.186388

ala*	1.73	0.052472	2.07	0.016959	1.3	0.520403	1.29	0.495407	1.06	0.960241	1.99	0.002691
carnitine*	1.66	0.052472	1.22	0.65523	1.53	0.201019	1.37	0.32553	1.52	0.511848	1.65	0.015992
guanidinoacetate*	0.58	0.094973	0.9	0.779568	0.64	0.271838	0.77	0.530895	0.7	0.732393	1.25	0.338188
pro*	1.59	0.104943	1.46	0.250727	1.32	0.492457	1.47	0.288718	1.57	0.511848	1.82	0.005966
lactate*	1.64	0.108633	3.37	0.000405	1.24	0.609962	1.65	0.223648	1.1	0.959654	1.79	0.013785
pyruvate*	1.44	0.154223	1.63	0.046708	1.44	0.244738	1.29	0.412057	1.02	0.960241	1.31	0.13641
val*	1.47	0.220282	1.58	0.125266	1.48	0.297793	1.47	0.288718	1.04	0.960241	1.45	0.083642
uridine*	1.43	0.274623	1.12	0.75492	1.83	0.081744	1.55	0.264243	0.99	0.967436	1.31	0.203698
betaala	1.4	0.338084	1.16	0.713168	1.23	0.609962	1.24	0.586819	1.16	0.939396	1.4	0.125971
taurine	0.74	0.338084	0.95	0.856218	0.96	0.910765	1.25	0.530895	0.8	0.872155	0.68	0.051472
asp	1.34	0.338084	1.32	0.412959	1.22	0.573631	1.44	0.288718	0.87	0.930454	0.95	0.766987
isocitrate	1.38	0.338084	1.16	0.713168	1.13	0.795265	1.03	0.950985	1.52	0.511848	0.95	0.815975
nacetylaspartate	0.75	0.346995	0.84	0.67359	0.53	0.059736	0.59	0.112628	1.08	0.959654	0.9	0.597693
hydroxyproline	1.35	0.349877	1.34	0.417447	1.11	0.795265	0.91	0.842973	1.37	0.732393	1.3	0.21741
oxoproline	0.72	0.354158	0.9	0.763659	0.59	0.168767	0.79	0.530895	1.06	0.960241	1.24	0.32183
kynurenine	1.31	0.429825	1.42	0.275095	1.31	0.492457	1.36	0.342989	1.48	0.511848	0.93	0.733171
methylhistidine	1.27	0.517071	0.89	0.75492	1.4	0.344524	1.15	0.712023	1.22	0.899208	1.2	0.36131
glycerophosphate	0.72	0.517071	0.59	0.218875	0.7	0.492457	1.02	0.954729	1.03	0.960241	1.08	0.778851
trp	1.28	0.539578	1.22	0.67359	1.23	0.609962	1.52	0.273272	1.18	0.930454	1.45	0.089062
hydroxybutyrate_3	1.24	0.63987	1.05	0.87524	1.32	0.492457	1.24	0.539355	0.98	0.960241	1.2	0.382616
creatine	1.25	0.690127	1.14	0.75492	1.05	0.907318	1.15	0.756692	1.27	0.899208	2.11	0.002712
ab	1.19	0.742011	1.2	0.67359	1.12	0.795265	1.31	0.447818	1.07	0.960241	1.97	0.002501
nndimethylglycine	1.2	0.742011	1.28	0.532224	1.27	0.53401	1.53	0.264243	0.97	0.960241	1.18	0.430743
creatinine	1.28	0.742011	0.86	0.75492	1.22	0.762074	0.94	0.914243	1.24	0.930454	0.84	0.532389
betaine	1.17	0.742011	0.86	0.682365	1.32	0.492457	1.27	0.494304	1.12	0.950561	0.99	0.950471
lys	0.85	0.742011	0.73	0.411068	1.01	0.977739	1.04	0.914243	1.05	0.960241	1.14	0.511906
glycerophosphorylcholine	1.2	0.742011	1.07	0.836301	1.14	0.795265	0.99	0.954729	0.97	0.960241	1.34	0.18873
glutarate	0.82	0.742011	1.25	0.631809	0.84	0.682553	0.87	0.756692	1.22	0.907867	0.95	0.825874
indoxylsulfate	1.18	0.742011	1.17	0.682365	1.2	0.650787	1.09	0.850141	1.12	0.950561	1.24	0.287785
adma	0.85	0.745116	1.23	0.65523	1	0.997248	1.06	0.881884	0.97	0.960241	0.86	0.442076
acetylbutyrate	1.16	0.771813	1.04	0.895267	1.16	0.761385	1.03	0.954729	1.1	0.959654	1.15	0.481276

malate	1.18	0.791959	1.65	0.125266	0.76	0.53401	1.13	0.802358	1.13	0.950561	1.72	0.023626
sdma	0.86	0.83771	0.73	0.470717	1.12	0.795265	1.02	0.954729	0.82	0.930454	0.8	0.33801
gammabutyrobetaine	0.88	0.83876	0.73	0.411068	0.95	0.903294	0.82	0.592118	0.9	0.952525	1	0.981483
cysteinessulfate	1.26	0.83876	1.22	0.75492	0.91	0.903294	1.84	0.32553	0.94	0.960241	1.16	0.70594
mucate	0.88	0.83876	0.94	0.836301	1.01	0.977739	0.95	0.881884	1.29	0.872155	0.83	0.359549
indole3acetate	1.13	0.842548	1.74	0.047869	1.32	0.492457	1.02	0.954729	0.91	0.959654	1.08	0.729786
azelate	1.95	0.842548	0.52	0.75492	4.79	0.492457	2.52	0.67919	1.25	0.960241	5.51	0.14314
pipecolate	1.11	0.849698	0.92	0.779568	1.23	0.573631	1.06	0.881884	1.14	0.939396	1.07	0.744552
ornithine	1.1	0.869292	1.19	0.67359	0.92	0.812255	1.38	0.32553	1.02	0.960241	1.17	0.43854
triethanolamine	1.11	0.869292	1.03	0.920316	1.39	0.492457	1.5	0.312099	0.69	0.732393	1.03	0.901556
his	0.91	0.869292	0.91	0.768352	1.31	0.492457	1.11	0.772402	1.21	0.899208	1.23	0.275153
citrulline	0.9	0.869292	0.81	0.67359	1.12	0.795265	0.98	0.954729	0.8	0.899208	1.16	0.507248
pelargonate	0.86	0.869292	1.25	0.713168	1.17	0.795265	0.9	0.881884	0.72	0.899208	1.33	0.3727
decanoate	1.1	0.869292	0.89	0.75492	1.04	0.910765	0.89	0.772402	1.19	0.930454	1.08	0.723956
thr	0.94	0.932001	1	0.989166	0.91	0.795265	0.89	0.756692	1.24	0.872155	1.17	0.396508
sarcosine	0.96	0.938287	0.88	0.713168	1.25	0.53401	1.13	0.756692	1.08	0.959654	1.11	0.563201
aminoisobutyrate	0.95	0.938287	0.89	0.75492	1.04	0.910765	1.18	0.67919	1.19	0.930454	0.88	0.557099
ser	1.05	0.938287	0.89	0.763659	0.92	0.857854	1.37	0.414364	0.71	0.732393	1.15	0.536164
methylnicotinamide	1.06	0.938287	0.81	0.630215	1.37	0.396693	1.06	0.881884	1.05	0.960241	0.71	0.086909
arg	1.06	0.938287	1.28	0.545127	0.88	0.795265	0.78	0.495407	1.03	0.960241	1.75	0.010666
guanidinosuccinate	0.95	0.938287	0.85	0.682365	0.91	0.795265	0.79	0.530895	0.91	0.959654	0.86	0.476406
fumarate	1.07	0.938287	1.31	0.532224	0.9	0.795265	1.26	0.546855	1.02	0.960241	1.24	0.320285
succinate	1.05	0.938287	1.64	0.125266	0.86	0.795265	0.99	0.954729	1.28	0.899208	1.17	0.503579
threonate	0.94	0.938287	0.99	0.972536	1.24	0.586376	1.45	0.312099	0.89	0.950561	0.96	0.850221
hippurate	0.95	0.938287	1.07	0.836301	1.06	0.886	0.9	0.772402	0.99	0.967436	0.96	0.812896
citrate	0.94	0.938287	0.77	0.470717	0.9	0.795265	0.78	0.494304	1.15	0.939396	0.79	0.223846
trimethylaminenoxide	1.01	0.961168	1.18	0.67359	0.92	0.795265	0.84	0.643462	0.92	0.959654	1.1	0.627912
prolinebetaine	0.97	0.961168	1.22	0.67359	0.83	0.621273	0.92	0.86509	0.89	0.950561	0.88	0.530471
met	1.02	0.961168	0.93	0.836301	0.99	0.977739	0.93	0.881884	1.14	0.950561	1.31	0.253366
oacetylcarnitine	1.02	0.961168	0.84	0.708407	1.1	0.800396	1.1	0.849398	0.92	0.960241	1.17	0.473693
hexanoate	1.01	0.961168	0.96	0.895267	0.75	0.492457	0.82	0.592118	1.45	0.522923	1.2	0.348128

octanoate	1.02	0.961168	0.85	0.682365	1.02	0.977739	0.84	0.67919	0.88	0.950561	1.35	0.144585
terephthalate	1.03	0.961168	0.95	0.856218	0.8	0.53401	1.09	0.842973	1.02	0.960241	1.24	0.270408
choline	1.02	0.971392	1.31	0.67359	0.83	0.795265	1.11	0.881884	1.03	0.960241	1.16	0.646545
cisaconitate	1	0.980559	1.01	0.97549	0.89	0.795265	0.86	0.67919	1.26	0.872155	1.05	0.790358

* Indicates metabolite among the top 25 highest ranked metabolites in original and sensitivity analyses.

Supplementary Table 4b. Sensitivity analysis results for TMCS cohort. P-values are FDR-adjusted. Model includes smoking status, physical activity, and diet quality (DASH score) as covariates, in addition to age and sex.

MetS: metabolic syndrome; OR: odds ratio; WC: waist circumference; TG: triglycerides; BP: blood pressure

Metabolite	MetS OR	MetS p-value	WC OR	WC p-value	TG OR	TG p-value	HDL-C OR	HDL-C p-value	BP OR	BP p-value	Elevated Glucose OR	Elevated Glucose p-value
glu*	2.64	0.00000000	2.38	0.00000036	2.16	0.00000021	1.91	0.00010283	1.13	0.60431881	1.57	0.0001775
pro*	2.22	0.00000024	1.8	0.00082675	1.6	0.00507551	1.57	0.01923624	1.36	0.14417664	1.97	0.0000002
methyl2oxopentanoate*	2.2	0.00000040	1.47	0.03901921	1.75	0.00101217	1.52	0.03583563	1.49	0.06585662	1.83	0.00000345
mucate*	0.52	0.00000227	0.54	0.00025569	0.69	0.00938861	0.71	0.05732442	0.6	0.00572203	0.72	0.00316642
ile*	2.16	0.00000241	1.89	0.00082675	1.63	0.00724953	1.64	0.01923624	1.15	0.5989992	1.51	0.00200291
leu*	2.08	0.00000961	2.09	0.00025569	1.61	0.00773205	1.58	0.03583563	1.19	0.536308	1.66	0.00026311
cystine*	1.85	0.00001077	1.38	0.06422082	1.51	0.0075247	1.77	0.00057377	1.37	0.11633573	1.43	0.00202566
alphaaminoadipate*	1.89	0.00001308	1.69	0.00254758	1.44	0.01843843	1.37	0.13943564	1.25	0.31241677	1.86	0.00000037
oxoisopentanoate*	1.74	0.00002808	1.45	0.02324752	1.42	0.01425581	1.23	0.27361095	1.19	0.42148781	1.69	0.00000517
val*	1.82	0.00008569	1.88	0.00071557	1.4	0.04294591	1.3	0.21560531	1.16	0.54145667	1.95	0.00000046
ala*	1.78	0.00009032	1.82	0.00082675	1.52	0.00938861	1.28	0.21560531	1.17	0.536308	2.12	0.00000001
ser*	0.6	0.00009032	0.62	0.00151072	0.68	0.0075247	0.92	0.66866017	0.72	0.07307326	0.93	0.51979248
gly*	0.6	0.00013582	0.83	0.31971331	0.66	0.00672587	0.8	0.21560531	0.87	0.536308	0.81	0.06369461
phe*	1.67	0.00028291	1.55	0.01110651	1.26	0.14790556	1.32	0.16592128	1.2	0.47839015	1.47	0.00148564
pyruvate*	1.64	0.00028455	1.19	0.37874834	1.57	0.00507551	1.3	0.17473316	1.15	0.54145667	1.37	0.00708499
tyr*	1.65	0.00037221	1.81	0.00071557	1.2	0.2599817	1.15	0.54230726	1.1	0.73072897	1.63	0.00006902

trp*	1.57	0.00301103	1.37	0.09423389	1.39	0.04395856	1.22	0.36558574	0.85	0.536308	1.24	0.0857992
gln*	0.67	0.00335107	0.72	0.04734143	0.7	0.0141255	0.89	0.58179287	0.63	0.00748416	1.13	0.27965159
hydroxybutyrate_3*	0.66	0.00455086	0.62	0.00670049	0.68	0.01418798	0.76	0.17473316	1.13	0.62189875	0.89	0.34851697
hydroxybutyrate_2*	1.43	0.0058531	1.06	0.7565462	1.29	0.07730852	1.21	0.30098789	1.22	0.31241677	1.48	0.00026842
guanidinosuccinate*	0.67	0.00676526	0.67	0.02335239	0.77	0.11541309	0.69	0.05732442	0.92	0.73072897	0.75	0.01593072
isocitrate*	1.41	0.0078071	1.26	0.17456347	1.32	0.04395856	1.33	0.1334042	1.25	0.24875312	1.2	0.08516526
cisaconitate*	1.38	0.01280508	1.23	0.23537282	1.2	0.19955341	1.29	0.16018882	1.34	0.11633573	1.27	0.02615708
cysteinessulfate*	1.75	0.01295669	0.89	0.74426822	1.22	0.42123835	1.38	0.3181341	1.34	0.42148781	1.58	0.01265173
carnitine	1.42	0.01485775	1.13	0.58944292	1.3	0.10284549	1.17	0.48670012	1.34	0.14417664	1.3	0.02273369
lactate	1.37	0.01632867	1.27	0.17456347	1.24	0.13966941	1.06	0.78420522	1.26	0.24875312	1.49	0.00030211
prolinebetaine	0.73	0.02340085	0.99	0.97941508	0.77	0.09817972	0.85	0.48010038	0.96	0.87253997	0.87	0.22390034
methylnicotinamide	1.37	0.02558004	1.24	0.27728975	1.38	0.03313579	1.32	0.16018882	0.89	0.64207586	1.18	0.13997823
urate	1.4	0.02558004	1.4	0.06643281	1.11	0.51402745	1.04	0.88473577	1.54	0.02817807	1.23	0.09474488
citrate	0.74	0.02842781	0.9	0.6207563	0.8	0.14882144	0.84	0.43371405	0.98	0.87929535	0.98	0.855367
acetylbutyrate	0.74	0.03672802	0.85	0.42323082	0.87	0.41306541	0.9	0.63514625	0.65	0.02817807	0.86	0.21512872
decanoate	0.78	0.03672802	1.04	0.83225828	0.81	0.10294496	0.89	0.54230726	0.95	0.83606271	0.91	0.31257922
gammabutyrobetaine	0.75	0.04078593	0.83	0.35750186	0.8	0.14882144	0.91	0.66672024	1.09	0.73072897	0.97	0.7708093
met	1.27	0.06451336	1.19	0.35750186	1.21	0.16504596	1.3	0.16018882	1.22	0.2986555	1.06	0.56278541
choline	1.31	0.06691132	1.22	0.31971331	1.14	0.42123835	0.96	0.88473577	1.36	0.14417664	1.14	0.28074314
asn	0.76	0.0788237	0.59	0.00271157	0.76	0.10658931	0.9	0.66866017	0.73	0.14417664	1.11	0.40751165
threonate	0.78	0.08231969	0.69	0.03465907	0.83	0.21761762	0.87	0.54230726	0.77	0.19479229	0.87	0.2343162
aminoisobutyrate	0.78	0.09809048	0.79	0.23537282	0.7	0.02094377	0.78	0.21560531	1.06	0.83606271	0.97	0.81015559
betaine	0.8	0.09809048	0.65	0.0049452	0.89	0.42123835	0.96	0.8436845	1.03	0.87253997	0.84	0.11359415
adma	0.79	0.09809048	1.08	0.72654379	0.69	0.01425581	0.79	0.21560531	1.09	0.73072897	0.85	0.17653904
glycerophosphate	1.24	0.10568615	1.07	0.72654379	1.32	0.04395856	1.26	0.17776922	1.01	0.90062258	0.84	0.10021517
creatine	1.27	0.1129115	1.39	0.06643281	1.3	0.11426202	1.15	0.54230726	1.14	0.5989992	1.16	0.22059926
thr	1.25	0.12144457	1.09	0.72654379	1.2	0.26732714	1.34	0.15680586	1.09	0.73072897	1.26	0.04891578
glucuronate	1.24	0.12144457	1.21	0.33201496	1.21	0.21687033	1.29	0.17473316	1.18	0.48673411	1.13	0.28741221
succinate	0.82	0.15387944	0.71	0.03505722	0.83	0.19955341	0.86	0.49776373	0.98	0.90062258	1.01	0.93463158
glycerophosphorylcholine	1.2	0.18959284	0.89	0.56557374	1.16	0.33471847	1.07	0.74699525	1.23	0.31241677	0.96	0.70960523
hippurate	0.87	0.25213492	0.98	0.91771705	0.84	0.15248053	0.89	0.49533297	0.96	0.84322506	1.07	0.47478475
uridine	0.85	0.27811066	1.09	0.71371864	0.89	0.42271879	1.01	0.96203809	0.88	0.54145667	0.92	0.44911107
pipecolate	1.18	0.2974047	0.97	0.90851633	0.94	0.68991995	0.89	0.58586768	1.08	0.77473483	1.37	0.00889799

trimethylaminenoxide	0.87	0.36210681	0.91	0.70944136	0.85	0.29118846	0.85	0.45478014	0.93	0.79627603	1.07	0.54612565
terephthalate	0.88	0.45834363	1.05	0.83225828	0.92	0.62889763	1.02	0.90375286	0.84	0.45524655	0.9	0.33949598
hydroxyproline	1.13	0.4792436	0.98	0.93761491	1.02	0.92011798	0.97	0.88473577	1.02	0.90062258	0.99	0.92383448
taurine	1.12	0.4868834	1.39	0.0445761	1.12	0.42123835	1.32	0.15680586	1.14	0.57629548	0.96	0.7372267
sdma	0.89	0.4868834	0.98	0.93761491	0.82	0.19828081	0.81	0.27535167	1.05	0.84419154	0.95	0.67773323
oacetylcarnitine	1.12	0.4868834	1.08	0.72654379	1.18	0.27825409	0.95	0.81590113	1.2	0.42148781	1.17	0.1736462
ornithine	1.12	0.49121407	1.01	0.97941508	0.96	0.81359652	1.03	0.88572635	0.94	0.83606271	1.08	0.48708459
creatinine	0.87	0.50311349	1	0.98980912	0.79	0.22565794	0.87	0.60247446	1.21	0.536308	0.94	0.68073668
triethanolamine	0.92	0.50311349	1.11	0.4685006	1.01	0.92011798	1	0.99226349	0.97	0.87253997	0.88	0.11922928
his	1.12	0.50311349	1.19	0.40481225	1.14	0.42123835	1.05	0.82070676	0.97	0.87929535	1.08	0.53709214
oxoproline	0.9	0.50311349	0.87	0.4685006	0.85	0.30441025	1.08	0.7347356	1.03	0.87253997	1.1	0.39505706
asp	1.1	0.52394535	1.17	0.40481225	1.11	0.44390663	1.03	0.88473577	0.95	0.84322506	1.02	0.82271078
octanoate	0.91	0.55737179	0.85	0.40481225	0.98	0.92011798	1.11	0.58586768	1.17	0.48673411	0.87	0.210154
indole3acetate	0.92	0.57022444	0.94	0.7565462	0.89	0.42123835	0.88	0.54230726	0.97	0.87253997	0.98	0.86906543
kynurenine	1.1	0.57022444	1.4	0.05874894	1.02	0.92011798	1	0.9895794	1.05	0.87253997	0.84	0.14404972
hexanoate	0.92	0.5719347	1.08	0.72654379	0.86	0.31631916	0.93	0.74699525	0.98	0.87929535	1	0.98501085
oxoglutarate	1.08	0.61144602	0.87	0.44134931	1.12	0.42123835	1.13	0.54230726	1.08	0.73072897	1.01	0.93446827
fumarate	0.93	0.65158693	0.73	0.05272217	0.94	0.68362928	0.98	0.88572635	1.04	0.87253997	1.03	0.81019025
methylhistidine	1.07	0.70426923	0.85	0.41155281	0.93	0.67572438	0.87	0.54230726	1.35	0.14417664	0.98	0.84564104
betaala	0.94	0.7109633	1	0.98980912	0.86	0.36223795	0.88	0.58533655	1.04	0.87253997	1.12	0.355246
lys	1.06	0.7340336	1.17	0.44134931	1.08	0.63095466	1.06	0.79603694	0.77	0.19479229	1.02	0.8312358
sarcosine	1.06	0.74273496	1.08	0.72654379	0.96	0.81359652	0.89	0.58179287	0.95	0.86478055	0.92	0.49035048
ab	0.96	0.82661083	1.01	0.98712874	0.89	0.42271879	0.87	0.5134017	1.03	0.87253997	1.35	0.00713671
arg	0.96	0.82661083	0.93	0.74426822	0.81	0.16504596	0.89	0.58533655	0.93	0.77473483	1.13	0.28579463
citrulline	0.96	0.82661083	0.75	0.07554382	0.92	0.59454357	1.05	0.82070676	1.23	0.31241677	1.04	0.7113077
nndimethylglycine	1.04	0.8335904	0.99	0.97941508	0.98	0.92011798	1.06	0.78420522	1.21	0.40606762	0.87	0.22040661
azelate	1.03	0.83878625	0.91	0.65682679	1.03	0.89548857	1.09	0.66866017	1.03	0.87929535	0.97	0.76121497
nacetylaspartate	0.97	0.86413811	0.87	0.50638276	0.99	0.93241037	1.03	0.88473577	1.13	0.5989992	0.89	0.2856236
pelargonate	1.02	0.89756838	0.85	0.37604171	1.06	0.68362928	1.07	0.75592437	1.16	0.53054962	0.94	0.57843152
guanidinoacetate	1	0.99209489	0.9	0.70944136	1.04	0.8819145	1.03	0.88572635	1.13	0.67313285	1.07	0.6282324
glutarate	1	0.99209489	0.89	0.54092631	0.99	0.94803337	1.13	0.54230726	1.09	0.72990326	0.97	0.80776319
malate	1	0.99209489	0.96	0.88134776	0.87	0.34263748	0.82	0.28507795	1.32	0.14417664	1.29	0.01722124
indoxylsulfate	1	0.99209489	1.04	0.8552303	1.04	0.84424596	1.07	0.75237462	0.89	0.5989992	0.92	0.45205397

* Indicates metabolite among 25 highest ranked metabolites in original and sensitivity analyses.

Supplementary Table 5a. Sex-stratified logistic regression: BLSA Male Participants Only. Model includes age and sample storage time as covariates.

Metabolite	MetS OR	MetS p-value	MetS FDR p-value	WC OR	WC p-value	WC FDR p-value	Elevated TGs OR	Elevated TGs p-value	Elevated TGs FDR p-value	Reduced HDL-c OR	Reduced HDL-c p-value	Reduced HDL-c FDR p-value	BP OR	BP p-value	BP FDR p-value	Elevated FG OR	Elevated FG p-value	Elevated FG FDR p-value
alphaaminoadipate*	2.87	0.0005182	0.0227631	1.55	0.1059652	0.4760536	2.19	0.0095168	0.1950934	2.38	0.0027552	0.1498713	1.85	0.030548	0.8349797	1.79	0.0265599	0.0265599
glucuronate*	3	0.0005552	0.0227631	1.9	0.0251289	0.3257099	2.37	0.0059687	0.1631448	1.82	0.0350901	0.2615804	2.22	0.0109745	0.8349797	2.7	0.0012353	0.0012353
urate*	2.67	0.0011812	0.0322856	2.09	0.0123954	0.3257099	1.89	0.0312603	0.3010162	1.31	0.3203896	0.6153199	1.29	0.368971	0.9375829	2.62	0.0014674	0.0014674
glu*	2.6	0.0073975	0.1516492	1.97	0.0520086	0.4738565	1.87	0.0829518	0.3607329	2.84	0.0036554	0.1498713	1.24	0.5526388	0.9375829	1.69	0.1137052	0.1137052
uridine*	2.07	0.0118669	0.1946179	1.15	0.6025297	0.8374141	2.7	0.0017576	0.0862167	2.07	0.0117426	0.1925789	1.36	0.2811878	0.9375829	1.71	0.0501043	0.0501043
hydroxybutyrate_2*	1.81	0.0205951	0.2814665	1.58	0.070924	0.4760536	1.71	0.0470796	0.3509572	1.63	0.0548578	0.2646083	1.35	0.2514264	0.9375829	1.68	0.0342173	0.0342173
ile*	1.99	0.024785	0.2903381	1.74	0.0665591	0.4760536	1.7	0.1066432	0.3860282	2.27	0.0088177	0.1807636	0.92	0.7968721	0.9375829	1.75	0.0512286	0.0512286
asn*	0.58	0.0456432	0.3742741	0.56	0.0317766	0.3257099	0.91	0.7263914	0.891485	1.02	0.9427818	0.954421	0.81	0.4224954	0.9375829	0.73	0.2101272	0.2101272
phe*	1.71	0.0446288	0.3742741	1.51	0.1219586	0.4760536	1.65	0.0731521	0.3607329	1.82	0.0256115	0.2183242	1.09	0.7496855	0.9375829	1.23	0.402108	0.402108
kynurenine	1.76	0.037387	0.3742741	1.54	0.1116768	0.4760536	1.7	0.0641233	0.3607329	1.68	0.0500624	0.2646083	1.78	0.0492713	0.8980987	1.18	0.5044163	0.5044163
creatine	1.65	0.0553838	0.3832439	1.36	0.2322582	0.6339386	1.68	0.0649265	0.3607329	1.58	0.0844555	0.3847415	1.14	0.6040098	0.9375829	1.66	0.0496098	0.0496098
carnitine*	1.67	0.0607582	0.3832439	1.28	0.3588687	0.7356809	1.86	0.0330384	0.3010162	1.34	0.2601642	0.6153199	1.69	0.0685817	0.8980987	1.51	0.114041	0.114041
cystine*	1.68	0.0591507	0.3832439	1.39	0.227749	0.6339386	2.64	0.0021028	0.0862167	1.82	0.0266249	0.2183242	1.56	0.1192872	0.9155642	1.34	0.2553953	0.2553953
taurine	0.62	0.0766625	0.4490233	0.78	0.3506579	0.7356809	0.8	0.4443516	0.814809	1.2	0.4924028	0.7673194	0.85	0.5514529	0.9375829	0.53	0.018379	0.018379

pro*	1.44	0.1229039	0.4912559	1.02	0.924866	0.9479877	1.55	0.0768732	0.3607329	1.44	0.1177798	0.4132523	1.71	0.0304828	0.8349797	1.62	0.0392575	0.0392575
val*	1.57	0.1198799	0.4912559	1.59	0.1116104	0.4760536	1.86	0.0533218	0.3607329	1.84	0.0392984	0.2646083	1.14	0.6550685	0.9375829	1.44	0.1858299	0.1858299
hydroxybutyrate_3	1.53	0.1132676	0.4912559	1	0.9859589	0.9859589	1.8	0.0436825	0.3509572	1.29	0.3332269	0.6153199	1.27	0.3622664	0.9375829	1.27	0.3375159	0.3375159
glutarate*	0.6	0.1186921	0.4912559	0.85	0.5816799	0.8368027	0.97	0.9257279	0.9608821	0.92	0.7979716	0.954421	1.11	0.7326043	0.9375829	0.52	0.0368224	0.0368224
nacetylaspartate	0.66	0.1258094	0.4912559	0.79	0.3966181	0.7477834	0.5	0.0280694	0.3010162	0.46	0.0079543	0.1807636	1.15	0.6281431	0.9375829	0.73	0.2242784	0.2242784
isocitrate*	1.64	0.1218631	0.4912559	1.16	0.6356087	0.851774	1.26	0.4826937	0.8289967	1.23	0.504674	0.7673194	1.92	0.0556614	0.8980987	1.24	0.4741177	0.4741177
indoxylsulfate	1.53	0.1138288	0.4912559	1.51	0.1269946	0.4760536	1.31	0.3396588	0.7753657	0.99	0.9557632	0.9557632	1.09	0.7578329	0.9375829	1.26	0.3543877	0.3543877
leu*	1.54	0.1405881	0.512705	1.94	0.0312418	0.3257099	1.48	0.223692	0.599854	1.99	0.0226942	0.2183242	1.2	0.5469251	0.9375829	1.4	0.2189849	0.2189849
triethanolamine	1.68	0.1438075	0.512705	0.94	0.8595982	0.9398273	1.86	0.1056434	0.3860282	2.46	0.0195627	0.2183242	0.88	0.7204918	0.9375829	1.68	0.1247015	0.1247015
methyl2oxopentanoate*	1.55	0.1508776	0.5154984	1.4	0.2717263	0.6553399	1.24	0.5054858	0.8289967	1.33	0.3331277	0.6153199	1.25	0.4517378	0.9375829	2.96	0.0009521	0.0009521
gln*	0.65	0.1596409	0.5236222	0.7	0.2396597	0.6339386	0.96	0.8919957	0.9473496	1.03	0.9141409	0.954421	0.83	0.533116	0.9375829	0.91	0.7284116	0.7284116
oxoglutarate	1.41	0.1864007	0.5878791	1.18	0.4937006	0.8008577	1.65	0.0883696	0.3623153	1.7	0.0430672	0.2646083	1.04	0.8833677	0.9375829	1.36	0.2082447	0.2082447
guanidinoacetate	0.7	0.2039335	0.6193535	1.19	0.5276761	0.8215456	0.87	0.6419177	0.8586636	0.92	0.7727817	0.954421	0.63	0.1013534	0.9155642	1.1	0.7200527	0.7200527
methylhistidine	1.41	0.2185922	0.6401628	0.79	0.4103689	0.7477834	1.99	0.025933	0.3010162	1.31	0.3270874	0.6153199	1.11	0.7247495	0.9375829	1.33	0.2886845	0.2886845
nndimethylglycine	1.35	0.2286206	0.6464444	1.47	0.1321475	0.4760536	1.23	0.4396562	0.814809	1.31	0.289443	0.6153199	1.51	0.133985	0.9155642	1.14	0.6010285	0.6010285
oxoisopentanoate	1.4	0.2587964	0.7073769	1.7	0.0810393	0.4760536	1.29	0.4341091	0.814809	0.93	0.7968543	0.954421	1.35	0.3206438	0.9375829	2.01	0.0188879	0.0188879
gly	0.74	0.2821344	0.7396209	0.64	0.1169085	0.4760536	0.7	0.2414047	0.599854	1.24	0.421067	0.7431617	0.61	0.0876194	0.8980987	0.78	0.3271542	0.3271542
prolinebetaine	1.27	0.2886326	0.7396209	1.22	0.4012846	0.7477834	0.86	0.4961588	0.8289967	1.08	0.7304986	0.9472311	1.38	0.1856695	0.9375829	0.93	0.7332178	0.7332178
betaala	1.24	0.4090278	0.8228161	1.08	0.7632677	0.9192225	1.41	0.2293871	0.599854	1.29	0.3230881	0.6153199	0.95	0.8537725	0.9375829	1.06	0.8006574	0.8006574
ala	1.22	0.4642354	0.8228161	1.44	0.191081	0.58032	1.25	0.4471513	0.814809	1.3	0.3292363	0.6153199	0.82	0.4830913	0.9375829	1.52	0.1151118	0.1151118
creatinine	1.28	0.3339221	0.8228161	0.95	0.8336079	0.9363815	1.2	0.4950381	0.8289967	0.98	0.9404505	0.954421	1.19	0.5150605	0.9375829	0.95	0.8492586	0.8492586

ornithine	1.16	0.5418545	0.8228161	1.03	0.912625	0.9479877	0.99	0.9788079	0.9991888	1.34	0.2295963	0.6153199	1.15	0.5871348	0.9375829	1.26	0.3310589	0.3310589
asp	1.17	0.5368995	0.8228161	1.46	0.1460983	0.4991693	1	0.9927543	0.9991888	1.32	0.2741987	0.6153199	0.97	0.9208958	0.9558665	0.79	0.3254519	0.3254519
methylnicotinamide	1.19	0.4770784	0.8228161	0.78	0.3383392	0.7301003	1.8	0.030974	0.3010162	1.47	0.1194996	0.4132523	0.95	0.8423023	0.9375829	0.85	0.5112785	0.5112785
citrulline	1.24	0.513932	0.8228161	0.86	0.6335817	0.851774	1.4	0.3404044	0.7753657	1.07	0.8445931	0.954421	0.78	0.4611423	0.9375829	1.1	0.7494456	0.7494456
tyr	1.27	0.3783846	0.8228161	1.53	0.1335272	0.4760536	1.67	0.0835845	0.3607329	1.32	0.3019701	0.6153199	1.36	0.2781785	0.9375829	1.44	0.168191	0.168191
trp	1.31	0.3533616	0.8228161	1.03	0.921483	0.9479877	1.19	0.5699894	0.8407384	2.05	0.0188156	0.2183242	1.07	0.8100994	0.9375829	1.62	0.1000741	0.1000741
pyruvate	1.16	0.5188455	0.8228161	1.22	0.397349	0.7477834	1.22	0.4212423	0.814809	0.97	0.9055742	0.954421	0.91	0.6921398	0.9375829	1.48	0.0854348	0.0854348
lactate	1.2	0.5229569	0.8228161	2.16	0.0126766	0.3257099	1.19	0.5650191	0.8407384	1.33	0.3077528	0.6153199	1.12	0.7141074	0.9375829	1.37	0.2513152	0.2513152
hexanoate	0.85	0.4821674	0.8228161	1.06	0.8024167	0.9267348	0.65	0.1082762	0.3860282	0.62	0.0502801	0.2646083	1.06	0.8143592	0.9375829	1.09	0.6879549	0.6879549
oxoproline	0.86	0.5417506	0.8228161	0.71	0.1671655	0.5483029	0.63	0.0795741	0.3607329	0.87	0.5763935	0.8074547	0.94	0.8061574	0.9375829	1.23	0.3883684	0.3883684
malate	1.21	0.4873991	0.8228161	1.25	0.433331	0.7702891	0.9	0.7284085	0.891485	1.19	0.524023	0.7673194	1.41	0.2521241	0.9375829	1.41	0.201192	0.201192
threonate	1.2	0.4829078	0.8228161	1.21	0.4690252	0.788996	1.39	0.2377174	0.599854	1.47	0.137859	0.4521776	1.04	0.8787982	0.9375829	1.01	0.974612	0.974612
octanoate	0.79	0.393816	0.8228161	0.61	0.0812975	0.4760536	0.92	0.7845394	0.9099239	0.73	0.2718476	0.6153199	0.71	0.2430755	0.9375829	0.87	0.6053152	0.6053152
pelargonate	0.79	0.4634209	0.8228161	1.02	0.9575235	0.9693448	1.36	0.3835055	0.814809	0.81	0.5183024	0.7673194	0.55	0.0870738	0.8980987	1.39	0.2975204	0.2975204
glycerophosphate	0.77	0.4960053	0.8228161	0.39	0.0224679	0.3257099	0.57	0.1652972	0.5462392	1.03	0.9361158	0.954421	1.31	0.4901569	0.9375829	0.94	0.8544168	0.8544168
cisaconitate	1.23	0.4051703	0.8228161	1.39	0.2027339	0.5937208	0.95	0.8543671	0.937977	0.78	0.3237956	0.6153199	1.22	0.4546615	0.9375829	1.68	0.0424189	0.0424189
hippurate	0.84	0.5151715	0.8228161	1.03	0.9011795	0.9479877	1.09	0.762939	0.9066811	0.65	0.1209519	0.4132523	1.22	0.4814917	0.9375829	0.99	0.9653035	0.9653035
azelate	3.23	0.4451099	0.8228161	0.65	0.7847022	0.9192225	6.96	0.2341393	0.599854	2.04	0.6464677	0.8550056	0.99	0.996155	0.996155	9.19	0.1451891	0.1451891
citrate	0.83	0.4654322	0.8228161	0.76	0.28847	0.6641092	0.79	0.3933307	0.814809	0.78	0.3376756	0.6153199	0.96	0.8654206	0.9375829	0.82	0.4348631	0.4348631
trimethylaminenoxide	0.87	0.579714	0.8643009	0.87	0.5730551	0.8368027	0.92	0.7543657	0.9066811	1.15	0.5652952	0.8074547	1.01	0.9752216	0.9872613	1.11	0.6586467	0.6586467
mucate	1.15	0.5944694	0.870473	1.12	0.6647992	0.851774	1.47	0.179446	0.5656627	1.54	0.0977513	0.4132523	1.22	0.4597259	0.9375829	0.7	0.157211	0.157211

aminoisobutyrate	1.12	0.6255769	0.8792114	1.06	0.814159	0.9272367	1.07	0.7878609	0.9099239	1.28	0.3047796	0.6153199	1.19	0.4831851	0.9375829	0.72	0.1713997	0.1713997
pipecolate	0.88	0.6244406	0.8792114	0.73	0.2606118	0.6475808	1	0.9991888	0.9991888	1.05	0.8505069	0.954421	1.44	0.2071498	0.9375829	1.15	0.5990525	0.5990525
hydroxyproline	1.14	0.6326034	0.8792114	1.04	0.8986496	0.9479877	1.05	0.8693446	0.937977	0.81	0.4415304	0.7431617	1.49	0.1711072	0.9375829	1.55	0.1144173	0.1144173
ab	1.13	0.6465546	0.8836246	0.81	0.4415072	0.7702891	1.39	0.2537842	0.6120677	1.55	0.1178827	0.4132523	1.09	0.7590605	0.9375829	1.38	0.2260693	0.2260693
sarcosine	1.11	0.6719988	0.8940828	0.93	0.7585847	0.9192225	1.12	0.6701765	0.8586636	0.98	0.9206081	0.954421	1.23	0.4091087	0.9375829	1.45	0.1258337	0.1258337
thr	0.91	0.6978208	0.8940828	0.85	0.4980944	0.8008577	0.9	0.6646337	0.8586636	1.04	0.8537262	0.954421	1.39	0.1812253	0.9375829	1.04	0.8534265	0.8534265
gammabutyrobetaine	0.91	0.690759	0.8940828	0.72	0.1881435	0.58032	1.14	0.626334	0.8586636	0.87	0.5920217	0.8090963	0.78	0.3307597	0.9375829	0.85	0.4752942	0.4752942
his	1.11	0.6810817	0.8940828	0.89	0.6464412	0.851774	1.42	0.186502	0.5656627	1.35	0.2263196	0.6153199	1.49	0.1277016	0.9155642	1.26	0.341656	0.341656
adma	1.11	0.7172188	0.904799	1.19	0.5594151	0.834037	1.28	0.429127	0.814809	1.35	0.3094191	0.6153199	1.34	0.3396116	0.9375829	0.76	0.3425901	0.3425901
choline	0.88	0.7584937	0.9073942	1.24	0.5981073	0.8374141	0.56	0.1931531	0.5656627	0.77	0.5215178	0.7673194	1.31	0.5211217	0.9375829	0.91	0.8034006	0.8034006
betaine	0.95	0.8335277	0.9073942	0.56	0.0212383	0.3257099	1.42	0.1665363	0.5462392	1.14	0.5809735	0.8074547	1.04	0.8812846	0.9375829	0.72	0.1679463	0.1679463
lys	1.05	0.8409995	0.9073942	0.93	0.7786979	0.9192225	1.1	0.7238965	0.891485	1.5	0.104085	0.4132523	1.01	0.9553785	0.9792629	0.94	0.7801326	0.7801326
met	1.1	0.7597909	0.9073942	0.87	0.6559625	0.851774	1.06	0.8672591	0.937977	1.43	0.2471683	0.6153199	0.94	0.8497855	0.9375829	0.83	0.5455463	0.5455463
guanidinosuccinate	1.06	0.8192334	0.9073942	0.75	0.2915602	0.6641092	0.85	0.5780186	0.8407384	0.58	0.0524718	0.2646083	0.88	0.6445951	0.9375829	0.86	0.5794603	0.5794603
indole3acetate	1.07	0.798317	0.9073942	2.01	0.0130254	0.3257099	1.17	0.5844157	0.8407384	0.81	0.4302314	0.7431617	1.06	0.8383552	0.9375829	1.34	0.2546144	0.2546144
oacetylcarnitine	1.1	0.8093107	0.9073942	0.65	0.2587136	0.6475808	0.83	0.6658729	0.8586636	1.09	0.8141651	0.954421	1.06	0.8918471	0.9375829	1.18	0.6570759	0.6570759
glycerophosphorylcholine	1.07	0.7839469	0.9073942	0.86	0.530999	0.8215456	1.21	0.4614617	0.8226056	1.02	0.9322526	0.954421	0.8	0.372885	0.9375829	1.15	0.5284825	0.5284825
fumarate	0.94	0.8312402	0.9073942	1.23	0.4714732	0.788996	0.84	0.5653681	0.8407384	1.1	0.7393023	0.9472311	0.88	0.6608809	0.9375829	0.92	0.7621542	0.7621542
acetylbutyrate	1.05	0.8255825	0.9073942	1.04	0.8572891	0.9398273	1.15	0.5673844	0.8407384	0.94	0.778498	0.954421	1.06	0.8186699	0.9375829	0.97	0.8740578	0.8740578
cysteinessulfate	1.15	0.8209422	0.9073942	1.2	0.7598854	0.9192225	1.15	0.8310956	0.9335595	1.52	0.4794605	0.7673194	0.73	0.6532325	0.9375829	0.87	0.8085203	0.8085203
sdma	0.96	0.8839254	0.9413231	0.79	0.385045	0.7477834	1.24	0.4383009	0.814809	1.22	0.4531474	0.7431617	1.08	0.7935201	0.9375829	0.71	0.1901234	0.1901234

ser	1.03	0.9062694	0.9527447	0.91	0.7426905	0.9192225	0.94	0.8286215	0.9335595	1.41	0.2132744	0.6153199	0.9	0.6940241	0.9375829	0.83	0.4700148	0.4700148
arg	1.03	0.9287117	0.9639793	1.67	0.1263436	0.4760536	0.81	0.5334925	0.8407384	0.79	0.4528945	0.7431617	0.86	0.6457718	0.9375829	1.84	0.0551004	0.0551004
succinate	1	0.9867073	0.9870297	1.63	0.0831024	0.4760536	1.04	0.9011374	0.9473496	0.97	0.8951163	0.954421	1.08	0.7793989	0.9375829	0.99	0.9643986	0.9643986
terephthalate	1	0.9870297	0.9870297	1.14	0.550553	0.834037	0.89	0.6204191	0.8586636	1.11	0.6253677	0.8406583	0.81	0.3579771	0.9375829	1.18	0.4192675	0.4192675
decanoate	1.01	0.96745	0.9870297	0.76	0.3154817	0.6991757	1.13	0.6600199	0.8586636	0.96	0.8830534	0.954421	0.87	0.596507	0.9375829	1.12	0.6623014	0.6623014

* Indicates metabolite among top 25 highest ranked metabolites in original and sex-stratified models.

Supplementary Table 5b. Sex-stratified logistic regression: BLSA Female Participants Only. Model includes age and sample storage time as covariates.

**Metabolite**	**MetS OR**	**MetS p-value**	**MetS FDR p-value**	**WC OR**	**WC p-value**	**WC FDR p-value**	**Elevated TGs OR**	**Elevated TGs p-value**	**Elevated TGs FDR p-value**	**Reduced HDL-c OR**	**Reduced HDL-c p-value**	**Reduced HDL-c FDR p-value**	**BP OR**	**BP p-value**	**BP FDR p-value**	**Elevated FG OR**	**Elevated FG p-value**	**Elevated FG FDR p-value**
oxoglutarate*	3.54	0.0002729	0.0223807	3.33	0.0004488	0.0099357	2.37	0.0081423	0.2484786	1.91	0.028082	0.5888033	1.29	0.3768372	0.9394137	1.84	0.055938	0.055938
urate*	3.24	0.0008552	0.0350612	4.64	8.59e-05	0.0070457	2.09	0.0237426	0.250609	1.9	0.0396526	0.5888033	1.64	0.1277726	0.9394137	2.22	0.0269403	0.0269403
alphaaminoadipate*	2.46	0.0020493	0.0353913	3	0.0004847	0.0099357	1.67	0.0638724	0.37411	1.19	0.4853944	0.842831	1.36	0.2549922	0.9394137	2.22	0.0110715	0.0110715
phe*	2.43	0.002158	0.0353913	1.7	0.0466983	0.2127369	2.13	0.0090907	0.2484786	1.55	0.0899467	0.5888033	1.23	0.4334733	0.9394137	1.07	0.8081893	0.8081893
oxoisopentanoate*	2.73	0.0019132	0.0353913	2.16	0.0150823	0.0951346	2.81	0.0016489	0.1352111	2.15	0.0112368	0.5888033	2	0.0261882	0.9394137	1.56	0.1708617	0.1708617
leu*	2.38	0.0046124	0.0508663	1.85	0.038376	0.185108	1.8	0.0464952	0.3493174	1.64	0.0760904	0.5888033	1.2	0.5253069	0.9394137	1.67	0.1062129	0.1062129
tyr*	2.1	0.0061158	0.0508663	2.26	0.0041215	0.0559184	1.85	0.0208433	0.250609	1.36	0.2127201	0.6840215	1.16	0.5629594	0.9394137	1.4	0.2308458	0.2308458
cystine*	2.57	0.0043142	0.0508663	2.63	0.0057363	0.0583231	1.48	0.1748368	0.5749453	1.72	0.0531355	0.5888033	1.42	0.2245503	0.9394137	1.03	0.9151056	0.9151056
hydroxybutyrate_2*	2.3	0.0062032	0.0508663	2.29	0.0079502	0.0592652	1.44	0.2004766	0.6322723	1.41	0.2108984	0.6840215	1.69	0.0747568	0.9394137	2.75	0.0034275	0.0034275
methyl2oxopentanoate*	2.21	0.0058548	0.0508663	2.31	0.0047735	0.0559184	1.89	0.0217293	0.250609	1.45	0.1533711	0.6773984	1.32	0.3176296	0.9394137	1.64	0.0981005	0.0981005

gln*	0.43	0.0105035	0.0724815	0.53	0.055257	0.2384774	0.62	0.1343849	0.4975073	0.7	0.2416164	0.6840215	0.6	0.1097841	0.9394137	0.63	0.1923096	0.1923096
pyruvate*	2.02	0.010607	0.0724815	2.17	0.0064013	0.0583231	1.87	0.0244497	0.250609	1.65	0.0568171	0.5888033	1.29	0.3440203	0.9394137	1.22	0.488174	0.488174
pro*	1.91	0.0115193	0.0726602	2.03	0.0076863	0.0592652	1.52	0.097741	0.4975073	1.42	0.1439057	0.6773984	1.44	0.1555544	0.9394137	1.61	0.0833127	0.0833127
ala*	2.18	0.0161318	0.0837953	2.46	0.0091993	0.062862	1.46	0.2158186	0.6467106	1.24	0.4404246	0.842831	1.26	0.4434433	0.9394137	1.99	0.0574121	0.0574121
guanidinoacetate*	0.49	0.0146646	0.0837953	0.61	0.0864342	0.3316198	0.54	0.0303077	0.2761368	0.61	0.0713472	0.5888033	0.78	0.3623157	0.9394137	1.11	0.7207337	0.7207337
lactate*	2.15	0.0163503	0.0837953	4.38	0.000178	0.0072986	1.44	0.2264617	0.6467106	2.01	0.0239499	0.5888033	1	0.9896118	0.9995988	2.08	0.0382615	0.0382615
betaala	2.13	0.0209731	0.1011644	1.37	0.3175994	0.6677731	1.63	0.1322102	0.4975073	1.52	0.1744905	0.6840215	1.1	0.7628113	0.9394137	1.43	0.2931747	0.2931747
ile*	2	0.0292518	0.133258	2.08	0.0253736	0.138709	1.32	0.3808372	0.7435394	1.07	0.8234556	0.887878	0.91	0.7660543	0.9394137	1.7	0.1128373	0.1128373
carnitine*	1.91	0.0311244	0.1343265	1.21	0.5075064	0.7532822	1.7	0.0734661	0.4016148	1.42	0.2205798	0.6840215	1.49	0.1845906	0.9394137	1.58	0.1519636	0.1519636
asp	1.68	0.0367648	0.1507357	1.46	0.1250673	0.3971274	1.46	0.1209971	0.4975073	1.48	0.0933469	0.5888033	0.95	0.8190578	0.9394137	1.32	0.2901131	0.2901131
isocitrate*	1.78	0.040616	0.1585957	1.55	0.1259185	0.3971274	1.21	0.4922732	0.7867849	0.84	0.5183682	0.842831	1.75	0.0529976	0.9394137	1.04	0.901698	0.901698
glu*	1.78	0.0444167	0.1655531	2.61	0.0032697	0.0536237	1.37	0.2448432	0.6467106	1.36	0.248731	0.6840215	1.07	0.8007107	0.9394137	1.58	0.1328036	0.1328036
gly*	0.58	0.053178	0.1895499	0.67	0.1555139	0.4250714	0.65	0.1200684	0.4975073	0.86	0.5553423	0.842831	0.77	0.3535432	0.9394137	1.38	0.2927337	0.2927337
val*	1.85	0.055478	0.1895499	1.75	0.0889712	0.3316198	1.66	0.1119302	0.4975073	1.32	0.3615654	0.842831	1.14	0.6920742	0.9394137	1.89	0.0735149	0.0735149
asn*	0.61	0.0593199	0.1945691	0.55	0.0286302	0.1467296	0.75	0.2634459	0.6467106	0.72	0.1996096	0.6840215	0.76	0.3150693	0.9394137	1.09	0.7395033	0.7395033
glucuronate	1.54	0.0623359	0.1965977	1.17	0.5047582	0.7532822	1.41	0.1395447	0.4975073	1.09	0.6955866	0.842831	1.11	0.6640435	0.9394137	1.09	0.720776	0.720776
mucate	0.58	0.0668632	0.2030661	0.64	0.1450381	0.4250714	0.71	0.2464562	0.6467106	0.6	0.0846946	0.5888033	1.11	0.7300999	0.9394137	0.95	0.8612608	0.8612608
oxoproline	0.49	0.0715589	0.2095654	1.31	0.4891766	0.7532822	0.46	0.0579725	0.37411	0.58	0.1569582	0.6773984	0.67	0.277789	0.9394137	1.4	0.4314399	0.4314399
nndimethylglycine	1.66	0.0973152	0.2672156	1.57	0.1529418	0.4250714	1.85	0.0468597	0.3493174	1.61	0.1093205	0.6403056	1.06	0.8396561	0.9394137	1.87	0.0712914	0.0712914
hydroxyproline	1.59	0.0977618	0.2672156	1.56	0.1243202	0.3971274	1.25	0.4282162	0.7633419	0.91	0.7194899	0.842831	1.29	0.3641615	0.9394137	1.04	0.8956829	0.8956829
glutarate	0.74	0.1141724	0.3020045	1.04	0.8500923	0.9548982	0.6	0.0137662	0.250609	0.65	0.0315756	0.5888033	0.94	0.7384127	0.9394137	1.16	0.4957471	0.4957471

his	0.68	0.1402084	0.3592839	1	0.9869053	0.9870159	1.11	0.6982864	0.857229	0.81	0.3920518	0.842831	0.85	0.5499909	0.9394137	1.01	0.9676204	0.9676204
creatinine	1.57	0.1578526	0.3922397	1.19	0.581062	0.794118	1.43	0.2668602	0.6467106	0.95	0.8662224	0.887878	1.15	0.6648129	0.9394137	1.14	0.6999551	0.6999551
cisaconitate	1.49	0.1731014	0.4174799	1.3	0.377884	0.7206159	1.12	0.6971016	0.857229	0.8	0.4106277	0.842831	1.62	0.0976695	0.9394137	1.15	0.6843279	0.6843279
citrulline	0.7	0.2113067	0.4950615	0.75	0.3283098	0.6730351	0.95	0.8393602	0.9301018	0.84	0.5036682	0.842831	0.73	0.2567072	0.9394137	1.35	0.3263181	0.3263181
triethanolamine	0.75	0.2508534	0.551218	1.08	0.7548852	0.9266366	1.02	0.9443614	0.9802232	0.96	0.8594695	0.887878	0.65	0.105033	0.9394137	0.81	0.4510665	0.4510665
uridine	1.36	0.2592063	0.551218	1.45	0.1859522	0.462063	1.7	0.0610311	0.37411	1.37	0.2308169	0.6840215	0.86	0.5991183	0.9394137	1.12	0.6906025	0.6906025
glycerophosphate	0.66	0.2621647	0.551218	0.76	0.4716979	0.7532822	0.71	0.3671944	0.7343888	1.03	0.9429815	0.9544836	0.82	0.5978079	0.9394137	1.25	0.5953221	0.5953221
azelate	5.81	0.2508341	0.551218	1.61	0.7598491	0.9266366	5.18	0.2987837	0.6630946	2.32	0.5788114	0.842831	3.81	0.4041225	0.9394137	14.29	0.1016193	0.1016193
threonate	0.72	0.2755784	0.5649357	0.71	0.2738048	0.6068106	1.28	0.4236154	0.7633419	1.38	0.279599	0.7395845	0.66	0.1829379	0.9394137	0.91	0.7807334	0.7807334
citrate	1.33	0.2835433	0.5670865	0.95	0.843062	0.9548982	1.13	0.6471657	0.857229	0.74	0.2502518	0.6840215	1.35	0.2606754	0.9394137	0.88	0.6501567	0.6501567
methylhistidine	1.32	0.3203283	0.6254029	0.98	0.9553441	0.9870159	1.33	0.2992012	0.6630946	1.17	0.5657875	0.842831	1.14	0.6407099	0.9394137	1.08	0.7911057	0.7911057
lys	0.77	0.3472144	0.6327017	0.63	0.1070229	0.3815598	1.13	0.6564293	0.857229	0.75	0.2900846	0.7433418	1.2	0.5249744	0.9394137	1.36	0.3282165	0.3282165
fumarate	1.36	0.340304	0.6327017	1.48	0.2445652	0.5898337	1.29	0.4279498	0.7633419	1.5	0.1993176	0.6840215	1.15	0.6816147	0.9394137	1.22	0.5782221	0.5782221
hexanoate	1.3	0.3373454	0.6327017	0.68	0.1655895	0.438011	1.11	0.6976692	0.857229	1.11	0.6949426	0.842831	1.75	0.0457334	0.9394137	1.15	0.6401901	0.6401901
methylnicotinamide	0.77	0.3662831	0.6521727	0.79	0.4386988	0.7532822	1.04	0.8800868	0.9525044	0.7	0.2158579	0.6840215	0.95	0.8646341	0.9453333	0.5	0.0477122	0.0477122
succinate	1.35	0.3738063	0.6521727	1.99	0.0600904	0.2463708	0.95	0.882809	0.9525044	0.87	0.6670288	0.842831	1.54	0.2166688	0.9394137	2.13	0.0562919	0.0562919
pipecolate	1.2	0.4488255	0.7389272	1	0.9870159	0.9870159	1.32	0.2681483	0.6467106	0.94	0.8032229	0.887878	1.15	0.5804345	0.9394137	1.04	0.8851381	0.8851381
prolinebetaine	0.8	0.4505653	0.7389272	1.21	0.5328094	0.7532822	0.9	0.7108728	0.857229	0.89	0.6858741	0.842831	0.9	0.7397723	0.9394137	0.85	0.6144253	0.6144253
arg	1.2	0.4363428	0.7389272	1.06	0.792548	0.9418686	1.09	0.7004439	0.857229	0.86	0.5054994	0.842831	1.09	0.7240175	0.9394137	1.45	0.1666297	0.1666297
adma	0.86	0.5085288	0.8176345	1.16	0.5327071	0.7532822	1.14	0.5867913	0.857229	1.13	0.5797921	0.842831	0.71	0.169053	0.9394137	0.76	0.2668747	0.2668747
malate	1.23	0.5327517	0.8401085	2.31	0.0223577	0.1309524	0.78	0.4471062	0.7634059	1.12	0.7175375	0.842831	0.94	0.8411423	0.9394137	1.61	0.2031505	0.2031505

ser	0.88	0.6426187	0.863848	1.04	0.9023714	0.9782536	0.72	0.2603782	0.6467106	1.11	0.7092261	0.842831	0.63	0.1287843	0.9394137	1.93	0.0619676	0.0619676
betaine	1.13	0.6131468	0.863848	0.99	0.9604354	0.9870159	1.06	0.8191013	0.9301018	1.22	0.3963727	0.842831	1	0.9995988	0.9995988	1.39	0.2276959	0.2276959
indole3acetate	1.18	0.5797282	0.863848	1.32	0.3702491	0.7206159	1.26	0.4533622	0.7634059	0.93	0.8093972	0.887878	0.87	0.6414363	0.9394137	1.03	0.9258652	0.9258652
glycerophosphorylcholine	0.85	0.5638481	0.863848	1.01	0.9774505	0.9870159	0.9	0.7002952	0.857229	0.85	0.5420068	0.842831	0.97	0.9185995	0.9911205	0.91	0.7509332	0.7509332
acetylbutyrate	1.14	0.6244749	0.863848	0.97	0.906674	0.9782536	1.09	0.7514608	0.8678843	1.12	0.6733582	0.842831	1.1	0.7191157	0.9394137	1.6	0.1329498	0.1329498
octanoate	1.16	0.6135755	0.863848	1.22	0.5022206	0.7532822	0.99	0.9675177	0.9812076	0.8	0.4256502	0.842831	1.13	0.6901839	0.9394137	1.76	0.0912441	0.0912441
pelargonate	1.33	0.5920176	0.863848	1.55	0.4276786	0.7532822	1.48	0.4785897	0.7848871	1.17	0.7682627	0.8872893	1.02	0.9730224	0.9995988	1.35	0.6083833	0.6083833
decanoate	1.15	0.6324734	0.863848	0.83	0.5172392	0.7532822	1.27	0.4037961	0.7633419	0.82	0.4852041	0.842831	1.22	0.4940273	0.9394137	0.96	0.8921044	0.8921044
indoxylsulfate	1.15	0.5935161	0.863848	1.02	0.9540654	0.9870159	1.45	0.1752882	0.5749453	1.23	0.4210603	0.842831	1.05	0.8450378	0.9394137	1.04	0.9024527	0.9024527
sarcosine	0.93	0.7641125	0.9080758	0.92	0.7443625	0.9266366	1.48	0.1279861	0.4975073	1.16	0.5333415	0.842831	0.93	0.7804722	0.9394137	0.96	0.8799956	0.8799956
ab	1.1	0.7318167	0.9080758	1.56	0.1465854	0.4250714	1.1	0.739713	0.8665209	1.12	0.6894379	0.842831	1.01	0.9756784	0.9995988	2.29	0.0173349	0.0173349
taurine	0.92	0.7596345	0.9080758	0.95	0.8436146	0.9548982	1.12	0.6633439	0.857229	1.25	0.3983474	0.842831	0.89	0.6744416	0.9394137	0.83	0.5072292	0.5072292
creatine	0.92	0.7616011	0.9080758	0.9	0.6979126	0.9083942	0.82	0.4561816	0.7634059	0.9	0.6817181	0.842831	1.38	0.2574403	0.9394137	1.81	0.0693185	0.0693185
kynurenine	1.09	0.7428824	0.9080758	1.46	0.1809503	0.462063	1.16	0.590375	0.857229	0.95	0.8535722	0.887878	1.14	0.6361327	0.9394137	0.94	0.8219646	0.8219646
hydroxybutyrate_3	1.13	0.6876736	0.9080758	1.07	0.8140148	0.9535602	1.06	0.8390382	0.9301018	1.13	0.67438	0.842831	0.78	0.4294396	0.9394137	1.52	0.188422	0.188422
terephthalate	0.91	0.7348754	0.9080758	0.77	0.3896578	0.7261805	0.75	0.3362737	0.707037	0.95	0.8649301	0.887878	1.09	0.7623785	0.9394137	0.95	0.8716244	0.8716244
hippurate	0.89	0.7015845	0.9080758	1.44	0.2673769	0.6068106	0.76	0.3647869	0.7343888	0.78	0.3959454	0.842831	0.93	0.8117656	0.9394137	0.86	0.6444793	0.6444793
ornithine	0.94	0.8101895	0.9138783	1.2	0.5040559	0.7532822	0.91	0.7393813	0.8665209	1.49	0.1238268	0.67692	0.82	0.4573933	0.9394137	1.03	0.9183063	0.9183063
met	0.93	0.8135746	0.9138783	0.9	0.7364195	0.9266366	1.03	0.9134281	0.9602705	0.65	0.136882	0.6773984	1.11	0.7279138	0.9394137	1.8	0.0863845	0.0863845
oacetylcarnitine	1.07	0.8024419	0.9138783	1.4	0.2720379	0.6068106	1	0.9989156	0.9989156	0.9	0.7187662	0.842831	0.98	0.9436805	0.9942817	1.02	0.9569554	0.9569554
cysteinessulfate	1.05	0.7882276	0.9138783	0.97	0.8634397	0.9567845	0.98	0.8990086	0.9573858	1.04	0.8381843	0.887878	0.88	0.5309167	0.9394137	0.78	0.3829889	0.3829889

trp	1.06	0.8253974	0.9146295	1.18	0.5723358	0.794118	1.33	0.3220421	0.6949329	1.02	0.9544836	0.9544836	1.2	0.5394997	0.9394137	1	0.989795	0.989795
aminoisobutyrate	0.96	0.8766879	0.9463527	0.8	0.4640074	0.7532822	1.21	0.5085317	0.7867849	1.25	0.4370628	0.842831	0.98	0.9457801	0.9942817	1.21	0.560514	0.560514
choline	1.07	0.8819854	0.9463527	1.62	0.3393206	0.6786411	0.83	0.6936157	0.857229	1.25	0.6303842	0.842831	0.86	0.7569459	0.9394137	1.7	0.356939	0.356939
gammabutyrobetaine	1.04	0.8886483	0.9463527	0.78	0.4263409	0.7532822	1.01	0.9692416	0.9812076	0.83	0.5031017	0.842831	0.84	0.5463529	0.9394137	1.16	0.6406343	0.6406343
trimethylaminenoxide	1.03	0.9112283	0.9469972	1.3	0.2861495	0.6174805	0.88	0.5749504	0.857229	0.67	0.0913512	0.5888033	0.9	0.6660484	0.9394137	1.15	0.6042212	0.6042212
sdma	0.97	0.912351	0.9469972	0.9	0.6866032	0.9080881	1.18	0.5082971	0.7867849	1.05	0.8285817	0.887878	0.74	0.2403919	0.9394137	1.24	0.4392508	0.4392508
nacetylaspartate	0.98	0.9325742	0.9558886	0.84	0.5124913	0.7532822	0.75	0.2777149	0.6506463	0.89	0.6515842	0.842831	0.95	0.8477636	0.9394137	1.25	0.4687519	0.4687519
thr	0.98	0.9468146	0.9585036	1.08	0.7684303	0.9266366	1.14	0.5999851	0.857229	0.83	0.443821	0.842831	1.06	0.808759	0.9394137	1.32	0.3225564	0.3225564
guanidinosuccinate	0.99	0.9670176	0.9670176	1.12	0.6745828	0.9068162	1.14	0.6403164	0.857229	1.21	0.477073	0.842831	1.09	0.7499071	0.9394137	0.95	0.8697846	0.8697846

* Indicates metabolite among top 25 highest ranked metabolites in original and sex-stratified models.

Supplementary Table 5c. Sex-stratified logistic regression: TMCS Male Participants Only. Model includes age as a covariate.

**Metabolite**	**MetS OR**	**MetS p-value**	**MetS FDR p-value**	**WC OR**	**WC p-value**	**WC FDR p-value**	**Elevated TGs OR**	**Elevated TGs p-value**	**Elevated TGs FDR p-value**	**Reduced HDL-c OR**	**Reduced HDL-c p-value**	**Reduced HDL-c FDR p-value**	**BP OR**	**BP p-value**	**BP FDR p-value**	**Elevated FG OR**	**Elevated FG p-value**	**Elevated FG FDR p-value**
glu*	2.75	1e-06	8.57e-05	2.09	0.0028914	0.0592737	2.45	7e-06	0.0005701	2	0.0014172	0.1162065	1	0.989881	0.9972243	1.75	0.0015479	0.0015479
pro*	1.94	8.11e-05	0.0024216	1.97	0.0010988	0.0450526	1.76	0.0005138	0.0092671	1.7	0.0031787	0.1303283	0.99	0.9731995	0.9972243	1.49	0.0077319	0.0077319
mucate*	0.49	8.86e-05	0.0024216	0.52	0.0026251	0.0592737	0.54	0.0005651	0.0092671	0.67	0.035506	0.4852484	0.55	0.0024691	0.1214105	0.76	0.0934127	0.0934127
methyl2oxopentanoate*	2.12	0.0001623	0.0033278	1.97	0.0059247	0.0809715	1.86	0.0013459	0.0183945	1.29	0.2327827	0.6582132	1.73	0.0060153	0.1233128	2.13	5.33e-05	5.33e-05
isocitrate*	2.01	0.000258	0.0042317	1.75	0.0199643	0.1278572	1.48	0.027367	0.1380811	1.54	0.0342646	0.4852484	1.52	0.0254807	0.2415086	1.5	0.0160698	0.0160698
leu*	1.9	0.0006732	0.0092006	1.67	0.0286774	0.1679676	1.6	0.009134	0.0576145	1.43	0.07426	0.4898	1.26	0.2033795	0.555904	1.63	0.0038166	0.0038166

ala*	1.9	0.0009818	0.0115015	2.07	0.0037816	0.0620179	1.72	0.0041065	0.0306121	1.48	0.0629032	0.4898	1.38	0.0891659	0.4216744	1.83	0.0006993	0.0006993
octanoate	0.54	0.0013378	0.0137122	0.7	0.1290395	0.4069706	0.68	0.0338409	0.1540441	0.87	0.4903865	0.8042338	1.2	0.331817	0.7558053	0.86	0.3529009	0.3529009
gly*	0.54	0.0028383	0.0258602	0.83	0.4593949	0.6726853	0.47	0.0003074	0.0092671	0.96	0.8584834	0.9839056	0.68	0.0557152	0.3807204	0.78	0.1608585	0.1608585
val*	1.69	0.0034078	0.0279437	1.57	0.0470581	0.2411729	1.43	0.0375717	0.1540441	1.19	0.3767546	0.7396741	1.17	0.38107	0.8012241	1.79	0.0004641	0.0004641
oxoisopentanoate*	1.64	0.0039775	0.0296502	1.73	0.0116294	0.1192009	1.34	0.0774425	0.2618805	1.01	0.9435925	0.9839056	1.35	0.0812747	0.4216744	2.06	2.02e-05	2.02e-05
ile*	1.72	0.0059633	0.0375935	1.68	0.0381779	0.2087061	1.52	0.0286266	0.1380811	1.47	0.0782995	0.4898	1.09	0.6370379	0.9198292	1.31	0.1256947	0.1256947
alphaaminoadipate*	1.67	0.0064184	0.0375935	1.26	0.3262123	0.5613922	1.42	0.0524816	0.1956131	1.21	0.3494966	0.7396741	1.13	0.5168464	0.8451574	1.61	0.0056969	0.0056969
pyruvate*	1.66	0.0058693	0.0375935	1.31	0.2390604	0.5026398	1.79	0.0015738	0.0184364	1.2	0.3635673	0.7396741	1.28	0.1807473	0.5335269	1.23	0.2034005	0.2034005
ser*	0.61	0.007104	0.0388355	0.69	0.1066565	0.3975378	0.52	0.0003623	0.0092671	1.09	0.6628694	0.8841883	0.67	0.026507	0.2415086	0.96	0.8214274	0.8214274
lactate*	1.57	0.0102332	0.0493902	1.72	0.0147294	0.1255316	1.54	0.0136179	0.079762	1.11	0.5856019	0.8774242	1.77	0.0029612	0.1214105	1.64	0.0033184	0.0033184
cisaconitate*	1.58	0.0102394	0.0493902	1.5	0.0735154	0.3349037	1.14	0.4545817	0.6012209	1.37	0.1135968	0.5813716	1.62	0.0083272	0.1365664	1.44	0.0230938	0.0230938
tyr*	1.56	0.0130614	0.0595018	2.24	0.0007212	0.0450526	1.25	0.1994505	0.3634431	1.17	0.4392185	0.7396741	0.97	0.8771841	0.9972243	1.38	0.0545048	0.0545048
cystine*	1.58	0.0174156	0.0751621	1.43	0.1456552	0.4265617	1.09	0.6435529	0.7508404	1.38	0.1355648	0.5813716	1.37	0.1019079	0.4216744	1.14	0.4593525	0.4593525
acetylbutyrate	0.64	0.0192553	0.0789469	0.95	0.8375801	0.9281293	0.75	0.1161074	0.2912645	0.7	0.0836244	0.4898	0.67	0.034629	0.2581437	0.96	0.794602	0.794602
gln*	0.66	0.0253904	0.0991437	0.94	0.7727024	0.9003749	0.56	0.0024834	0.0254544	0.96	0.8610095	0.9839056	0.66	0.0294553	0.2415331	1.07	0.6830154	0.6830154
threonate	0.68	0.0269365	0.1003997	0.59	0.0201423	0.1278572	0.75	0.1022275	0.2775732	0.9	0.5885163	0.8774242	0.58	0.0045008	0.123023	0.88	0.4235634	0.4235634
decanoate	0.71	0.029599	0.1055269	1.23	0.2817106	0.5368316	0.73	0.0369431	0.1540441	0.8	0.1987207	0.6582132	1.06	0.7023964	0.9289758	0.94	0.6392986	0.6392986
his	1.47	0.0345326	0.1179864	1.76	0.0153087	0.1255316	1.21	0.2804363	0.4312915	1.01	0.9455432	0.9839056	1.17	0.4035127	0.8036663	1	0.9838884	0.9838884
hydroxybutyrate_2*	1.46	0.0409824	0.1344221	1.03	0.911895	0.9692614	1.44	0.0451325	0.1762317	1.26	0.2619864	0.7160962	1.29	0.1727165	0.5335269	1.44	0.0297886	0.0297886
trp	1.46	0.0461792	0.1456421	1.37	0.1953213	0.4730107	1.37	0.0862289	0.2618805	1.18	0.4328979	0.7396741	0.88	0.497346	0.8451574	1.02	0.9086656	0.9086656
phe	1.41	0.0505955	0.1536604	1.53	0.0583682	0.281541	1.25	0.1989577	0.3634431	1.27	0.2283498	0.6582132	0.98	0.8925115	0.9972243	1.26	0.1504757	0.1504757

methylnicotinamide	1.43	0.0582021	0.1590856	1.99	0.0078172	0.0915733	1.27	0.1868024	0.3634431	1.18	0.4234607	0.7396741	0.99	0.97313	0.9972243	1.19	0.3217287	0.3217287
gammabutyrobetaine	0.71	0.0559189	0.1590856	0.93	0.7645565	0.9003749	0.63	0.0088045	0.0576145	0.76	0.1677044	0.6250801	1.23	0.255279	0.6338839	1.03	0.8543008	0.8543008
citrulline	0.72	0.0569016	0.1590856	0.82	0.3663236	0.5832479	0.6	0.0035815	0.029368	0.92	0.6572093	0.8841883	1.07	0.6853907	0.9289758	0.97	0.8650805	0.8650805
choline	1.38	0.0615751	0.1628761	1.1	0.642806	0.8330408	1.27	0.1597218	0.3446629	0.93	0.7183492	0.9203849	1.33	0.1028913	0.4216744	1.37	0.0491562	0.0491562
asp	1.38	0.0688978	0.1765506	1.29	0.2516732	0.51593	1.35	0.0895644	0.2622957	1.21	0.3424455	0.7396741	0.95	0.8016645	0.9960074	1.07	0.6809821	0.6809821
hexanoate	0.73	0.0779384	0.1936651	1	0.9852202	0.9852202	0.79	0.175204	0.3538038	0.74	0.1488878	0.5813716	1.04	0.8438608	0.9972243	1.09	0.5915317	0.5915317
oxoproline	0.73	0.0854047	0.205976	0.94	0.7973777	0.9081246	0.73	0.0844966	0.2618805	1.11	0.6130699	0.8841883	1.02	0.9049082	0.9972243	1	0.9964665	0.9964665
carnitine	1.38	0.0929121	0.2116331	1.37	0.1961264	0.4730107	1.29	0.1766076	0.3538038	0.92	0.6793154	0.8841883	1.4	0.0778105	0.4216744	1.26	0.1756068	0.1756068
succinate	0.74	0.0913651	0.2116331	0.84	0.4524922	0.6726853	0.77	0.1464093	0.3430162	0.83	0.3539649	0.7396741	0.83	0.3277705	0.7558053	0.89	0.5052912	0.5052912
cysteinessulfate	1.63	0.0998715	0.2213368	0.96	0.9164838	0.9692614	1.15	0.619966	0.7367711	1.52	0.2119271	0.6582132	0.86	0.6156569	0.9178885	1.44	0.1658033	0.1658033
hydroxybutyrate_3	0.74	0.1114407	0.2404773	0.74	0.2203512	0.4883459	0.66	0.0275118	0.1380811	0.85	0.430869	0.7396741	1.1	0.6023659	0.9147038	0.94	0.7210901	0.7210901
betaine	0.75	0.1211593	0.2487587	0.69	0.1231021	0.403775	0.73	0.0839527	0.2618805	0.69	0.0752459	0.4898	1.24	0.2514826	0.6338839	0.84	0.3121875	0.3121875
creatine	1.32	0.1213457	0.2487587	1.27	0.2989254	0.5447085	1.34	0.0959959	0.2714366	1.19	0.3844622	0.7396741	1.01	0.9770963	0.9972243	1.18	0.3113215	0.3113215
hydroxyproline	1.3	0.1358525	0.2519213	0.75	0.2280083	0.4920178	1.28	0.1541115	0.3446629	1.27	0.2186485	0.6582132	0.89	0.5359409	0.8451574	0.81	0.2042843	0.2042843
triethanolamine	1.24	0.1382495	0.2519213	1.27	0.2019455	0.4731295	1.16	0.2892809	0.4312915	1.27	0.1446164	0.5813716	1.22	0.1463598	0.5000627	0.96	0.7337198	0.7337198
arg	0.76	0.1273905	0.2519213	1.36	0.1758757	0.4730107	0.59	0.0034622	0.029368	0.73	0.1250223	0.5813716	0.78	0.1670629	0.5335269	1.05	0.7499456	0.7499456
oacetylcarnitine	1.32	0.1326364	0.2519213	1.24	0.3698645	0.5832479	1.25	0.2172602	0.3798348	0.81	0.3196893	0.7396741	1.29	0.1886864	0.5335269	1.18	0.3368885	0.3368885
terephthalate	0.75	0.1305719	0.2519213	1.15	0.5585469	0.7762856	0.87	0.4407991	0.6012209	1.2	0.3815544	0.7396741	0.88	0.480746	0.8451574	0.78	0.1504097	0.1504097
pipecolate	1.27	0.1584342	0.2824262	1.1	0.6758017	0.8525498	0.93	0.6810733	0.7547028	0.92	0.6699349	0.8841883	1.16	0.4076762	0.8036663	1.08	0.6345405	0.6345405
glutarate	1.28	0.163356	0.285004	0.99	0.9574411	0.9692614	1.25	0.196505	0.3634431	1.72	0.0080094	0.2189234	0.86	0.4116339	0.8036663	1.16	0.3634675	0.3634675
sarcosine	1.26	0.1778203	0.3037764	1.08	0.7334288	0.898975	1.04	0.8141066	0.8669707	0.99	0.9783734	0.9839056	0.97	0.8603455	0.9972243	1	0.9871804	0.9871804

pelargonate	0.79	0.2043726	0.3420112	0.74	0.2109787	0.4805627	0.83	0.3138751	0.4596029	0.72	0.1305513	0.5813716	1.34	0.1284261	0.4786793	1.11	0.5250441	0.5250441
prolinebetaine	0.79	0.2160121	0.3473136	0.76	0.2671872	0.5222631	0.74	0.1049362	0.2775732	0.91	0.63522	0.8841883	1.13	0.5315778	0.8451574	0.98	0.8910957	0.8910957
urate	1.25	0.2153656	0.3473136	1.12	0.625859	0.8277491	1.03	0.8770648	0.9220424	0.96	0.8597714	0.9839056	1.31	0.1417585	0.5000627	1.31	0.1015802	0.1015802
glycerophosphorylcholine	1.24	0.2441104	0.3849433	0.78	0.288056	0.5368316	1.33	0.1172162	0.2912645	1.33	0.178361	0.6358956	1.37	0.1011156	0.4216744	0.96	0.824229	0.824229
taurine	1.22	0.2850529	0.4174088	1.51	0.0819129	0.3498573	1.25	0.2177102	0.3798348	1.51	0.0475609	0.4898	1.06	0.7437613	0.9382835	0.99	0.9568189	0.9568189
guanidinosuccinate	0.81	0.2850596	0.4174088	0.79	0.3488612	0.5721323	0.83	0.3384344	0.4868706	0.68	0.0792854	0.4898	0.84	0.3753526	0.8012241	0.88	0.4666303	0.4666303
fumarate	0.82	0.270365	0.4174088	0.88	0.5873046	0.8026496	0.91	0.5983165	0.7214994	1.01	0.9566534	0.9839056	1.35	0.1079898	0.4216744	0.95	0.7813276	0.7813276
oxoglutarate	1.18	0.2832198	0.4174088	1.17	0.4281026	0.6500818	1.18	0.2832015	0.4312915	1.15	0.4262312	0.7396741	1.17	0.3415781	0.7570109	1.16	0.3077423	0.3077423
citrate	0.84	0.316377	0.4551388	1.46	0.0895976	0.3498573	0.83	0.2886119	0.4312915	0.93	0.7343255	0.9263798	1.12	0.5377379	0.8451574	0.97	0.8723388	0.8723388
aminoisobutyrate	0.83	0.3434897	0.4856234	0.72	0.1842604	0.4730107	0.71	0.0741838	0.2618805	0.89	0.5819339	0.8774242	1	0.9947113	0.9972243	0.8	0.2173973	0.2173973
sdma	1.18	0.3596492	0.4983133	0.98	0.9312818	0.9692614	1.14	0.4501107	0.6012209	1.18	0.4001542	0.7396741	0.92	0.6506109	0.9198292	1.02	0.9240793	0.9240793
glucuronate	1.17	0.3646195	0.4983133	1.43	0.1164284	0.403775	1.24	0.2311912	0.3868914	1.43	0.0744787	0.4898	1.16	0.4322397	0.8242711	1.06	0.7216988	0.7216988
betaala	1.16	0.410337	0.5340894	1.51	0.0861291	0.3498573	1.02	0.9204002	0.9553521	1.01	0.9694348	0.9839056	1.08	0.6921045	0.9289758	1.24	0.2044963	0.2044963
met	1.15	0.4041553	0.5340894	1.42	0.1353706	0.4111257	0.99	0.9567	0.9685112	1.1	0.6190416	0.8841883	1.33	0.0828662	0.4216744	1.01	0.9641975	0.9641975
indoxylsulfate	1.16	0.410081	0.5340894	1.26	0.3286198	0.5613922	1.13	0.4667515	0.6075178	1.13	0.5435346	0.8571123	1.02	0.9000777	0.9972243	1.05	0.741932	0.741932
trimethylaminenoxide	0.87	0.4578932	0.5776499	0.84	0.4718036	0.678735	0.88	0.4847847	0.6211304	0.72	0.1295544	0.5813716	1.02	0.9186677	0.9972243	1.07	0.6975324	0.6975324
methylhistidine	1.14	0.4516971	0.5776499	1.05	0.830574	0.9281293	0.99	0.9409798	0.9645043	0.91	0.663193	0.8841883	1.14	0.49796	0.8451574	0.88	0.4529073	0.4529073
lys	1.13	0.48187	0.598687	1.36	0.1874784	0.4730107	1	0.9833135	0.9833135	0.95	0.7887564	0.9653437	0.65	0.025516	0.2415086	1.04	0.8240446	0.8240446
uridine	1.13	0.4954634	0.6063881	1.42	0.1222399	0.403775	0.93	0.6978469	0.7629793	1.28	0.2091366	0.6582132	1.12	0.5462603	0.8451574	1.09	0.6159949	0.6159949
adma	0.9	0.533654	0.643524	1.07	0.7795929	0.9003749	0.82	0.2637054	0.4312915	1.14	0.5035619	0.8096486	1	0.9972243	0.9972243	0.99	0.9495874	0.9495874
glycerophosphate	1.1	0.5817326	0.6913344	1.01	0.9529066	0.9692614	1.27	0.1567588	0.3446629	0.99	0.9759328	0.9839056	1.22	0.2628299	0.6338839	0.81	0.1745815	0.1745815

kynurenine	1.1	0.5963701	0.698605	1.73	0.02027	0.1278572	1.11	0.5483449	0.6711087	1.01	0.9760421	0.9839056	0.89	0.5367574	0.8451574	0.83	0.2461374	0.2461374
azelate	1.08	0.6547467	0.7561864	0.83	0.3833477	0.5931039	1.26	0.1769019	0.3538038	1.52	0.0294314	0.4852484	0.96	0.8158795	0.9972243	0.92	0.5830889	0.5830889
creatinine	0.93	0.6785224	0.7727617	1.26	0.3121624	0.5564634	0.77	0.1356053	0.3270482	0.94	0.7596447	0.943801	1.03	0.8532831	0.9972243	1.05	0.7533669	0.7533669
ab	1.06	0.7442473	0.8360038	0.87	0.5558288	0.7762856	0.89	0.5473176	0.6711087	1	0.9839056	0.9839056	1.07	0.7404651	0.9382835	1.35	0.0910694	0.0910694
guanidinoacetate	0.95	0.7754104	0.8592386	0.98	0.9441628	0.9692614	0.83	0.2842388	0.4312915	0.86	0.4420004	0.7396741	0.93	0.6687821	0.9289758	1.16	0.363864	0.363864
asn	1.05	0.7864771	0.8598816	1.01	0.9500228	0.9692614	0.86	0.3750782	0.5212952	1.2	0.3496239	0.7396741	0.73	0.0809805	0.4216744	1.22	0.220327	0.220327
nndimethylglycine	0.97	0.8719363	0.9407734	0.92	0.7384661	0.898975	0.93	0.6676837	0.7508404	1.02	0.9077597	0.9839056	1.07	0.726891	0.9382835	0.95	0.7687658	0.7687658
thr	0.98	0.927474	0.9510357	1.13	0.5972817	0.8029033	0.93	0.668431	0.7508404	1.19	0.3803915	0.7396741	1.01	0.9756016	0.9972243	1.02	0.9105684	0.9105684
ornithine	0.99	0.9394377	0.9510357	1.28	0.2675006	0.5222631	0.86	0.3667134	0.5184569	1.01	0.9701572	0.9839056	0.79	0.1831724	0.5335269	0.92	0.5905841	0.5905841
indole3acetate	0.99	0.9377638	0.9510357	1.26	0.3442591	0.5721323	0.94	0.7178488	0.774521	0.96	0.8511773	0.9839056	0.79	0.2168613	0.5736332	1.06	0.7158251	0.7158251
nacetylaspartate	1.02	0.9253068	0.9510357	0.69	0.1594088	0.4507421	1.09	0.6624036	0.7508404	1.19	0.4367109	0.7396741	1.16	0.4770656	0.8451574	0.93	0.7061221	0.7061221
hippurate	0.98	0.9135053	0.9510357	0.92	0.6501782	0.8330408	0.84	0.2237255	0.3821978	1.01	0.9743715	0.9839056	1.07	0.639603	0.9198292	1.05	0.7162367	0.7162367
malate	1	0.9793603	0.9793603	1.08	0.7454915	0.898975	0.89	0.5141407	0.6486083	0.83	0.3350275	0.7396741	1.54	0.0197557	0.2415086	1.23	0.2047574	0.2047574

* Indicates metabolite among top 25 highest ranked metabolites in original and sex-stratified analyses.

Supplementary Table 5d. Sex-stratified logistic regression: TMCS Female Participants Only. Model includes age as a covariate.

Metabolite	MetS OR	MetS p-value	MetS FDR p-value	WC OR	WC p-value	WC FDR p-value	Elevated TGs OR	Elevated TGs p-value	Elevated TGs FDR p-value	Reduced HDL-c OR	Reduced HDL-c p-value	Reduced HDL-c FDR p-value	BP OR	BP p-value	BP FDR p-value	Elevated FG OR	Elevated FG p-value	Elevated FG FDR p-value
glu*	2.43	4e-07	2.97e-05	2.59	2e-07	1.92e-05	1.88	0.0001629	0.0066799	1.76	0.0006828	0.0186621	1.21	0.2561995	0.6565113	1.37	0.0482356	0.0482356
pro*	2.19	8e-07	3.39e-05	1.54	0.005053	0.0295961	1.39	0.0224203	0.1521035	1.39	0.0252878	0.1644181	1.57	0.004145	0.1699456	2.11	1.9e-06	1.9e-06

methyl2oxopentanoate*	2.05	7.2e-06	0.0001979	1.25	0.1526482	0.4037792	1.69	0.0007088	0.019373	1.71	0.0005282	0.0186621	1.23	0.1922805	0.6519156	1.51	0.0067459	0.0067459
cystine*	2.08	1.44e-05	0.0002942	1.35	0.0702021	0.2398572	1.93	9.67e-05	0.0066799	2.08	1.92e-05	0.0015748	1.39	0.0500278	0.4558085	1.75	0.0007032	0.0007032
ile*	1.86	4.28e-05	0.0007025	1.55	0.004195	0.0280418	1.42	0.0154249	0.1521035	1.45	0.0113264	0.1203674	1.11	0.4895443	0.7955428	1.45	0.0111854	0.0111854
leu*	1.88	0.0001022	0.0013973	1.93	1e-04	0.0033918	1.46	0.01726	0.1521035	1.45	0.0197008	0.1615466	1.12	0.484904	0.7955428	1.61	0.0028719	0.0028719
alphaaminoadipate*	1.78	0.0001655	0.0019382	1.67	0.0013877	0.0162556	1.36	0.0385086	0.1754279	1.39	0.0260663	0.1644181	1.36	0.0491221	0.4558085	1.78	0.0001651	0.0001651
val*	1.75	0.0003388	0.0033231	1.84	0.0002026	0.0038113	1.29	0.0917038	0.3133213	1.25	0.1383013	0.4200262	1.16	0.345577	0.7658734	1.91	5.1e-05	5.1e-05
oxoisopentanoate*	1.78	0.0003647	0.0033231	1.33	0.0822891	0.2595272	1.44	0.0215826	0.1521035	1.44	0.0227178	0.1644181	1.08	0.6339653	0.7965831	1.55	0.0064909	0.0064909
guanidinosuccinate*	0.57	0.0006039	0.004952	0.61	0.0028312	0.0211051	0.71	0.0296654	0.1715092	0.67	0.0117432	0.1203674	0.95	0.7527027	0.8817375	0.68	0.0156084	0.0156084
ser*	0.6	0.0007864	0.0058619	0.59	0.0007733	0.0105685	0.81	0.1586005	0.410798	0.86	0.2998789	0.5372029	0.77	0.0883024	0.5363373	0.93	0.5952978	0.5952978
phe*	1.68	0.0011269	0.0077007	1.6	0.0044457	0.0280418	1.21	0.2285388	0.4461947	1.22	0.200731	0.473351	1.18	0.3031179	0.710162	1.47	0.0148428	0.0148428
tyr*	1.58	0.0012623	0.0079619	1.58	0.002132	0.0194252	1.16	0.2862378	0.4835096	1.19	0.2018545	0.473351	1.08	0.5958743	0.7965831	1.5	0.0044179	0.0044179
mucate*	0.63	0.0016945	0.009925	0.68	0.0132124	0.0601899	0.73	0.0329516	0.1715092	0.75	0.0549608	0.2371993	0.68	0.0143787	0.2155525	0.73	0.0282704	0.0282704
gly*	0.62	0.0020735	0.0113349	0.84	0.2718633	0.6064603	0.76	0.0788476	0.3109306	0.73	0.0424434	0.2047268	0.98	0.9060251	0.9393345	0.83	0.2323897	0.2323897
pyruvate*	1.56	0.0031396	0.0160902	1.11	0.5097395	0.7759641	1.37	0.0334652	0.1715092	1.34	0.0485864	0.2213379	0.97	0.8235833	0.9126193	1.43	0.0174971	0.0174971
ala*	1.54	0.0034875	0.0168221	1.48	0.0099412	0.0479517	1.22	0.1753406	0.410798	1.14	0.3609941	0.56631	1.02	0.8976611	0.9393345	2.02	6.1e-06	6.1e-06
trp*	1.54	0.0051928	0.0224109	1.24	0.1799602	0.4471737	1.3	0.0869051	0.3109306	1.21	0.2106268	0.473351	0.93	0.6605811	0.7965831	1.3	0.0852344	0.0852344
hydroxybutyrate_3*	0.63	0.0049855	0.0224109	0.58	0.0017653	0.0180942	0.72	0.0448524	0.1935733	0.74	0.07349	0.2896793	1.12	0.5179321	0.7955428	0.91	0.5417691	0.5417691
gln*	0.68	0.0069432	0.028467	0.57	0.0002324	0.0038113	0.82	0.1714416	0.410798	0.86	0.3070353	0.5372029	0.59	0.0007524	0.0616957	1.15	0.327812	0.327812
hydroxybutyrate_2*	1.47	0.008098	0.0316206	1.12	0.4340137	0.7660635	1.23	0.1544986	0.410798	1.2	0.2036996	0.473351	1.24	0.1664998	0.6501421	1.68	0.0005421	0.0005421
asn	0.68	0.0089233	0.0332596	0.54	0.0001241	0.0033918	0.77	0.0799759	0.3109306	0.83	0.1987067	0.473351	0.82	0.212337	0.6529865	1.04	0.7734048	0.7734048
thr	1.45	0.0129747	0.0462577	1.1	0.5315095	0.7759641	1.46	0.0126887	0.1521035	1.45	0.0141369	0.1288027	1.13	0.4229692	0.7955428	1.46	0.011456	0.011456

urate	1.44	0.0174327	0.058084	1.36	0.0527962	0.1882299	1.22	0.2046739	0.4329051	1.15	0.3660297	0.56631	1.56	0.007606	0.2078984	1.08	0.5954561	0.5954561
cysteinessulfate*	1.83	0.0177085	0.058084	0.86	0.578934	0.7824992	1.26	0.3591814	0.5367759	1.27	0.3537936	0.56631	1.89	0.0165329	0.2155525	1.75	0.0307675	0.0307675
prolinebetaine	0.7	0.0212539	0.0630499	1.09	0.5707265	0.7824992	0.79	0.1277533	0.3879914	0.84	0.2650465	0.5054375	0.87	0.3974712	0.7760152	0.81	0.1692083	0.1692083
triethanolamine	0.77	0.022179	0.0630499	1.05	0.6478534	0.817292	0.94	0.6058729	0.7306114	0.89	0.3144603	0.5372029	0.82	0.0968018	0.5363373	0.83	0.095576	0.095576
uridine	0.72	0.0222981	0.0630499	0.93	0.642173	0.817292	0.92	0.5526931	0.6866794	0.94	0.665313	0.8183695	0.75	0.062286	0.4805872	0.79	0.1019285	0.1019285
citrate	0.71	0.0205214	0.0630499	0.71	0.0243504	0.0998366	0.82	0.1810762	0.4124513	0.85	0.2778094	0.5177357	0.91	0.5252775	0.7955428	0.95	0.7314813	0.7314813
carnitine	1.35	0.0461792	0.121405	1	0.9911762	0.9911762	1.19	0.2399018	0.4574871	1.24	0.1510688	0.427385	1.18	0.2939818	0.709015	1.35	0.0481012	0.0481012
adma	0.75	0.0473776	0.121405	1.1	0.5393897	0.7759641	0.64	0.0025512	0.0523002	0.66	0.0053362	0.109393	1.14	0.3758007	0.7703915	0.78	0.0908805	0.0908805
glycerophosphate	1.34	0.0451787	0.121405	1.11	0.5082818	0.7759641	1.4	0.024114	0.1521035	1.47	0.0101747	0.1203674	0.91	0.5643689	0.7965831	0.81	0.1526047	0.1526047
sdma	0.74	0.0496853	0.1234604	0.99	0.9319432	0.9911762	0.64	0.0049022	0.0803966	0.66	0.00726	0.1190645	1.15	0.3944782	0.7760152	0.9	0.5059398	0.5059398
aminoisobutyrate	0.73	0.0559722	0.1345154	0.82	0.248858	0.5830388	0.69	0.0217785	0.1521035	0.72	0.0415746	0.2047268	1.11	0.5392058	0.7955428	1.15	0.3744813	0.3744813
lactate	1.31	0.0574151	0.1345154	1.12	0.4278293	0.7660635	1.1	0.4815122	0.6580667	1.06	0.6776797	0.8183695	1.05	0.7417225	0.8814674	1.38	0.0215201	0.0215201
met	1.28	0.0595983	0.1357517	1.07	0.6249433	0.8134183	1.32	0.0365542	0.1754279	1.34	0.0294718	0.1726206	1.17	0.2532883	0.6565113	1.17	0.2290191	0.2290191
betaine	0.79	0.0773941	0.1586579	0.65	0.002374	0.019467	0.88	0.332102	0.5236994	0.99	0.9157725	0.9503276	0.84	0.2150077	0.6529865	0.8	0.0892224	0.0892224
cisaconitate	1.28	0.0739981	0.1586579	1.13	0.3869715	0.7660635	1.27	0.0872122	0.3109306	1.27	0.0940258	0.3409117	1.25	0.1288616	0.580587	1.13	0.3734307	0.3734307
hippurate	0.81	0.0720623	0.1586579	1	0.9776216	0.9911762	0.85	0.1531512	0.410798	0.83	0.122208	0.3854254	0.89	0.3424455	0.7658734	1.07	0.549581	0.549581
glucuronate	1.33	0.0768715	0.1586579	1.17	0.3551476	0.7467207	1.2	0.2508639	0.4675192	1.25	0.1662079	0.4543016	1.17	0.370639	0.7703915	1.14	0.398258	0.398258
methylnicotinamide	1.31	0.0800288	0.1600576	0.99	0.9457674	0.9911762	1.48	0.0144727	0.1521035	1.41	0.0319797	0.1748225	0.82	0.2390243	0.6565113	1.19	0.2718949	0.2718949
ornithine	1.24	0.1395647	0.2661466	0.89	0.4499981	0.7660635	1.06	0.6950794	0.7818402	1.05	0.7544186	0.8713004	1.12	0.472427	0.7955428	1.26	0.1128519	0.1128519
glutarate	0.8	0.1364265	0.2661466	0.91	0.522307	0.7759641	0.85	0.2635365	0.4724917	0.87	0.3442446	0.56631	1.31	0.0981105	0.5363373	0.77	0.0853337	0.0853337
succinate	0.81	0.1495503	0.2725138	0.65	0.0058324	0.0318839	0.79	0.1111008	0.3644106	0.81	0.1511483	0.427385	1.1	0.531628	0.7955428	1.08	0.6000067	0.6000067

octanoate	1.22	0.1473623	0.2725138	0.92	0.5821031	0.7824992	1.19	0.2279067	0.4461947	1.25	0.1143541	0.3799426	1.25	0.1345263	0.580587	0.91	0.5063182	0.5063182
decanoate	0.83	0.1700207	0.3030803	0.93	0.6060519	0.8015525	0.88	0.3555001	0.5367759	0.97	0.8284267	0.9179864	0.83	0.1795223	0.6519156	0.86	0.260565	0.260565
gammabutyrobetaine	0.83	0.1776584	0.3099573	0.85	0.2740942	0.6064603	0.9	0.4525014	0.6289002	0.94	0.6786479	0.8183695	1.02	0.8829051	0.9393345	0.98	0.8677063	0.8677063
acetylbutyrate	0.82	0.1960622	0.3349396	0.82	0.2280041	0.5498921	0.97	0.8587368	0.914499	1.02	0.8806175	0.9375862	0.64	0.0124975	0.2155525	0.82	0.1978237	0.1978237
pelargonate	1.19	0.2144333	0.3588476	0.89	0.4114029	0.7660635	1.23	0.1350208	0.395418	1.28	0.0741862	0.2896793	1.07	0.6567161	0.7965831	0.83	0.1821511	0.1821511
creatine	1.2	0.2278991	0.3737545	1.38	0.0436623	0.1627412	1.19	0.2681093	0.4724917	1.09	0.5654735	0.7824659	1.13	0.4423641	0.7955428	1.2	0.2368445	0.2368445
indoxylsulfate	0.85	0.2690288	0.4325561	0.95	0.7521339	0.8810711	0.94	0.6646373	0.7785751	0.98	0.8648404	0.9331173	0.75	0.0703298	0.4805872	0.8	0.1242113	0.1242113
choline	1.16	0.2793438	0.4403224	1.22	0.1733961	0.4443276	0.99	0.9498135	0.9615396	0.94	0.6703177	0.8183695	1.25	0.1292761	0.580587	0.94	0.6612479	0.6612479
citrulline	1.18	0.2845986	0.4403224	0.68	0.0186879	0.0806531	1.23	0.1730837	0.410798	1.12	0.4539934	0.6647761	1.28	0.1262499	0.580587	1.11	0.4929111	0.4929111
betaala	0.86	0.3079657	0.4676516	0.89	0.4479453	0.7660635	0.82	0.1741932	0.410798	0.86	0.3135676	0.5372029	1.01	0.9490862	0.9608033	1.04	0.7807742	0.7807742
isocitrate	1.15	0.3148445	0.4694046	1.12	0.4572378	0.7660635	1.25	0.1214618	0.3830718	1.25	0.1158362	0.3799426	1.07	0.6498757	0.7965831	1.01	0.9309997	0.9309997
trimethylaminenoxide	0.87	0.3323168	0.4729082	0.95	0.708242	0.8416788	0.83	0.1947244	0.4315515	0.89	0.4287676	0.6392535	0.89	0.4560426	0.7955428	1.1	0.4823714	0.4823714
creatinine	0.87	0.3303393	0.4729082	0.9	0.4689294	0.7690442	0.84	0.2058939	0.4329051	0.84	0.2135852	0.473351	1.22	0.1864073	0.6519156	0.87	0.3043177	0.3043177
nacetylaspartate	0.87	0.334496	0.4729082	0.99	0.9720272	0.9911762	0.84	0.2201743	0.4461947	0.88	0.3547116	0.56631	1	0.98287	0.98287	0.86	0.302032	0.302032
arg	1.15	0.3637682	0.5055761	0.79	0.1285948	0.3646025	1.02	0.8808766	0.9215565	1.03	0.8475894	0.9266978	1.09	0.5772512	0.7965831	1.25	0.1406842	0.1406842
glycerophosphorylcholine	1.12	0.4426235	0.6049188	0.97	0.8460786	0.9465876	1.06	0.6922031	0.7818402	0.95	0.7232448	0.8595083	1.08	0.6247433	0.7965831	0.97	0.8544048	0.8544048
ab	0.9	0.4656864	0.6260046	1.07	0.6656047	0.8269634	0.89	0.4178346	0.5907317	0.84	0.2257805	0.4776082	1.04	0.7934621	0.8912861	1.45	0.012523	0.012523
taurine	1.11	0.4835289	0.639506	1.4	0.0335145	0.1308661	1.11	0.4975806	0.6688788	1.28	0.0956216	0.3409117	1.2	0.2535144	0.6565113	1	0.9935829	0.9935829
indole3acetate	0.89	0.4996055	0.6502802	0.93	0.6983949	0.8416788	0.83	0.2889265	0.4835096	0.86	0.3733264	0.5669031	1.1	0.6123061	0.7965831	0.91	0.5941037	0.5941037
kynurenine	1.1	0.51573	0.6607791	1.26	0.1289448	0.3646025	0.96	0.7945268	0.8804216	0.99	0.9520254	0.9520254	1.19	0.2671543	0.663838	0.9	0.4763998	0.4763998
nndimethylglycine	1.09	0.545501	0.6881705	0.96	0.7954375	0.9186743	1.02	0.8923694	0.9215565	1.08	0.6040413	0.8119899	1.34	0.0653152	0.4805872	0.89	0.3998201	0.3998201

pipecolate	1.08	0.6140143	0.7628662	0.9	0.510293	0.7759641	0.85	0.3039687	0.4985086	0.81	0.1923184	0.473351	0.96	0.7920658	0.8912861	1.64	0.0018842	0.0018842
his	0.94	0.6612056	0.7973362	1.1	0.5570741	0.7824992	1.03	0.8312722	0.8992321	1.04	0.7791827	0.8874025	0.9	0.5378627	0.7955428	1.12	0.4456446	0.4456446
terephthalate	0.94	0.6599335	0.7973362	1.03	0.8369681	0.9465876	0.92	0.5841224	0.7148961	0.9	0.4979799	0.7163922	0.82	0.1987548	0.6519156	0.97	0.860239	0.860239
oxoproline	1.06	0.6922456	0.8226686	0.85	0.2810426	0.6064603	0.98	0.8990795	0.9215565	1.07	0.6286911	0.8183695	1.02	0.8778815	0.9393345	1.17	0.2918117	0.2918117
hydroxyproline	0.95	0.7023095	0.8227054	1.03	0.8542376	0.9465876	0.86	0.2708184	0.4724917	0.84	0.2271551	0.4776082	1.03	0.8421232	0.9207213	1.06	0.6700828	0.6700828
sarcosine	0.95	0.7159151	0.8268315	1.12	0.4572662	0.7660635	0.87	0.3600326	0.5367759	0.84	0.2462368	0.4992152	0.89	0.4497792	0.7955428	0.92	0.557778	0.557778
hexanoate	1.04	0.7736717	0.8811261	1.1	0.5335672	0.7759641	0.93	0.6331281	0.7524131	1.07	0.6590743	0.8183695	0.93	0.6491279	0.7965831	0.89	0.4341362	0.4341362
asp	0.97	0.7956401	0.8816552	1.06	0.6944881	0.8416788	0.97	0.8334346	0.8992321	0.98	0.8918503	0.9375862	0.96	0.7896269	0.8912861	0.97	0.8244624	0.8244624
lys	0.96	0.7945898	0.8816552	1	0.9777964	0.9911762	1.09	0.5418318	0.6835417	1.03	0.8218618	0.9179864	0.87	0.3679591	0.7703915	1.01	0.9482039	0.9482039
oacetylcarnitine	0.97	0.8592317	0.9369547	1	0.9885491	0.9911762	1.1	0.5285789	0.6772417	1.01	0.9458554	0.9520254	1.09	0.5813929	0.7965831	1.12	0.4413625	0.4413625
fumarate	1.02	0.8699846	0.9369547	0.67	0.0077907	0.0399272	1	0.9981365	0.9981365	0.99	0.9271488	0.9503276	0.83	0.2325589	0.6565113	1.05	0.7535601	0.7535601
threonate	0.98	0.8915702	0.9369547	0.8	0.1397051	0.3818605	0.91	0.5230785	0.6772417	0.94	0.6583988	0.8183695	1.02	0.9164239	0.9393345	0.93	0.5933973	0.5933973
oxoglutarate	1.02	0.8836604	0.9369547	0.76	0.0766163	0.2513014	1.06	0.6960285	0.7818402	1.1	0.5138666	0.7265011	1.1	0.5432975	0.7955428	0.87	0.3314362	0.3314362
azelate	1.02	0.9026758	0.9369547	0.98	0.8778428	0.9597748	0.91	0.5119104	0.6770428	0.92	0.572536	0.7824659	1.08	0.5984752	0.7965831	0.99	0.920748	0.920748
malate	0.99	0.9442143	0.9678196	0.89	0.4438156	0.7660635	0.87	0.3151804	0.5067606	0.85	0.2625838	0.5054375	1.1	0.5252199	0.7955428	1.39	0.0247182	0.0247182
guanidinoacetate	1	0.9914451	0.9914451	0.89	0.4577696	0.7660635	1.14	0.3808266	0.5576389	1.05	0.7343089	0.8601904	1.24	0.1632284	0.6501421	0.96	0.7977798	0.7977798
methylhistidine	1	0.9831753	0.9914451	0.77	0.0887015	0.2693896	0.88	0.3942864	0.5672191	0.84	0.2496076	0.4992152	1.47	0.0184008	0.2155525	1.07	0.6222803	0.6222803

* Indicates metabolite among top 25 highest ranked metabolites in original and sex-stratified models.

Supplementary Table 6a. Counts of imputed values by cohort. Columns indicated the number of values which were imputed for each metabolite in each cohort.

Metabolite	BLSA	TMCS
gly	0	0
trimethylaminenoxide	0	0
betaala	0	4
ala	0	0
sarcosine	0	43
aminoisobutyrate	0	15
ab	0	0
nndimethylglycine	0	0
choline	0	0
ser	0	0
creatinine	0	0
pro	0	0
guanidinoacetate	0	1
val	0	0
betaine	0	0
thr	0	0
taurine	0	0
pipecolate	0	3
hydroxyproline	0	0
creatine	0	0
ile	0	0
leu	0	0
asn	0	0
ornithine	0	0
asp	0	11

methylnicotinamide	5	89
prolinebetaine	1	8
gammabutyrobetaine	0	0
gln	0	0
lys	0	0
glu	0	0
met	0	0
triethanolamine	35	125
his	0	0
alphaaminoadipate	1	18
carnitine	0	0
phe	0	0
methylhistidine	0	0
arg	0	0
guanidinosuccinate	19	113
indole3acetate	3	28
citrulline	0	0
tyr	0	0
sdma	0	62
adma	0	54
oacetylcarnitine	36	47
trp	0	0
kynurenine	0	21
cystine	0	0
uridine	0	1
glycerophosphorylcholine	0	0
pyruvate	3	0
lactate	0	0
hydroxybutyrate_2	0	0
hydroxybutyrate_3	0	0

fumarate	1	41
oxoisopentanoate	1	0
hexanoate	4	4
succinate	0	0
oxoproline	0	0
methyl2oxopentanoate	0	0
acetylbutyrate	17	113
glutarate	54	10
malate	0	0
threonate	0	0
octanoate	30	25
oxoglutarate	27	2
pelargonate	2	0
terephthalate	10	21
urate	0	0
glycerophosphate	10	34
decanoate	0	34
cisaconitate	0	0
nacetylaspartate	3	3
hippurate	13	136
azelate	0	4
isocitrate	0	2
citrate	0	0
glucuronate	5	54
cysteinessulfate	13	174
mucate	0	0
indoxylsulfate	2	17

Supplementary Table 6b. Sensitivity analysis excluding imputed values for BLSA. The results shown represent logistic regression analysis with covariates of sex and age, as in the original analysis, with imputed values removed from analysis. * indicates that a

metabolite among the top 25 ranked metabolites in this analysis was also present among the top 25 highest ranked metabolites for BLSA in the original analysis.

Metabolites	MetS OR	MetS p-value	MetS FDR p-value	WC OR	WC p-value	WC FDR p-value	TG OR	TG p-value	TG FDR p-value	HDL OR	HDL p-value	HDL FDR p-value	BP OR	BP p-value	BP FDR p-value	Glucose OR	Glucose p-value	Glucose FDR p-value
urate*	2.86	1.55e-05	0.0012749	3.12	6.8e-06	0.0002781	1.79	0.0121316	0.110532	1.46	0.0835315	0.3542164	1.46	0.1016421	0.5953325	2.57	0.0001303	0.0001303
alphaaminoadipate*	2.48	4.06e-05	0.0016659	2.04	0.0010448	0.0154273	1.8	0.0068657	0.0703738	1.41	0.0836582	0.3542164	1.58	0.0278568	0.2855326	2.46	7.36e-05	7.36e-05
oxoglutarate*	2.18	0.0002003	0.0043297	1.89	0.0017471	0.0182641	2.01	0.0012834	0.0350804	1.98	0.0009175	0.0590666	1.21	0.3401824	0.807095	1.67	0.0113463	0.0113463
phe*	2.03	0.0002112	0.0043297	1.65	0.0063231	0.0345665	1.82	0.0020797	0.0426343	1.71	0.0034914	0.095431	1.24	0.2353416	0.72028	1.26	0.1859604	0.1859604
glu*	2.24	0.0003067	0.004775	2.3	0.000298	0.0081459	1.63	0.0248187	0.1356754	1.98	0.0014406	0.0590666	1.18	0.4472154	0.8994552	1.62	0.024978	0.024978
glucuronate*	2.02	0.0003494	0.004775	1.45	0.0483555	0.158606	1.76	0.0042913	0.0586482	1.34	0.1064271	0.3542164	1.48	0.0434141	0.3955502	1.65	0.0096971	0.0096971
hydroxybutyrate_2*	1.96	0.0004903	0.0057433	1.92	0.0008692	0.0154273	1.45	0.0507587	0.209999	1.45	0.0419114	0.2863942	1.6	0.0147153	0.2855326	2.18	0.0001256	0.0001256
cystine*	2.11	0.0008149	0.0083527	2.15	0.0011288	0.0154273	2.12	0.0011757	0.0350804	1.76	0.0063302	0.1203858	1.64	0.0219236	0.2855326	1.15	0.4951075	0.4951075
methyl2oxopentanoate*	2.05	0.0015337	0.0135009	2.01	0.0024752	0.0184518	1.75	0.0152324	0.1135504	1.57	0.038201	0.2847711	1.48	0.0820901	0.5177988	2.5	0.0001543	0.0001543
asn*	0.56	0.0016465	0.0135009	0.57	0.0020046	0.0182641	0.71	0.0599085	0.2339284	0.76	0.1164713	0.3542164	0.77	0.1411488	0.72028	0.94	0.7149429	0.7149429
ile*	1.96	0.0031686	0.0216553	1.99	0.0033765	0.0230725	1.38	0.1699463	0.4738501	1.5	0.0665351	0.3542164	0.98	0.9471096	0.9830758	1.92	0.0049199	0.0049199
leu*	1.93	0.0031691	0.0216553	1.81	0.0086406	0.0442829	1.55	0.0512193	0.209999	1.77	0.0088087	0.1203858	1.32	0.2105313	0.72028	1.64	0.0240218	0.0240218
oxoisopentanoate*	1.76	0.0036462	0.022999	1.83	0.0024743	0.0184518	1.64	0.0136646	0.1120496	1.31	0.1463558	0.3750367	1.67	0.0094109	0.2855326	1.82	0.0026977	0.0026977
gln*	0.55	0.0043607	0.0255414	0.63	0.0278492	0.1085488	0.76	0.1867839	0.4738501	0.84	0.38161	0.6258404	0.69	0.0672048	0.4934755	0.8	0.2865875	0.2865875
guanidinoacetate*	0.54	0.0068109	0.0372327	0.82	0.3833299	0.6687883	0.62	0.0352568	0.1700623	0.73	0.1419583	0.3750367	0.72	0.1516246	0.72028	1.15	0.5198416	0.5198416
carnitine*	1.66	0.0081634	0.0406283	1.25	0.2440806	0.5409355	1.56	0.0219207	0.1356754	1.29	0.1632407	0.4056283	1.63	0.0146088	0.2855326	1.62	0.0144279	0.0144279
tyr*	1.65	0.0084229	0.0406283	1.85	0.0018745	0.0182641	1.76	0.004079	0.0586482	1.36	0.0968901	0.3542164	1.28	0.1962907	0.72028	1.41	0.0712558	0.0712558

gly*	0.57	0.0109962	0.0500938	0.64	0.0424553	0.1450555	0.6	0.0247937	0.1356754	0.93	0.7252369	0.8583158	0.61	0.0275791	0.2855326	1.03	0.9075403	0.9075403
uridine*	1.62	0.0155648	0.0653887	1.26	0.2546814	0.5495756	2	0.0011668	0.0350804	1.63	0.013614	0.1594784	1.08	0.7023314	0.9562838	1.47	0.0553605	0.0553605
pro*	1.62	0.0159485	0.0653887	1.45	0.0680407	0.2066422	1.37	0.1281642	0.393132	1.36	0.1166322	0.3542164	1.65	0.0154208	0.2855326	1.83	0.0036466	0.0036466
ala*	1.59	0.0215372	0.0840977	1.84	0.0044653	0.0281657	1.31	0.1850624	0.4738501	1.27	0.2107447	0.454765	1.05	0.7949921	0.9562838	1.71	0.0105376	0.0105376
pyruvate*	1.48	0.022814	0.085034	1.65	0.0056176	0.0329032	1.48	0.0293109	0.1502183	1.24	0.1971317	0.454765	1.1	0.5893669	0.9529254	1.32	0.1124355	0.1124355
isocitrate*	1.55	0.0272106	0.0970115	1.27	0.2310166	0.5355591	1.22	0.3266301	0.5777335	0.97	0.8887592	0.9448948	1.59	0.0240846	0.2855326	1.06	0.7579231	0.7579231
lactate*	1.61	0.0288757	0.0986586	3.16	6.6e-06	0.0002781	1.24	0.3273595	0.5777335	1.6	0.0290942	0.2847711	1.17	0.4671827	0.8994552	1.79	0.0109241	0.0109241
val*	1.51	0.0384247	0.1250204	1.57	0.0291228	0.1085488	1.45	0.0731848	0.2727798	1.38	0.0995825	0.3542164	1.19	0.397656	0.8812916	1.66	0.0140339	0.0140339
asp	1.44	0.0396406	0.1250204	1.41	0.0559126	0.1763398	1.18	0.3489762	0.5777335	1.45	0.0360764	0.2847711	0.97	0.885247	0.9562838	1.01	0.9401103	0.9401103
taurine	0.7	0.0487181	0.1479588	0.87	0.4479851	0.6815623	0.85	0.3817128	0.6019317	1.17	0.3804769	0.6258404	0.87	0.4693061	0.8994552	0.74	0.1002705	0.1002705
kynurenine	1.42	0.059985	0.1756703	1.5	0.036755	0.1310396	1.4	0.0836536	0.2982434	1.32	0.1263946	0.3573915	1.42	0.0722159	0.4934755	0.97	0.8737608	0.8737608
betaala	1.45	0.066824	0.1889505	1.23	0.3170903	0.6460956	1.24	0.2995782	0.5777335	1.25	0.270682	0.5101389	1.2	0.3910166	0.8812916	1.37	0.1234898	0.1234898
glycerophosphate	0.64	0.0894588	0.2445208	0.52	0.0167874	0.0724509	0.63	0.0955164	0.3263476	1.01	0.9575356	0.969357	0.94	0.8090927	0.9562838	1.04	0.8914283	0.8914283
glutarate	0.66	0.0940927	0.2488905	0.99	0.9580954	0.9801988	0.81	0.4003781	0.6186894	0.77	0.2737331	0.5101389	1.33	0.2547332	0.72028	0.78	0.3050819	0.3050819
creatinine	1.54	0.0989546	0.2535711	1.01	0.9682452	0.9801988	1.35	0.2736941	0.5473882	0.96	0.8657615	0.9448948	1.36	0.2516465	0.72028	1.05	0.8408483	0.8408483
methylhistidine	1.36	0.1035021	0.257187	0.96	0.8247282	0.9163014	1.48	0.0468146	0.209999	1.2	0.3292477	0.5744321	1.27	0.2237699	0.72028	1.28	0.2009708	0.2009708
hydroxyproline	1.35	0.1172216	0.2827109	1.29	0.1873698	0.4801352	1.1	0.6195232	0.7902606	0.85	0.3950907	0.6352439	1.45	0.0597757	0.4901607	1.36	0.1280557	0.1280557
oxoproline	0.73	0.1226328	0.2873112	0.89	0.5824415	0.7630863	0.61	0.02331	0.1356754	0.78	0.2195247	0.4615648	1.04	0.8589644	0.9562838	1.29	0.2312121	0.2312121
azelate	4.26	0.1774736	0.4042455	0.99	0.9926722	0.9926722	6.42	0.0996082	0.3267151	2.32	0.4378325	0.6527685	1.8	0.5926731	0.9529254	10.02	0.0383527	0.0383527
indoxylsulfate	1.26	0.2148111	0.4760677	1.21	0.3019977	0.6349695	1.27	0.2237079	0.5091814	1.1	0.5901207	0.7644014	1.07	0.7264501	0.9562838	1.21	0.3242103	0.3242103
hydroxybutyrate_3	1.25	0.2361668	0.4967741	1.07	0.7388596	0.8533308	1.29	0.1906958	0.4738501	1.16	0.436311	0.6527685	1.06	0.7551738	0.9562838	1.36	0.1169592	0.1169592

nndimethylglycine	1.25	0.2362706	0.4967741	1.32	0.1553875	0.4306571	1.28	0.2100594	0.5066139	1.38	0.0956774	0.3542164	1.08	0.7140729	0.9562838	1.33	0.1536487	0.1536487
creatine	1.29	0.2436225	0.4994261	1.17	0.4664378	0.6829982	1.11	0.6267624	0.7902606	1.19	0.4052188	0.6389989	1.37	0.1580975	0.72028	2.01	0.0030579	0.0030579
nacetylaspartate	0.83	0.3085343	0.6170685	0.86	0.4209444	0.6778678	0.58	0.0063533	0.0703738	0.61	0.008064	0.1203858	1.1	0.6212288	0.9562838	0.96	0.8490718	0.8490718
mucate	0.84	0.3490046	0.68139	0.89	0.5440174	0.7534025	1	0.989196	0.989196	0.98	0.9103255	0.9448948	1.24	0.2784016	0.7609644	0.77	0.1898986	0.1898986
malate	1.19	0.4160483	0.7911243	1.66	0.025703	0.1053825	0.78	0.2632299	0.5396213	1.1	0.6434038	0.7991585	1.15	0.533362	0.9507757	1.71	0.0186557	0.0186557
trp	1.17	0.4245057	0.7911243	1.1	0.6249143	0.7648205	1.21	0.3447414	0.5777335	1.39	0.0934689	0.3542164	1.16	0.4716655	0.8994552	1.34	0.1467392	0.1467392
lys	0.86	0.4393884	0.8006633	0.76	0.1575575	0.4306571	1.02	0.9082876	0.9354441	1.05	0.8154076	0.9159373	1.05	0.814966	0.9562838	1.12	0.5581157	0.5581157
acetylbutyrate	1.15	0.4622508	0.8240122	0.97	0.878973	0.925607	1.17	0.4312181	0.642907	1.03	0.8893359	0.9448948	1	0.9942161	0.9942161	1.17	0.4169568	0.4169568
arg	1.14	0.4782816	0.8344487	1.32	0.1642071	0.4343544	0.91	0.6231361	0.7902606	0.79	0.2103075	0.454765	1.06	0.7634363	0.9562838	1.72	0.0079272	0.0079272
succinate	1.16	0.4903142	0.8376202	1.73	0.0152019	0.0724509	0.99	0.9545284	0.9663127	0.92	0.68518	0.8262465	1.36	0.1716971	0.72028	1.33	0.1984308	0.1984308
ab	1.12	0.5571995	0.8767424	1.12	0.5512701	0.7534025	1.16	0.4397447	0.6439119	1.27	0.2028021	0.454765	1.12	0.5468875	0.9529254	1.76	0.005708	0.005708
octanoate	0.89	0.558246	0.8767424	0.84	0.3830223	0.6687883	0.84	0.4074296	0.6186894	0.7	0.0750137	0.3542164	0.97	0.8861174	0.9562838	1.35	0.1479046	0.1479046
betaine	1.11	0.5682087	0.8767424	0.84	0.3390809	0.6466194	1.26	0.2253792	0.5091814	1.23	0.2565532	0.5080342	1.04	0.8296445	0.9562838	0.97	0.8911876	0.8911876
cisaconitate	1.11	0.5773316	0.8767424	1.1	0.6068133	0.7630863	0.95	0.7723946	0.9048051	0.8	0.2036275	0.454765	1.29	0.1757319	0.72028	1.11	0.5635985	0.5635985
adma	0.9	0.5835007	0.8767424	1.28	0.2031784	0.5048675	1.04	0.8437136	0.9354441	1.12	0.5471842	0.7574662	1	0.9888073	0.9942161	0.85	0.4112826	0.4112826
thr	0.91	0.5901895	0.8767424	0.96	0.8308697	0.9163014	0.98	0.9126284	0.9354441	0.91	0.5780688	0.7644014	1.2	0.2987126	0.790143	1.11	0.5342217	0.5342217
decanoate	1.1	0.6085615	0.8767424	0.88	0.507233	0.7171225	1.1	0.6251906	0.7902606	0.86	0.4146538	0.6415399	1.13	0.5328012	0.9507757	1.21	0.3406765	0.3406765
fumarate	1.1	0.625998	0.8767424	1.37	0.1346424	0.39431	0.96	0.8298361	0.9354441	1.21	0.3270308	0.5744321	1.05	0.8285653	0.9562838	1.19	0.3912086	0.3912086
ornithine	1.1	0.6302656	0.8767424	1.19	0.3738122	0.6687883	0.97	0.8966096	0.9354441	1.5	0.0324277	0.2847711	0.99	0.9373607	0.9830758	1.16	0.4346935	0.4346935
cysteinessulfate	1.18	0.6321721	0.8767424	1.13	0.7112498	0.8331784	0.82	0.553971	0.7699257	1.66	0.1242991	0.3573915	0.85	0.615611	0.9562838	1.15	0.7008306	0.7008306
indole3acetate	1.09	0.6390022	0.8767424	1.62	0.0165376	0.0724509	1.23	0.3073066	0.5777335	0.91	0.6059279	0.7644014	0.99	0.9649766	0.9891011	1.18	0.399878	0.399878

hippurate	0.92	0.645325	0.8767424	1.04	0.8416125	0.9163014	0.97	0.8640585	0.9354441	0.82	0.2602126	0.5080342	1.11	0.5687646	0.9529254	1.02	0.9136595	0.9136595
prolinebetaine	0.92	0.6522108	0.8767424	1.16	0.4759311	0.6846728	0.79	0.2359621	0.5091814	0.95	0.8008471	0.912849	0.91	0.6540075	0.9562838	0.87	0.4885932	0.4885932
triethanolamine	1.1	0.670497	0.8867864	1.04	0.8492549	0.9163014	1.25	0.3159778	0.5777335	1.42	0.1050324	0.3542164	0.76	0.2478779	0.72028	1.22	0.3590953	0.3590953
terephthalate	0.93	0.6834573	0.8895794	0.86	0.4135378	0.6778678	0.88	0.5248658	0.7420517	1.05	0.801526	0.912849	0.95	0.7982044	0.9562838	1.09	0.6396332	0.6396332
his	0.93	0.7028794	0.8955949	0.91	0.5880905	0.7630863	1.31	0.1440646	0.4219034	1.12	0.5310666	0.7574662	1.19	0.3414171	0.807095	1.17	0.3814172	0.3814172
citrulline	0.93	0.7215774	0.8955949	0.84	0.4298674	0.6778678	1.12	0.5937074	0.7902606	0.93	0.7327086	0.8583158	0.78	0.2522501	0.72028	1.23	0.3472109	0.3472109
gammabutyrobetaine	0.93	0.7274059	0.8955949	0.79	0.2351235	0.5355591	0.97	0.8752962	0.9354441	0.82	0.3043923	0.5546704	0.87	0.4961141	0.9245763	1.04	0.8516508	0.8516508
sarcosine	0.94	0.7368695	0.8955949	0.85	0.3787975	0.6687883	1.24	0.2338017	0.5091814	1.09	0.6018628	0.7644014	1.05	0.7881221	0.9562838	1.18	0.3613233	0.3613233
hexanoate	0.94	0.7498897	0.8955949	0.91	0.6343239	0.76492	0.77	0.1771609	0.4738501	0.76	0.1382603	0.3750367	1.27	0.2149265	0.72028	1.2	0.3318062	0.3318062
sdma	0.93	0.7536104	0.8955949	0.81	0.333843	0.6466194	1.23	0.3593221	0.5777335	1.13	0.5542436	0.7574662	0.85	0.4611296	0.8994552	0.89	0.6115819	0.6115819
pipecolate	1.05	0.7884579	0.9087731	0.87	0.4488337	0.6815623	1.19	0.3586824	0.5777335	1	0.9906333	0.9906333	1.21	0.3223697	0.807095	1.04	0.832573	0.832573
threonate	0.95	0.7961146	0.9087731	0.97	0.8807766	0.925607	1.27	0.2511444	0.5280471	1.4	0.0981808	0.3542164	0.92	0.6792021	0.9562838	1.01	0.9434764	0.9434764
ser	1.05	0.8046092	0.9087731	0.91	0.6720198	0.7986323	0.91	0.676086	0.8274485	1.45	0.0829508	0.3542164	0.74	0.1776072	0.72028	1.16	0.4993865	0.4993865
methylnicotinamide	1.05	0.8090297	0.9087731	0.79	0.2245929	0.5355591	1.35	0.1294459	0.393132	1.02	0.9348369	0.9582079	1.04	0.8501093	0.9562838	0.78	0.1935448	0.1935448
pelargonate	0.95	0.8689577	0.9626348	1.28	0.4288221	0.6778678	1.27	0.4521972	0.6505293	0.85	0.5831674	0.7644014	0.74	0.3444918	0.807095	1.59	0.1335259	0.1335259
oacetylcarnitine	1.03	0.9025155	0.9626348	0.85	0.462637	0.6829982	1.08	0.7108199	0.8447425	0.97	0.9023382	0.9448948	0.93	0.7533601	0.9562838	1.24	0.3324363	0.3324363
met	0.97	0.9031672	0.9626348	0.89	0.6054908	0.7630863	0.97	0.894332	0.9354441	0.91	0.652971	0.7991585	1.07	0.761834	0.9562838	1.24	0.3356561	0.3356561
aminoisobutyrate	1.02	0.9143648	0.9626348	0.97	0.8917433	0.925607	1.11	0.6138026	0.7902606	1.26	0.2558316	0.5080342	1.16	0.4619424	0.8994552	0.88	0.5487635	0.5487635
trimethylaminenoxide	0.98	0.9193957	0.9626348	1.11	0.5678517	0.7630863	0.92	0.6360634	0.7902606	0.88	0.4522394	0.6622078	0.98	0.9235115	0.9830758	1.04	0.8460522	0.8460522
guanidinosuccinate	1.02	0.9274164	0.9626348	0.9	0.6141914	0.7630863	0.97	0.9006742	0.9354441	0.83	0.3422994	0.5847615	0.96	0.8415842	0.9562838	0.9	0.5927931	0.5927931
citrate	1.01	0.9528537	0.9752693	0.83	0.3230478	0.6460956	0.93	0.7088063	0.8447425	0.75	0.1149588	0.3542164	1.11	0.5920816	0.9529254	0.84	0.3611277	0.3611277

glycerophosphorylcholine	1.01	0.9633757	0.9752693	0.95	0.8154153	0.9163014	0.96	0.8492481	0.9354441	0.89	0.5519454	0.7574662	0.97	0.8863118	0.9562838	1.2	0.3623529	0.3623529
choline	1	0.991028	0.991028	1.28	0.4089838	0.6778678	0.75	0.3444388	0.5777335	1.04	0.9035426	0.9448948	1.15	0.6541611	0.9562838	1.24	0.4652082	0.4652082

Supplementary Table 6c. Sensitivity analysis excluding imputed values for TMCS. The results shown represent logistic regression analysis with covariates of sex and age, as in the original analysis, with imputed values removed from analysis. * indicates that a metabolite among the top 25 ranked metabolites in this analysis was also present among the top 25 highest ranked metabolites for TMCS in the original analysis.

Metabolites	MetS OR	MetS p-value	MetS FDR p-value	WC OR	WC p-value	WC FDR p-value	TG OR	TG p-value	TG FDR p-value	HDL OR	HDLp-value	HDL FDR p-value	BP OR	BP p-value	BP FDR p-value	Glucose OR	Glucose p-value	Glucose FDR p-value
pro*	2.22	0	2e-07	1.79	6.32e-05	0.0007683	1.61	0.0001991	0.0040822	1.57	0.000723	0.0197634	1.34	0.0265016	0.1552239	1.94	2e-07	2e-07
glu*	2.62	0	0	2.36	0	3e-07	2.16	0	2e-07	1.89	1.6e-06	0.0001283	1.12	0.3523812	0.6234465	1.57	0.0001596	0.0001596
methyl2oxopentanoate*	2.18	0	4e-07	1.46	0.0101364	0.0433872	1.76	2.07e-05	0.0008476	1.51	0.0026004	0.0333038	1.48	0.0043193	0.0698039	1.83	3.2e-06	3.2e-06
ile*	2.15	1e-07	2.7e-06	1.89	7.5e-05	0.0007683	1.63	0.0004407	0.0060229	1.62	0.0010778	0.0220948	1.14	0.3469516	0.6234465	1.52	0.0016959	0.0016959
mucate*	0.53	2e-07	2.7e-06	0.54	8.1e-06	0.0002223	0.69	0.0016342	0.0121819	0.73	0.008216	0.0842138	0.61	9.65e-05	0.007535	0.72	0.0035501	0.0035501
leu*	2.07	7e-07	9.2e-06	2.08	6.7e-06	0.0002223	1.62	0.0007301	0.0073111	1.56	0.002843	0.0333038	1.18	0.2569146	0.5693784	1.66	0.0002218	0.0002218
cystine*	1.84	1.1e-06	1.23e-05	1.37	0.0194199	0.0723833	1.5	0.000857	0.0073111	1.77	1.39e-05	0.0005712	1.37	0.0110338	0.1130964	1.43	0.0022987	0.0022987
alphaaminoadipate*	1.88	1.9e-06	1.9e-05	1.67	0.0004774	0.0035589	1.43	0.0041677	0.0201029	1.35	0.0225745	0.1494896	1.26	0.0736219	0.2982323	1.92	3e-07	3e-07
oxoisopentanoate*	1.74	2.7e-06	2.47e-05	1.43	0.0049633	0.0254368	1.43	0.0020456	0.0139784	1.24	0.0748419	0.2454814	1.19	0.1356267	0.4448557	1.68	5.1e-06	5.1e-06
ala*	1.78	1.04e-05	8.27e-05	1.81	6.67e-05	0.0007683	1.53	0.0008916	0.0073111	1.29	0.0594141	0.2205178	1.17	0.2397012	0.545986	2.1	0	0
ser*	0.6	1.15e-05	8.27e-05	0.62	0.0001547	0.0014096	0.68	0.0006696	0.0073111	0.92	0.4525585	0.6510491	0.72	0.0051076	0.0698039	0.93	0.5288011	0.5288011
val*	1.8	1.21e-05	8.27e-05	1.86	4.26e-05	0.0006993	1.41	0.0084696	0.0385836	1.29	0.0626118	0.2205178	1.15	0.2946837	0.5921807	1.94	4e-07	4e-07

gly*	0.6	1.91e-05	0.0001206	0.83	0.1525352	0.3573682	0.66	0.0003288	0.0053929	0.79	0.0502224	0.2059116	0.87	0.2240926	0.545986	0.81	0.0621333	0.0621333
pyruvate*	1.65	4.21e-05	0.0002467	1.18	0.2039538	0.3800956	1.58	0.000156	0.0040822	1.31	0.0309918	0.1494896	1.16	0.2380181	0.545986	1.37	0.0068198	0.0068198
phe*	1.65	6.54e-05	0.0003565	1.57	0.0013954	0.0076283	1.26	0.0592481	0.1428925	1.29	0.0487467	0.2059116	1.17	0.2107374	0.545986	1.48	0.0011762	0.0011762
tyr*	1.65	6.96e-05	0.0003565	1.82	3.11e-05	0.0006379	1.2	0.132013	0.2577396	1.13	0.3252522	0.5674613	1.08	0.527372	0.8027603	1.63	6.71e-05	6.71e-05
guanidinosuccinate*	0.63	0.0005479	0.0025038	0.62	0.0011991	0.007023	0.74	0.019433	0.0724322	0.65	0.0022874	0.0333038	0.93	0.5791409	0.8160899	0.75	0.0205194	0.0205194
trp*	1.57	0.0005496	0.0025038	1.38	0.0280827	0.0852881	1.39	0.010788	0.0444043	1.22	0.1387539	0.3670264	0.85	0.2369036	0.545986	1.24	0.0843374	0.0843374
gln*	0.67	0.0007675	0.0033124	0.72	0.0121765	0.0475464	0.7	0.0023343	0.0143	0.89	0.3396313	0.5683625	0.63	0.0001838	0.007535	1.13	0.269	0.269
hydroxybutyrate_3*	0.66	0.001037	0.0042518	0.62	0.0008483	0.0054861	0.68	0.0024415	0.0143	0.77	0.0463788	0.2059116	1.13	0.3392838	0.6234465	0.89	0.3506441	0.3506441
isocitrate*	1.42	0.0015816	0.0061759	1.27	0.058854	0.1664149	1.32	0.0108303	0.0444043	1.33	0.01407	0.1281937	1.25	0.0492889	0.2245385	1.2	0.0830357	0.0830357
hydroxybutyrate_2*	1.42	0.0016615	0.0061929	1.05	0.6627484	0.7991966	1.29	0.0209631	0.0747379	1.2	0.1129454	0.3188162	1.21	0.0992081	0.3536984	1.49	0.0002462	0.0002462
cisaconitate*	1.38	0.0034581	0.0123289	1.23	0.0961581	0.2464052	1.2	0.0937353	0.1970846	1.29	0.0269692	0.1494896	1.35	0.0100384	0.1130964	1.26	0.0276847	0.0276847
lactate*	1.38	0.0038706	0.0132247	1.25	0.072924	0.1993257	1.25	0.0414458	0.1132852	1.08	0.5055387	0.7026131	1.27	0.0417887	0.2119818	1.48	0.0003441	0.0003441
acetylbutyrate	0.7	0.004617	0.0151438	0.83	0.1836825	0.3800956	0.84	0.1558634	0.2904728	0.87	0.2672545	0.5217826	0.65	0.0016224	0.03326	0.84	0.1329181	0.1329181
carnitine	1.41	0.0048575	0.0153199	1.13	0.3781655	0.5742513	1.3	0.0316049	0.0959851	1.17	0.2243061	0.4840289	1.33	0.0253736	0.1552239	1.31	0.0213755	0.0213755
urate	1.41	0.0082618	0.0250913	1.38	0.0254302	0.0802029	1.12	0.361632	0.4861282	1.05	0.7152676	0.8260837	1.55	0.0012661	0.03326	1.23	0.0910825	0.0910825
prolinebetaine	0.73	0.0085994	0.025184	0.99	0.9423483	0.9678252	0.77	0.0279537	0.0914556	0.86	0.2164228	0.4796398	0.97	0.7924101	0.9069173	0.87	0.2056081	0.2056081
decanoate	0.77	0.010226	0.0287393	1.05	0.6803817	0.8085696	0.82	0.0511519	0.135305	0.88	0.2377087	0.4997978	0.96	0.6948777	0.8766149	0.82	0.0448768	0.0448768
citrate	0.74	0.0105144	0.0287393	0.88	0.3316609	0.5332587	0.81	0.0653865	0.1531912	0.86	0.213209	0.4796398	0.99	0.9211423	0.9325144	0.98	0.8502059	0.8502059
hippurate	0.74	0.0160418	0.0424332	0.96	0.791985	0.8921048	0.78	0.0551021	0.1411273	0.83	0.1720183	0.4407968	0.9	0.4335826	0.7255871	0.99	0.9148568	0.9148568
gammabutyrobetaine	0.75	0.0169674	0.043479	0.83	0.1766324	0.3800956	0.79	0.0567951	0.1411273	0.91	0.4402491	0.6446505	1.09	0.4766899	0.7664426	0.96	0.7365369	0.7365369
met	1.27	0.0274465	0.0682004	1.19	0.1587971	0.3617045	1.21	0.0694759	0.15525	1.29	0.0303441	0.1494896	1.2	0.0871191	0.3247167	1.06	0.5353396	0.5353396

asn	0.76	0.0338922	0.0797843	0.6	0.0004094	0.0033573	0.76	0.0353354	0.1034822	0.9	0.4378067	0.6446505	0.73	0.0218201	0.1552239	1.11	0.3981662	0.3981662
choline	1.3	0.0340543	0.0797843	1.23	0.1321154	0.3186312	1.13	0.3188734	0.450821	0.95	0.712924	0.8260837	1.34	0.0235181	0.1552239	1.14	0.2816198	0.2816198
betaine	0.79	0.0372135	0.084764	0.66	0.0008697	0.0054861	0.87	0.2259034	0.3632172	0.94	0.6155513	0.7886751	1.02	0.8489268	0.9069173	0.84	0.102113	0.102113
threonate	0.78	0.038376	0.0850496	0.69	0.0060225	0.0290496	0.83	0.1155051	0.2367854	0.88	0.3003408	0.5353901	0.78	0.0439474	0.2119818	0.87	0.2300136	0.2300136
aminoisobutyrate	0.78	0.0410745	0.0886345	0.78	0.0882463	0.2334256	0.7	0.004075	0.0201029	0.78	0.0534971	0.2088932	1.06	0.662655	0.8490267	0.97	0.8242875	0.8242875
methylnicotinamide	1.28	0.0450899	0.0948044	1.19	0.2018415	0.3800956	1.31	0.0289981	0.0914556	1.23	0.1166401	0.3188162	0.93	0.5977087	0.8160899	1.2	0.1329638	0.1329638
creatine	1.27	0.0554638	0.1137007	1.39	0.0214409	0.0736947	1.3	0.0371503	0.1050457	1.15	0.2806597	0.5230477	1.14	0.3233947	0.6167062	1.16	0.2205363	0.2205363
adma	0.8	0.0636745	0.1273489	1.11	0.4649452	0.6808127	0.7	0.0030676	0.0167694	0.8	0.082509	0.2505828	1.1	0.4508252	0.7393534	0.85	0.1533879	0.1533879
thr	1.25	0.0678853	0.132538	1.1	0.4759533	0.6847048	1.19	0.1494495	0.2849968	1.33	0.0290924	0.1494896	1.08	0.5451125	0.8055936	1.26	0.0457838	0.0457838
succinate	0.81	0.0739253	0.1409738	0.71	0.0074314	0.0338543	0.82	0.0850456	0.1835195	0.86	0.2047318	0.4796398	0.98	0.8849366	0.9185418	1.01	0.9542538	0.9542538
glucuronate	1.22	0.0950145	0.1770724	1.24	0.1054853	0.2621149	1.18	0.1652911	0.2946493	1.26	0.0645418	0.2205178	1.2	0.1506191	0.4574358	1.09	0.4385681	0.4385681
glycerophosphorylcholine	1.21	0.1009621	0.1839753	0.88	0.338883	0.5343924	1.16	0.1995742	0.3339813	1.08	0.4997732	0.7026131	1.24	0.0734745	0.2982323	0.96	0.6774421	0.6774421
glycerophosphate	1.19	0.1137346	0.2027444	1.07	0.6008137	0.7464655	1.3	0.018749	0.0724322	1.23	0.0815494	0.2505828	1.04	0.722646	0.8844324	0.82	0.070337	0.070337
uridine	0.86	0.1744223	0.3043113	1.08	0.5305258	0.709257	0.9	0.3315009	0.4530512	1.02	0.8737165	0.8955594	0.88	0.2855774	0.5921807	0.92	0.4432149	0.4432149
cysteinessulfate	1.2	0.188952	0.3193246	0.92	0.5994819	0.7464655	1.08	0.5928102	0.7044991	1.4	0.0218005	0.1494896	0.98	0.9147315	0.9325144	1.07	0.6065328	0.6065328
pipecolate	1.17	0.1908159	0.3193246	0.97	0.8064285	0.89361	0.93	0.5546611	0.6891244	0.89	0.3607126	0.5915687	1.08	0.5501615	0.8055936	1.36	0.0098608	0.0098608
terephthalate	0.86	0.2098421	0.344141	1.07	0.593553	0.7464655	0.91	0.4016562	0.5312227	0.99	0.9606292	0.9724888	0.84	0.1491383	0.4574358	0.89	0.3144935	0.3144935
trimethylaminenoxide	0.87	0.2199177	0.3535932	0.92	0.4977627	0.6918058	0.85	0.1613197	0.2939603	0.85	0.1784351	0.4433842	0.93	0.567839	0.8160899	1.07	0.5435934	0.5435934
hydroxyproline	1.13	0.3010839	0.4747862	0.99	0.9362399	0.9678252	1.02	0.8945186	0.9284877	0.97	0.7775163	0.8500845	1.01	0.939515	0.939515	0.99	0.9043927	0.9043927
taurine	1.12	0.3129439	0.4841774	1.39	0.0105822	0.0433872	1.13	0.2963122	0.4338857	1.32	0.0215621	0.1494896	1.14	0.2861858	0.5921807	0.96	0.7423042	0.7423042
ornithine	1.12	0.3448519	0.5153365	1.02	0.902117	0.9678252	0.96	0.6991464	0.7962501	1.02	0.8667852	0.8955594	0.94	0.5933707	0.8160899	1.08	0.4882293	0.4882293

his	1.12	0.3456525	0.5153365	1.19	0.2035541	0.3800956	1.14	0.2697272	0.4095857	1.05	0.6808693	0.809149	0.97	0.8233662	0.9069173	1.08	0.5260447	0.5260447
sdma	0.9	0.3591603	0.5217143	1.04	0.7832101	0.8921048	0.77	0.0284933	0.0914556	0.75	0.0270897	0.1494896	1.06	0.6413932	0.8348293	1.02	0.8482213	0.8482213
oxoproline	0.9	0.362655	0.5217143	0.87	0.2736673	0.4675149	0.85	0.1785891	0.311581	1.08	0.5285346	0.7223306	1.03	0.7941699	0.9069173	1.1	0.3869229	0.3869229
asp	1.1	0.3869625	0.5357192	1.17	0.1952792	0.3800956	1.11	0.3290012	0.4530512	1.03	0.8291434	0.8829839	0.94	0.6215293	0.8220227	1.03	0.8096231	0.8096231
creatinine	0.88	0.3897822	0.5357192	1	0.9834402	0.9955814	0.79	0.1275268	0.2550535	0.88	0.435452	0.6446505	1.22	0.2199284	0.545986	0.94	0.6796011	0.6796011
octanoate	0.91	0.3919897	0.5357192	0.85	0.1968708	0.3800956	0.98	0.8577731	0.9036663	1.11	0.3798163	0.6106851	1.18	0.1576256	0.4616179	0.88	0.2545239	0.2545239
hexanoate	0.92	0.4610588	0.619784	1.08	0.5409237	0.709257	0.86	0.1857352	0.3172976	0.93	0.5658791	0.7365411	0.98	0.8503212	0.9069173	1	0.9798578	0.9798578
oxoglutarate	1.08	0.4724216	0.6248157	0.86	0.2275573	0.4056456	1.12	0.2894808	0.4315895	1.14	0.2591541	0.5183082	1.08	0.5236159	0.8027603	1	0.9666519	0.9666519
kynurenine	1.08	0.522501	0.6800807	1.38	0.0217793	0.0736947	1.01	0.9640885	0.9640885	0.97	0.8447992	0.8881223	1.05	0.7221266	0.8844324	0.86	0.199052	0.199052
oacetylcarnitine	1.07	0.5414702	0.6937587	1.08	0.5434203	0.709257	1.14	0.2667558	0.4095857	0.91	0.4291698	0.6446505	1.17	0.2020651	0.545986	1.14	0.2510517	0.2510517
methylhistidine	1.07	0.5592648	0.7029518	0.85	0.2185219	0.3981955	0.94	0.5669478	0.691207	0.88	0.2991676	0.5353901	1.35	0.0193551	0.1552239	0.98	0.8395736	0.8395736
triethanolamine	1.08	0.5657905	0.7029518	1.14	0.3681667	0.5696164	0.95	0.7095834	0.7970663	1.04	0.7448091	0.844346	1.09	0.528647	0.8027603	0.98	0.8602508	0.8602508
fumarate	0.94	0.5884871	0.7158528	0.76	0.038538	0.1128613	0.95	0.6793217	0.7845687	1	0.9900988	0.9900988	1.02	0.8816858	0.9185418	1	0.99742	0.99742
betaala	0.93	0.5936341	0.7158528	1	0.9979192	0.9979192	0.86	0.2340482	0.3690759	0.88	0.3390235	0.5683625	1.03	0.8340677	0.9069173	1.13	0.3336538	0.3336538
lys	1.06	0.6478354	0.7698914	1.17	0.2478711	0.432456	1.08	0.5207917	0.6569988	1.05	0.6716016	0.809149	0.76	0.0334432	0.1828229	1.03	0.8210561	0.8210561
arg	0.96	0.7253828	0.8452632	0.93	0.544917	0.709257	0.81	0.0700518	0.15525	0.9	0.4045299	0.6379125	0.94	0.6070913	0.8160899	1.12	0.3135873	0.3135873
sarcosine	1.04	0.7400809	0.8452632	1.07	0.6281532	0.7687845	0.91	0.4578361	0.5959136	0.87	0.2586679	0.5183082	0.96	0.7410147	0.8935766	0.97	0.7656861	0.7656861
ab	0.96	0.7421823	0.8452632	1.01	0.9108972	0.9678252	0.89	0.3138768	0.450821	0.86	0.2164228	0.4796398	1.03	0.8262343	0.9069173	1.35	0.006937	0.006937
citrulline	0.96	0.7532621	0.84613	0.74	0.0224679	0.0736947	0.92	0.4668591	0.5981632	1.06	0.6498423	0.809149	1.24	0.0763766	0.2982323	1.04	0.7246392	0.7246392
nndimethylglycine	1.03	0.7927607	0.872465	0.99	0.9442197	0.9678252	0.98	0.859585	0.9036663	1.05	0.6641702	0.809149	1.21	0.1307437	0.4448557	0.87	0.2274901	0.2274901
azelate	1.03	0.7997099	0.872465	0.92	0.4976907	0.6918058	1.02	0.8563819	0.9036663	1.07	0.5400852	0.7251696	1.02	0.8516175	0.9069173	0.97	0.7499186	0.7499186

nacetylaspartate	0.97	0.8086261	0.872465	0.87	0.2862458	0.4790235	0.99	0.9480924	0.963076	1.04	0.7665218	0.849389	1.13	0.3073078	0.599982	0.89	0.2889727	0.2889727
pelargonate	1.02	0.8390204	0.8935023	0.84	0.1767205	0.3800956	1.06	0.5731961	0.691207	1.07	0.548299	0.7251696	1.17	0.1932492	0.545986	0.94	0.56432	0.56432
indole3acetate	0.98	0.9037027	0.9500465	1.04	0.7941908	0.8921048	0.94	0.6570423	0.7696781	0.97	0.7991892	0.8622831	0.96	0.7916894	0.9069173	0.96	0.7711944	0.7711944
indoxylsulfate	0.99	0.9193628	0.9542753	1.02	0.8526464	0.9322267	1.02	0.840697	0.9036663	1.05	0.6567529	0.809149	0.88	0.2960904	0.5921807	0.93	0.5242008	0.5242008
malate	1.01	0.9530853	0.9769124	0.96	0.7266928	0.8512687	0.88	0.2243561	0.3632172	0.83	0.1118293	0.3188162	1.33	0.013918	0.1268087	1.29	0.0171709	0.0171709
glutarate	1	0.9764001	0.9884544	0.89	0.3238293	0.5310801	0.99	0.9513312	0.963076	1.13	0.2771713	0.5230477	1.1	0.4047699	0.6914818	0.97	0.784439	0.784439
guanidinoacetate	1	0.9947685	0.9947685	0.89	0.4414985	0.6582341	1.04	0.8045821	0.8915639	1.05	0.7516738	0.844346	1.15	0.3573413	0.6234465	1.06	0.6793411	0.6793411
